# Cembrane-Based Diterpenoids Isolated from the Soft Coral *Sarcophyton* sp.

**DOI:** 10.3390/md23110422

**Published:** 2025-10-30

**Authors:** Yueping Wang, Xiaohui Li, Yusen Guo, Te Li, Xia Yan, Han Ouyang, Wenhan Lin, Bin Wu, Hongyu Hu, Shan He

**Affiliations:** 1 Li Dak Sum Yip Yio Chin Kenneth Li Marine Biopharmaceutical Research Center, Ningbo University, Ningbo 315211, China; wangyueping2024@163.com (Y.W.); lixiaohui@nbu.edu.cn (X.L.); g2286194566@126.com (Y.G.); telinbu@163.com (T.L.); yanxia@nbu.edu.cn (X.Y.); 2 Institute of Drug Discovery Technology, Ningbo University, Ningbo 315211, China; ouyanghan@nbu.edu.cn; 3 Ningbo Institute of Marine Medicine, Peking University, Ningbo 315800, China; whlin@bjmu.edu.cn; 4 Ocean College, Zhejiang University, Hangzhou 310058, China; wubin@zju.edu.cn; 5 Xingzhi College, Zhejiang Normal University, Lanxi 321004, China

**Keywords:** soft coral, *Sarcophyton* sp., marine natural products, cembranoids, antibacterial activity

## Abstract

This research explored the chemical composition of the soft coral *Sarcophyton* sp., leading to the discovery of six previously unreported cembranoids, sarcophynoids D–I (**1**–**6**), and three known analog compounds (**7**–**9**). Structural elucidation of the new metabolites was achieved by spectroscopic methods, including one- and two-dimensional (1D and 2D) NMR (COSY, HSQC and HMBC), high-resolution electrospray ionization mass spectrometry (HRESIMS), quantum mechanical NMR (QM-NMR) methods, electronic circular dichroism (ECD) calculations, and comparison with literature data. All isolated substances were screened for antibacterial activities, and most exhibited moderate inhibitory effects against six pathogenic bacterial strains, with MIC values between 8 and 64 μg/mL. In addition, the effects of these compounds on LPS and IFN-γ stimulated RAW264.7 cells, focused on the release of NO and TNF-α, were also evaluated, but were inactive at 20 μM.

## 1. Introduction

The genus *Sarcophyton*, a highly prevalent group of soft corals, is widely recognized as a prolific source of biologically active secondary metabolites [1,2]. Natural products derived from species within this genus exhibit substantial chemical diversity, particularly enriched in terpenoid classes such as cembrane-type diterpenes, tetraterpenes, triterpenes, and ceramides, alongside steroids, sesquiterpenes, and fatty acids [3]. 

**Figure 1:**
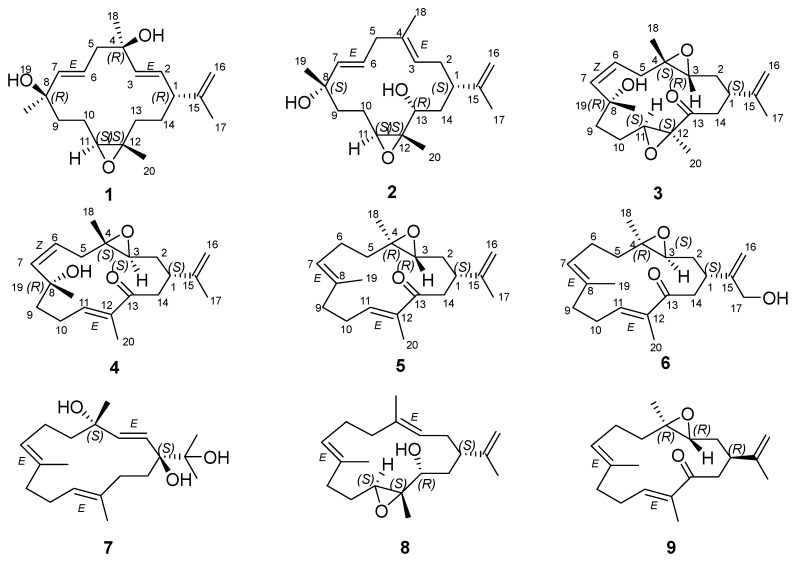
Chemical structures of compounds **1**–**9**.

Structurally, cembrane diterpenoids possess a 14-membered isoprenoid macrocyclic backbone characterized by an isopropyl group at C-1 and methyl groups located at C-4, C-8, and C-12 (Figure 1). The basic structure of this diterpene usually presents cyclic ether, lactone, or furan moieties around the macrocyclic ring. Due to extensive functional group variation, including lactone, epoxide, furan, ester, aldehyde, hydroxyl, and carboxyl moieties, as well as cyclization diversity, cembrane diterpenoids can be categorized into several subclasses, including simple cembranes, cembranolides, furanocembranoids, and biscembranoids [4]. The 14-membered carbocyclic nucleus of cembranolides is often bonded to a lactone ring with five, six, seven, or eight members [4]. Furanocembranoids feature a furan heterocycle and a 14-membered carbocyclic nucleus. Additionally, they include a butenolide moiety that includes C-10, C-12, and C-20. Biscembranoids have a tricyclic backbone of tetraterpenoids with 14–6–14 members. These compounds demonstrate a wide spectrum of biological activities, such as anti-cancer, neuroprotective, antimicrobial, antiviral, antidiabetic, antifouling, and anti-inflammatory effects [3,5,6].

In our continuous investigation of marine-derived bioactive compounds [7,8,9], we collected specimens of *Sarcophyton* sp. from the Xisha Islands. This study details the purification and structural characterization of six newly discovered cembranoids, designated as sarcophynoids D–I (**1**–**6**), along with three previously identified analogs (**7**–**9**) (Figure 1). Their antibacterial and anti-inflammatory properties were evaluated.

## 2. Results

The acetone extract of the title animals was partitioned between Et_2_O and H_2_O. The Et_2_O-soluble portion of the acetone extracts yielded the purified compounds **1**–**9** (Figure 1). The known compounds were identified as crassumol A (**7**) [10], 5-episinuleptolide (**8**) [11], and arbolide C (**9**) [12].

**Figure 2:**
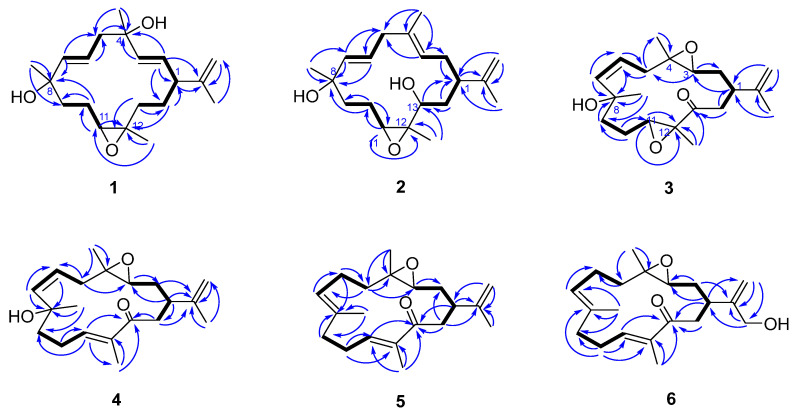
^1^H-^1^H COSY and key HMBC correlations of compounds **1**–**6**.

**Figure 3:**
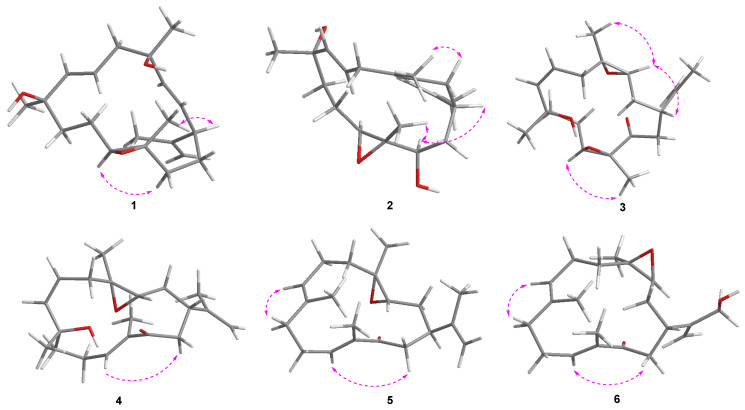
Key 1D NOEs of compounds **1**–**6**.

**Figure 4:**
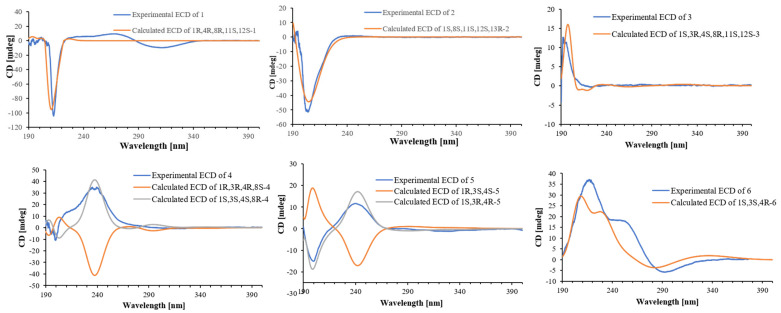
Calculated and experimental ECD data of compounds **1**–**6**.

Sarcophynoid D (**1**) was obtained as a colorless oil. Its molecular formula, C_20_H_32_O_3_, was established through HRESIMS, which exhibited an ion peak at *m*/*z* 303.2325 [M − H_2_O + H]^+^ (calculated 303.2324), indicating five degrees of unsaturation. Analysis of the ^13^C NMR and HSQC data suggested the presence of 20 carbon signals, including three double bonds, three nonprotonated sp^3^ carbons, two methine carbons, five methylene carbons, and four methyl groups. The planar framework of **1** was established by 2D NMR correlation analysis (Figure 2). The ^1^H–^1^H COSY correlations of H_2_-13/H_2_-14/H-1/H-2/H-3, H-1/H-2/H-3, H_2_-5/H-6/H-7, and H_2_-9/H_2_-10/H-11 revealed three partial structures. These data, in combination with the crucial HMBC from H-2, H-3, H_2_-5, and H_3_-15 to C-4, from H-6, H-7, H_2_-10, and H_3_-19 to C-8, from H-11, H_2_-13, H_2_-14, and H_3_-17 to C-12, and from H-1, H-2, H-3, H_2_-16, and H_3_-17 to C-15, established the skeleton of **1**. A comparison of the 1D and 2D NMR spectral data for compound **1** (Figure 2) with those of (1*S*,2*E*,4*R*,6*E*,8*R*,11*S*,12*S*)-11,12-epoxy-2,6-cembrane-4,8-diol, a naturally occurring cembrane derived from Australian *Sarcophyton* sp., revealed that they possess the same planar structure [13]. The difference was the existence of the Δ^15,16^ double bond of compound **1**. The larger coupling constants (*J_2,3_* = 15.7 Hz, and *J_6,7_* = 15.6 Hz) supported the *E* configurations of the Δ^2,3^ and Δ^6,7^ double bonds. As reported by Quinn’s work, the change in configuration at C-8 is clearly reflected by the ^13^C chemical shift of the Me-19 group (27.8 ppm in 8*S* and 30.9 ppm in 8*R*), which suggested the 8*R* configuration of compound **1**. The chemical shifts around the epoxide at C-11 and C-12 also suggested that this functional group should have the 11*S*,12*S* configuration. The 1D NOE correlation from H-13b (*δ*_H_ 1.01) to H-11, from H_3_-20 to H-1, together with the absence NOEs of H-11/H_3_-20, H-13b/H-1 and H-13b/H_3_-20, indicated that H-13b and H-11 were oriented toward the same face, relative to H-1 and H_3_-20 (Figure 3). Together with the quantum mechanical NMR (QM-NMR) approach [14,15], was applied to assign the relative configurations at C-1, C-4, C-8, C-11 and C-12. Four possible configurations were calculated: **1a** (1*R*,4*S*,8*R*,11*S*,12*S*) and **1b** (1*R*,4*R*,8*R*,11*S*,12*S*) (Figure S10). The computational and experimental data were correlated using the DP4+ procedure, and the relevant DP4+ probabilities were calculated [14,15]. According to Figure S10, configuration **1b** showed a dominating probability, indicating that the relative configuration of **1** is (1R*,4R*,8R*,11S*,12S*). Ultimately, the absolute configuration of **1** was determined as 1*R*,4*R*,8*R*,11*S*,12*S* through time-dependent density functional theory (TDDFT) ECD calculations [16,17] (Figure 4).

Sarcophynoid E (**2**) was isolated as a colorless oil and shared the same molecular formula as **1**. A comparison of the 1D NMR spectra of compounds **2** and **1** indicated that they possess similar structural groups, with the primary differences being the position of the double bonds, the presence of one more sp^3^ oxygenated methine carbon, and one less sp^3^ oxygenated quaternary carbon in **2**. The structure and assignments were further validated through 2D-NMR experiments. The *E* geometry of the Δ^3,4^ and Δ^6,7^ double bonds in compound **2** was corroborated by 1D NOE data and a larger coupling constant (*J_6,7_* = 15.5 Hz). In the NOE experiment (Figure 3), it was found NOE correlations between H-13 and H_3_-20, H-13 and H-1, and no NOE could be detected between H-11 and H_3_-20, and H-11 and H-13, suggesting that H-13 and H_3_-20, H-1 and H-13, were oriented toward the same face, as well as H-11 and H_3_-20, and H-11 and H-13, were oriented toward the opposite face. Since there is no clear relationship between the stereocluster C11-C12-C13 and the other stereoclusters, four possible configurations were calculated: **2a** (1*S*,8*S*,11*R*,12*R*,13*S*), **2b** (1*S*,8*R*,11*R*,12*R*,13*S*), **2c** (1*S*,8*S*,11*S*,12*S*,13*R*), and **2d** (1*S*,8*R*,11*S*,12*S*,13*R*). The relative configuration of **2** was confirmed to be 1S*,8S*,11S*,12S*,13R* by DP4^+^ calculations (Figure S21). The absolute configuration of compound **2** was established as 1*S*,8*S*,11*S*,12*S*,13*R* through TDDFT/ECD calculations.

Sarcophynoid F (**3**) has a molecular formula of C_20_H_30_O_4_ as determined by HRESIMS and NMR data, which indicates 6 degrees of unsaturation. The 1D NMR data (Table 1 and Table 2) and HSQC experiments revealed the presence of 20 carbon signals, including a ketone carbon, two disubstituted double bonds, three sp^3^ oxygenated quaternary carbons, three sp^3^ methine, five sp^3^ methylenes, and four methyl groups, indicating that compound **3** is also a cembranoid as compound **2**, with differences in the functional group at the position of C-3 and C-4, and the oxidation of hydroxyl group in **2** to ketone group in **3**. The proposed structure of **3** was supported by the comprehensive 1D and 2D NMR data. The *Z* geometry of the Δ^6,7^ double bonds in compound **3** was corroborated by a smaller coupling constant (*J_6,7_* = 6.3 Hz). The critical NOE correlations (Figure 3) of H-3/H-1 and H-3/H_3_-18 indicated H-1, H-3, and H_3_-18 are on the same side, and NOE correlations of H-11/H_3_-20 indicated H-11 and H_3_-20 are on the same side. The relative configuration of **3** was further confirmed to be 1S*,3R*,4S*,8R*,11S*,12S* by DP4^+^ calculations. The absolute configuration of **3** was established as 1*S*,3*R*,4*S*,8*R*,11*S*,12*S* by TDDFT/ECD calculations.

Sarcophynoid G (**4**) was also a colorless oil. Its molecular formula was established as C_20_H_30_O_3_ based on HRESIMS ion peak at *m*/*z* 319.2296 [M + H]^+^ (calculated for C_20_H_31_O_3_, 319.2273), indicating six degrees of unsaturation. The NMR data for compound **4** closely resembled that of compound **3**, suggesting that they are structural analogs. The main difference was that the functional group at C-11 and C-12 was a double bond (*δ*_C_/*δ*_H_ 144.4/6.72 and *δ*_C_ 136.5) in **4** rather than the epoxy ring in **3**. The proposed structure of **4** was supported by the comprehensive 1D and 2D NMR data. The *Z* and *E* geometry of the Δ^6,7^ and Δ^11,12^ double bonds in **4** was supported by a smaller coupling constant (*J_6,__7_* = 7.9 Hz), as well as the 1D NOE enhancement of H-11/H_2_-14, respectively. No NOE could be detected between H_3_-18 and H-3, suggesting that H-3 and H_3_-18 were oriented toward the opposite face. The relative configurations of compound **4** were indicated as 1R*,3R*,4R*,8S*, which were established by DP4^+^ calculations (Figure S40). Finally, the absolute configuration of **4** was established as 1*S*,3*S*,4*S*,8*R* by TDDFT/ECD calculations.

The molecular formula of compound **5** (sarcophynoid H) was determined to be C_20_H_30_O_2_ from its HRESIMS ion peak at *m*/*z* 303.2396 [M + H]^+^ (calculated for C_20_H_31_O_2_, 303.2324). The 1D and 2D NMR analysis indicated that **5** should possess the same planar structure as eunicenone Ⅳ, a cembrane-type diterpene isolated from the lipid extract of the Caribbean gorgonian *Eunicea mammosa* [18]. The configurations of the Δ^7,8^ and Δ^11,12^ double bonds were assigned as *E*, which were supported by the 1D NOE enhancements of H-7/H_2_-9 and H-11/H_2_-14. The absence of NOEs between H_3_-18 and H-3 suggested that H-3 and H_3_-18 were oriented toward the opposite face. Additionally, the relative configuration of compound **5** was confirmed as 1R*,3S*,4S* by DP4^+^ calculations. The absolute configuration of compound **5** was established as 1S*,3R*,4R* by TDDFT/ECD calculations (Figure 4).

Sarcophynoid I (**6**) was also isolated as a colorless oil. Its molecular formula was determined to be C_20_H_30_O_3_ via HRESIMS (*m*/*z* 319.2296 [M + H]^+^, calculated 319.2274), indicating six degrees of unsaturation. The NMR spectra of **6** (Table 1 and Table 2) showed great significance to those of **5**, except that a primary alcohol (17-CH_2_OH) in **6** rather than the methyl (17-CH_3_) in **5**. The configurations of the Δ^7,8^ and Δ^11,12^ double bonds were also assigned as *E*, based on the same method as for **5**. The relative configuration of **6** was confirmed as 1S*,3S*,4R* by DP4^+^ calculations. The absolute configuration of **6** was established as 1*S*,3*S*,4*R* by TDDFT/ECD calculations.

Compounds **1**–**9** were evaluated for the inhibition of NO and TNF-α production in lipopolysaccharide (LPS) and interferon-gamma stimulated RAW 264.7 macrophages. However, all compounds were inactive at 20 μM. Furthermore, all compounds were evaluated for antibacterial activity against six pathogenic bacterial strains (*Pseudomonas aeruginosa*, *Bacillus subtilis*, *Staphylococcus aureus*, *Enterococcus faecalis*, *Staphylococcus saprophyticus,* and *Staphylococcus white*). The results showed that most of the compounds displayed moderate inhibitory activity (MIC 8–64 μg/mL) (Table 3). 

## 3. Materials and Methods

### 3.1. General Chemical Experimental Procedures

NMR spectra were recorded on a Bruker AVANCE NEO 600 spectrometer (BrukerBiospin AG, Fällanden, Germany). ^1^H chemical shifts were referenced to the residual CDCl_3_ (7.26 ppm) and ^13^C chemical shifts were referenced to the CDCl_3_ (77.2 ppm) solvent peaks. High-resolution electrospray ionization mass spectra (HRESIMS) were performed on an ultra-high-performance liquid chromatograph (UPLC) and TIMS-QTOF high-resolution mass spectrometry (Waters, Milford, MA, USA). The purification was performed by reversed-phase high-performance liquid chromatography using a Shimadzu LC-20AT system (Shimadzu Corporation, Tokyo, Japan). The solvents used for HPLC were all Fisher HPLC grade. A Cosmosil C_18_-MS-II column (250 mm × 10.0 mm, id, 5 μm, Cosmosil, Nakalai Tesque Co. Ltd., Kyoto, Japan) was used for the preparative HPLC separation. Column chromatography was performed using silica gel (300–400 mesh, Qingdao Ocean Chemical Co. Ltd., Qingdao, China) and C_18_ reversed-phase silica gel (75 µm, Nakalai Tesque Co. Ltd., Kyoto, Japan). 

### 3.2. Animal Material

Soft coral *Sarcophyton* sp. was sampled off the coast of Xisha Islands, South China Sea, wet weight was 8.24 kg, and was frozen immediately after collection. The specimens (XSSC20190803) were deposited at the Li Dak Sum Yip Yio Chin Kenneth Li Marine Biopharmaceutical Research Center, Health Science Center, Ningbo University, China.

### 3.3. Extraction and Isolation

The soft coral samples were vacuum freeze-dried with a freeze-dryer, crushed in a pulverizer, and fully soaked in acetone at room temperature for 2 days each time, followed by ultrasonic extraction for an hour, repeated soaking and ultrasonic extraction for 4–5 times. The extract was filtered to remove the sample residue, and the extract was concentrated under reduced pressure. The extract was partitioned three times with Et_2_O and water (1:1, *v*/*v*), and the Et_2_O layer extract was concentrated under reduced pressure to obtain 67 g brown residue.

The 67 g of the extract was separated by gradient elution on a normal-phase silica gel column, yielding 11 fractions (FrA–FrK). Fr.E (4.7 g) was eluted with MeOH/H_2_O (60:40 to 100:0, *v*/*v*) on reversed-phase column chromatography to obtain six subfractions (Fr.E.1–Fr.E.6). Purification of Fr.E.3 by semi-preparative HPLC (MeCN/H_2_O, 55:45, 2 mL/min) gave compounds **1** (3.8 mg, t*_R_* = 62 min) and **2** (5.8 mg, t*_R_* = 67 min). Separation of Fr.F (2.3 g) on a reversed-phase column with MeOH/H_2_O (55:45~100:0, *v*/*v*) afforded five subfractions (Fr.F.1~Fr.F.5). Fr.F.3 was purified by semipreparative reversed-phase HPLC (MeCN/H_2_O, 53: 47, 2 mL/min) to afford compounds **3** (3.5 mg, t*_R_* = 54 min), **4** (2.8 mg, t*_R_* = 57 min) and **8** (3.8 mg, t*_R_* = 63 min). Fr.F.4 was purified by semipreparative reversed-phase HPLC (MeCN/H_2_O, 55: 45, 2 mL/min) to afford compound **5** (2.8 mg, t*_R_* = 48 min). Separation of Fr.I (2.5 g) on a reversed-phase column with MeOH/H_2_O (50:50~100:0, *v*/*v*) afforded seven subfractions (Fr.I.1~Fr.J.7). Fr.I.3 was purified by semipreparative reversed-phase HPLC (MeCN/H_2_O, 42:58, 2 mL/min) to afford compounds **6** (6.7 mg, t*_R_* = 63 min) and **7** (5.2 mg, t*_R_* = 67 min). Fr.I.4 was purified by semipreparative reversed-phase HPLC (MeCN/H_2_O, 44:56, 2 mL/min) to afford compound **8** (5.9 mg, t*_R_* = 48 min) and **9** (8.3 mg, t*_R_* = 52 min). 

Sarcophynoid D (**1**): colorless oil; {[α${]}_{D}^{25}$ −42.5 (c 0.5, MeOH)}; UV (MeOH): 205 (3.72); IR (KBr) *ν*_max_ 3362, 2973, 2928, 2858, 1635, 1445, 1394, 1094, 1043, 1016 cm^−1^; ^1^H and ^13^C NMR data, Table 1 and Table 2; HRESIMS *m*/*z* 303.2325 [M − H_2_O + H]^+^ (calcd for C_20_H_31_O_2_, 303.2324).

Sarcophynoid E (**2**): colorless oil; {[α${]}_{D}^{25}$ −41.0 (c 0.5, MeOH)}; UV (MeOH): 204 (3.31); IR (KBr) *ν*_max_ 3395, 2957, 2832, 1673, 1463, 1375, 1094, 1028 cm^−1^; ^1^H and ^13^C NMR data, Table 1 and Table 2; HRESIMS *m*/*z* 303.2325 [M − H_2_O + H]^+^ (calcd for C_20_H_31_O_2_, 303.2324).

Sarcophynoid F (**3**): colorless oil; {[α${]}_{D}^{25}$ +13.5 (c 0.5, MeOH)}; UV (MeOH): 203 (2.62); IR (KBr) *ν*_max_ 3478, 2926, 2775, 1729, 1655, 1417, 1124, 1095 cm^−1^; ^1^H and ^13^C NMR data, Table 1 and Table 2; HRESIMS *m*/*z* 335.2220 [M + H]^+^ (calcd for C_20_H_31_O_4_, 335.2222).

Sarcophynoid G (**4**): colorless oil; {[α${]}_{D}^{25}$ +36.0 (c 0.5, MeOH)}; UV (MeOH): 205 (3.25); IR (KBr) *ν*_max_ 3358, 2965, 1710, 1668, 1448, 1086, 1015 cm^−1^; ^1^H and ^13^C NMR data, Table 1 and Table 2; HRESIMS *m*/*z* 319.2296 [M + H]^+^ (calcd for C_20_H_31_O_3_, 319.2273).

Sarcophynoid H (**5**): colorless oil; {[α${]}_{D}^{25}$ −20.5 (c 0.5, MeOH)}; UV (MeOH): 207 (3.23); IR (KBr) *ν*_max_ 2957, 2843, 1728, 1659, 1443, 1396, 1228, 1132, 1081 cm^−1^; ^1^H and ^13^C NMR data, Table 1 and Table 2; HRESIMS *m*/*z* 303.2396 [M + H]^+^ (calcd for C_20_H_31_O_3_, 303.2324).

Sarcophynoid I (**6**): colorless oil; {[α${]}_{D}^{25}$ +21.4 (c 0.5, MeOH)}; UV (MeOH): 205 (3.80); IR (KBr) *ν*_max_ 3370, 2926, 1760, 1628, 1460, 1073 cm^−1^; ^1^H and ^13^C NMR data, Table 1 and Table 2; HRESIMS *m*/*z* 319.2296 [M + H]^+^ (calcd for C_20_H_31_O_3_, 319.2274).

### 3.4. Computational Section

The NMR calculations method and the simulation of NMR spectra were referenced with the procedure described in the literature [14,15]. The ECD calculations method followed the protocol described in the literature [16,17].

### 3.5. Antibacterial Assays

Antibacterial activities of all isolated compounds were evaluated using standardized protocols [19]. The assay included six bacterial strains: *Pseudomonas aeruginosa* [CMCC (B) 10104], *Bacillus subtilis* [CMCC (B) 63501], *Staphylococcus aureus* [CMCC (B) 26003], *Enterococcus faecalis* [ATCC 29212], *Staphylococcus saprophyticus* [ATCC 49453], and *Staphylococcus hominis* [ATCC 8032], with penicillin G employed as the positive control. Compounds **1**–**9** were dissolved in DMSO and tested across serial concentrations of 64, 32, 16, 8, 4, 2, 1, and 0.5 μg/mL.

In brief, the bacterial strains were cultured in Mueller–Hinton (MH) medium for 24 h at 28 °C with shaking at 180 rpm, followed by dilution in sterile MH medium to a turbidity equivalent to the 0.5 McFarland standard. Subsequently, 100 μL of each bacterial suspension was combined with 100 μL of MH medium containing 0.002% 2,3,5-triphenyltetrazolium chloride and the corresponding test or control compound. After incubation, bacterial growth inhibition was quantified by optical measurements.

## 4. Conclusions

In conclusion, the phytochemical investigation of *Sarcophyton* sp., a soft coral collected from the South China Sea, resulted in the discovery of six previously undescribed cembranoids, named sarcophynoids D–I (**1**–**6**), together with three known analogues. Structural determination of these metabolites was achieved through high-resolution electrospray ionization mass spectrometry (HRESIMS), nuclear magnetic resonance (NMR) spectroscopy, quantum mechanical NMR (QM-NMR) analyses, and electronic circular dichroism (ECD) calculations, in combination with comparison to reported data. Most of the isolated cembranoids demonstrated moderate antibacterial effects, displaying minimum inhibitory concentrations (MICs) between 8 and 64 μg/mL. The discovery of these new metabolites from marine soft corals highlights the remarkable chemical diversity of *Sarcophyton* species and emphasizes the importance of investigating their antimicrobial potential for future public health applications.

## Figures and Tables

**Table 1:** ^1^H NMR data of compounds **1**–**6** at 600 MHz in CDCl_3_.

No.	1	2	3	4	5	6
1	2.50, m	1.87, m	2.70, m	2.72, ov	2.68, m	2.78, m
2a	5.31, dd (15.7,9.3)	2.04, m	1.79, ov	1.94, m	1.77, m	1.78, m
2b		1.97, m	1.63, m	1.43, ddd (14.4, 9.5, 3.6)	1.37, ov	1.54, m
3	5.72, d (15.7)	5.05, tdd (5.8, 2.9, 1.4)	2.61, dd (7.4, 4.8)	2.72, ov	2.70, ov	2.79, dd (7.0, 5.0)
5a	2.54, dt (14.4, 2.6)	2.73, m	2.56, dd (4.5, 1.3)	2.61, m	2.03, ddd (13.9, 5.7, 3.6)	2.11, m
5b	2.14, dd (14.4, 10.7)	2.67, m	2.00, dd (14.5, 5.8)	1.84, m	1.41, ov	1.22, m
6a	5.94, ddd (15.6, 10.8, 2.9)	5.88, ddd (15.5, 9.4, 5.3)	5.44, ov	5.60, ov	2.14, m	2.19, m
6b					2.14, m	2.13, m
7	5.58, dd (15.6, 2.1)	5.54, d (15.5)	5.45, ov	5.60, ov	5.11, br.t (6.6)	5.14, br.t (9.2)
9a	1.79, m	1.88, m	1.79, ov	1.92, m	2.32, m	2.20, m
9b		1.73, m	1.61, m	1.84, ov	2.21, m	2.20, m
10a	1.96, ov	1.95, m	1.40, m	2.48, m	2.44, m	2.48, m
10b	1.22, m	1.41, m	1.35, m	2.33, m	2.44, m	2.40, m
11	2.99, dd (11.1, 1.9)	2.96, dd (9.4, 2.7)	2.95, dd (7.8,5.3)	6.72, ddd (8.0, 6.5, 1.5)	6.58, td (7.4, 1.4)	6.66, t (6.6)
13a	1.96, ov	3.59, dt (10.5, 4.0)				
13b	1.01, m					
14a	1.63, m	1.54, ddd (14.1, 11.1, 3.7)	2.80, dd (6.8, 0.8)	3.16, dd (14.7,8.8)	2.82, dd (12.7, 7.6)	3.47, dd (12.8, 8.8)
14b	1.25, m	1.36, m		2.42, dd (14.7, 6.0)	2.55, dd (12.7, 7.1)	2.33, dd (12.8, 6.5)
16a	4.70, d (0.9)	4.79, br.s	4.88, br.s	4.79, t (1.5)	4.76, br.s	5.14, br.s
16b	4.69, t (1.5)	4.76, br.s	4.77, br.s	4.69, br.s	4.71, br.s	4.88, br.s
17	1.69, s	1.77, s	1.76, s	1.77, s	1.73, s	4.10, s
18	1.38, s	1.64, s	1.28, s	1.17, s	1.16, s	1.77, s
19	1.30, s	1.35, s	1.28, s	1.34, s	1.64, s	1.62, s
20	1.20, s	1.33, s	1.42, s	1.74, s	1.71, s	1.23, s
13-OH		2.12, br.d (5.8)				

**Table 2:** ^13^C NMR data of compounds **1**–**6** at 150 MHz in CDCl_3_.

No.	1	2	3	4	5	6
1	48.7, CH	44.7, CH	36.9, CH	41.1, CH	44.4, CH	40.4, CH
2	129.9, CH	33.0, CH_2_	31.9, CH_2_	30.6, CH_2_	31.6, CH_2_	31.7, CH_2_
3	137.9, CH	123.7, CH	58.7, CH	60.9, CH	63.0, CH	61.1, CH
4	72.3, C	136.8, C	60.6, C	60.7, C	60.5, C	60.3, C
5	46.9, CH_2_	41.5, CH_2_	41.6, CH_2_	42.1, CH_2_	37.3, CH_2_	38.7, CH_2_
6	122.2, CH	125.7, CH	123.8, CH	123.6, CH	23.6, CH_2_	24.0, CH_2_
7	139.4, CH	137.6, CH	139.9, CH	139.8, CH	126.5, CH	125.5, CH
8	73.4, C	73.2, C	72.8, C	72.8, C	134.1, C	134.3, C
9	38.0, CH_2_	38.1, CH_2_	38.6, CH_2_	41.2, CH_2_	38.7, CH_2_	38.2, CH_2_
10	22.4, CH_2_	22.1, CH_2_	25.2, CH_2_	24.8, CH_2_	25.8, CH_2_	25.6, CH_2_
11	65.6, CH	62.4, CH	64.2, CH	144.4, CH	143.5, CH	144.5, CH
12	61.1, C	63.6, C	66.7, C	136.5, C	136.4, C	138.3, C
13	36.1, CH_2_	70.6, CH	208.9, C	200.8, C	201.9, C	202.7, C
14	29.3, CH_2_	34.5, CH_2_	41.8, CH_2_	39.6, CH_2_	42.7, CH_2_	39.3, CH_2_
15	148.9, C	148.0, C	147.7, C	147.5, C	147.1, C	150.8, C
16	109.7, CH_2_	111.2, CH_2_	110.3, CH_2_	110.9, CH_2_	111.7, CH_2_	112.6, CH_2_
17	21.0, CH_3_	19.7, CH_3_	21.6, CH_3_	20.6, CH_3_	18.9, CH_3_	65.4, CH_2_
18	27.4, CH_3_	17.3, CH_3_	17.7, CH_3_	16.7, CH_3_	17.0, CH_3_	11.4, CH_3_
19	31.1, CH_3_	31.2, CH_3_	27.9, CH_3_	28.9, CH_3_	15.6, CH_3_	15.6, CH_3_
20	15.5, CH_3_	18.0, CH_3_	19.1, CH_3_	11.4, CH_3_	11.5, CH_3_	16.6, CH_3_

**Table 3:** Inhibitory effects of **1**–**9** on six kinds of pathogenic bacteria.

No.	MIC (μg/mL)					
	*P. aeruginosa*	*B. subtilis*	*S. aureus*	*E. faecalis*	*S. saparophytics*	*S. white*
**1**	>64	64	64	32	32	64
**2**	>64	>64	64	>64	8	64
**3**	>64	>64	64	>64	>64	8
**4**	>64	64	>64	>64	16	>64
**5**	32	>64	64	64	32	64
**6**	>64	64	64	>64	64	64
**7**	32	64	64	64	64	64
**8**	16	16	32	32	>64	>64
**9**	64	>64	>64	>64	64	64
Penicillin ^a^	≤0.5	≤0.5	≤0.5	≤0.5	≤0.5	≤0.5

^a^ Penicillin was used as a positive control.

## Data Availability

The data presented in this study are available in the Supplementary Materials file associated with this article.

## Supplementary Materials

The following supporting information can be downloaded at: https://www.mdpi.com/article/10.3390/md23110422/s1, Figures S1–S63: HRESIMS, 1D and 2D NMR spectra, and DP4+ data of all new compounds; Figures S64–S69: ^1^H and ^13^C NMR data for the known compounds.
