# Supplementary material for: Cembrane-Based Diterpenoids Isolated from the Soft Coral Sarcophyton sp
Source: Mar Drugs. 2025 Oct 30;23(11):422. doi: 10.3390/md23110422 (PMC12653649; doi:10.3390/md23110422)
Supplement: Supplementary file 1 [file marinedrugs-23-00422-s001.zip › marinedrugs-3835902-supplementary.pdf]

Supporting Information

Figure S1 HRESIMS spectrum of compound **1**....3
Figure S2 ^{1}H NMR spectrum of compound **1** in $CDCl_3$, 600 MHz....3
Figure S3 ^{13}C NMR spectrum of compound **1** in $CDCl_3$, 150 MHz....4
Figure S4 DEPT 135 spectrum of **1** in $CDCl_3$, 150 MHz....4
Figure S5 ^{1}H-^{1}H COSY spectrum of **1** in $CDCl_3$, 600 MHz....5
Figure S6 HSQC spectrum of **1** in $CDCl_3$, 150 MHz....5
Figure S7 HMBC spectrum of **1** in $CDCl_3$, 150 MHz....6
Figure S8 1D NOE spectrum of **1** in $CDCl_3$, 600 MHz (H_3-20)....6
Figure S9 1D NOE spectrum of **1** in $CDCl_3$, 600 MHz (H-11)....7
Figure S10 1D NOE spectrum of **1** in $CDCl_3$, 600 MHz (H-13b)....7
Figure S11 Detailed DP4+ probability (calculated at PCM/mPW1PW91/6-31+G (d, p) level) for compound **1**. Isomer **1** is 1*R*,4*S*,8*R*,11*S*,12*S*, isomer **2** is 1*R*,4*R*,8*R*,11*S*,12*S*....8
Figure S12 HRESIMS spectrum of compound **2**....9
Figure S13 ^{1}H NMR spectrum of compound **2** in $CDCl_3$, 600 MHz....9
Figure S14 ^{13}C NMR spectrum of compound **2** in $CDCl_3$, 150 MHz....10
Figure S15 DEPT 135 spectrum of **2** in $CDCl_3$, 150 MHz....10
Figure S16 ^{1}H-^{1}H COSY spectrum of **2** in $CDCl_3$, 600 MHz....11
Figure S17 HSQC spectrum of **2** in $CDCl_3$, 150 MHz....11
Figure S18 HMBC spectrum of **2** in $CDCl_3$, 150 MHz....12
Figure S19 1D NOE spectrum of **2** in $CDCl_3$, 600 MHz (H_3-18)....12
Figure S20 1D NOE spectrum of **2** in $CDCl_3$, 600 MHz (H-11)....13
Figure S21 1D NOE spectrum of **2** in $CDCl_3$, 600 MHz (H-13)....13
Figure S22 Detailed DP4+ probability (calculated at PCM/mPW1PW91/6-31+G (d, p) level) for compound **2**. Isomer **1** is *1*S,*8*S,*11*R,*12R,13*S, isomer **2** is *1*S,*8R,11R,12R,13S*, isomer **3** is *1S,8S,11S,12S,13R*, isomer **4** is 1*S*,8*R*,11*S*,12*S*,13*R*....14
Figure S23 HRESIMS spectrum of compound **3**....15
Figure S24 ^{1}H NMR spectrum of compound **3** in $CDCl_3$, 600 MHz....15
Figure S25 ^{13}C NMR spectrum of compound **3** in $CDCl_3$, 150 MHz....16
Figure S26 DEPT 135 spectrum of **3** in $CDCl_3$, 150 MHz....16
Figure S27 ^{1}H-^{1}H COSY spectrum of **3** in $CDCl_3$, 600 MHz....17
Figure S28 HSQC spectrum of **3** in $CDCl_3$, 150 MHz....17
Figure S29 HMBC spectrum of **3** in $CDCl_3$, 150 MHz....18
Figure S30 1D NOE spectrum of **3** in $CDCl_3$, 600 MHz (H-3)....18
Figure S31 1D NOE spectrum of **3** in $CDCl_3$, 600 MHz (H-11)....19
Figure S32 Detailed DP4+ probability (calculated at PCM/mPW1PW91/6-31+G (d, p) level) for compound **3**. Isomer **1** is 1*S*,3*R*,4*S*,8*R*,11*R*,12*R*, isomer **2** is 1*S*,3*R*,4*S*,8*R*,11*S*,12*S*, isomer **3** is 1*S*,3*R*,4*S*,8*S*,11*R*,12*R,* isomer **4** is 1*S*,3*R*,4*S*,8*S*,11*S*,12*S*....20
Figure S33 HRESIMS spectrum of compound **4**....20
Figure S34 ^{1}H NMR spectrum of compound **4** in $CDCl_3$, 600 MHz....21
Figure S35 ^{13}C NMR spectrum of compound **4** in $CDCl_3$, 150 MHz....21
Figure S36 DEPT 135 spectrum of **4** in $CDCl_3$, 150 MHz....22

Figure S37 ^{1}H-^{1}H COSY spectrum of **4** in $CDCl_3$, 600 MHz ... 22
Figure S38 HSQC spectrum of **4** in $CDCl_3$, 150 MHz ... 23
Figure S39 HMBC spectrum of **4** in $CDCl_3$, 150 MHz ... 23
Figure S40 1D NOE spectrum of **4** in $CDCl_3$, 600 MHz (H_3-18) ... 24
Figure S41 Detailed DP4+ probability (calculated at PCM/mPW1PW91/6-31+G (d, p) level) for compound **4**. Isomer **1** is 1*S*,3*S*,4*S*,8*S*, isomer **2** is 1*S*,3*R*,4*R*,8*S*, isomer **3** is 1*R*,3*S*,4*S*,8*S*, isomer **4** is 1*R*,3*R*,4*R*,8*S* ... 25
Figure S42 HRESIMS spectrum of compound **5** ... 25
Figure S43 ^{1}H NMR spectrum of compound **5** in $CDCl_3$, 600 MHz ... 26
Figure S44 ^{13}C NMR spectrum of compound **5** in $CDCl_3$, 150 MHz ... 26
Figure S45 DEPT 135 spectrum of **5** in $CDCl_3$, 150 MHz ... 27
Figure S46 ^{1}H-^{1}H COSY spectrum of **5** in $CDCl_3$, 600 MHz ... 27
Figure S47 HSQC spectrum of **5** in $CDCl_3$, 150 MHz ... 28
Figure S48 HMBC spectrum of **5** in $CDCl_3$, 150 MHz ... 28
Figure S49 1D NOE spectrum of **5** in $CDCl_3$, 600 MHz (H-3) ... 29
Figure S50 1D NOE spectrum of **5** in $CDCl_3$, 600 MHz (H_3-18) ... 29
Figure S51 1D NOE spectrum of **5** in $CDCl_3$, 600 MHz (H-7) ... 30
Figure S52 1D NOE spectrum of **5** in $CDCl_3$, 600 MHz (H-11) ... 30
Figure S53 Detailed DP4$^+$ probability (calculated at PCM/mPW1PW91/6-31+G (d, p) level) for compound **5**. Isomer **1** is 1*R*, 3*S*,4*S*, isomer **2** is 1*R*, 3*R*,4*R* ... 31
Figure S54 HRESIMS spectrum of compound **6** ... 32
Figure S55 ^{1}H NMR spectrum of compound **6** in $CDCl_3$, 600 MHz ... 32
Figure S56 ^{13}C NMR spectrum of compound **6** in $CDCl_3$, 150 MHz ... 33
Figure S57 DEPT 135 spectrum of **6** in $CDCl_3$, 150 MHz ... 33
Figure S58 ^{1}H-^{1}H COSY spectrum of **6** in $CDCl_3$, 600 MHz ... 34
Figure S59 HSQC spectrum of **6** in $CDCl_3$, 150 MHz ... 34
Figure S60 HMBC spectrum of **6** in $CDCl_3$, 150 MHz ... 35
Figure S61 1D NOE spectrum of **6** in $CDCl_3$, 600 MHz (H-11) ... 35
Figure S62 1D NOE spectrum of **6** in $CDCl_3$, 600 MHz (H-7) ... 36
Figure S63 Detailed DP4+ probability (calculated at PCM/mPW1PW91/6-31+G (d, p) level) for compound **6**. Isomer **1** is 1*S*,3*S*,4*S*, isomer **2** is 1*S*,3*R*,4*R*, isomer **3** is 1*S*,3*R*,4*S*, isomer **4** is 1*S*,3*S*,4*R* ... 37
Figure S64 ^{1}H NMR spectrum of compound **7** in $CDCl_3$, 600 MHz ... 37
Figure S65 ^{13}C NMR spectrum of compound **7** in $CDCl_3$, 150 MHz ... 38
Figure S66 ^{1}H NMR spectrum of compound **8** in $CDCl_3$, 600 MHz ... 38
Figure S67 ^{13}C NMR spectrum of compound **8** in $CDCl_3$, 150 MHz ... 39
Figure S68 ^{1}H NMR spectrum of compound **9** in $CDCl_3$, 600 MHz ... 39
Figure S69 ^{13}C NMR spectrum of compound **9** in $CDCl_3$, 150 MHz ... 40

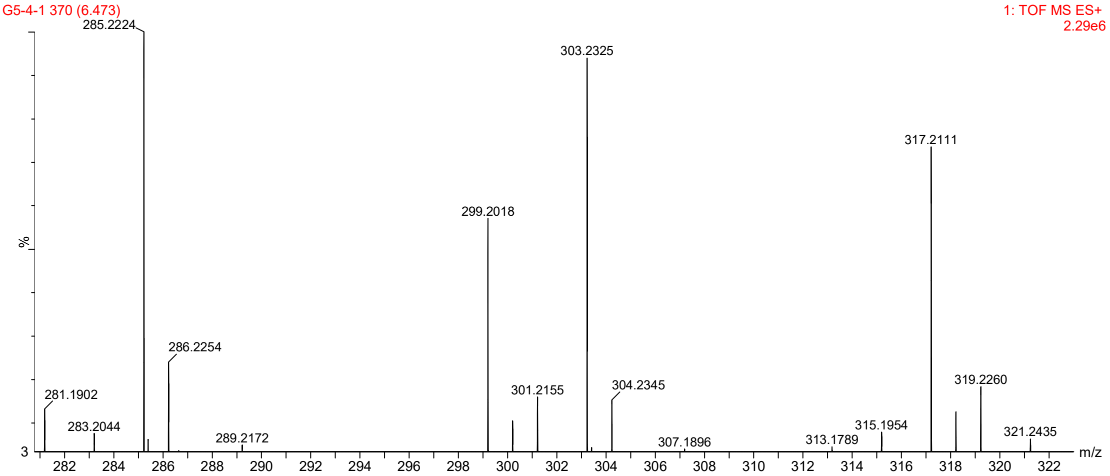

Figure S1 HRESIMS spectrum of compound **1**

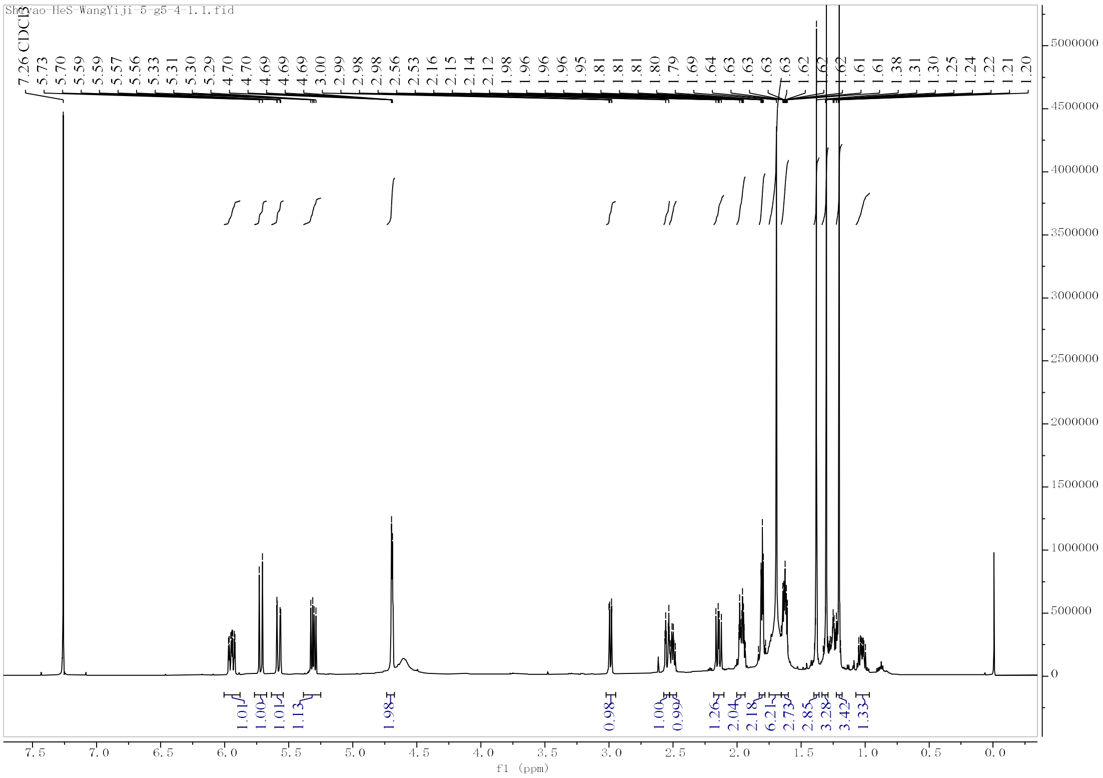

Figure S2 ^{1}H NMR spectrum of compound **1** in $CDCl_3$, 600 MHz

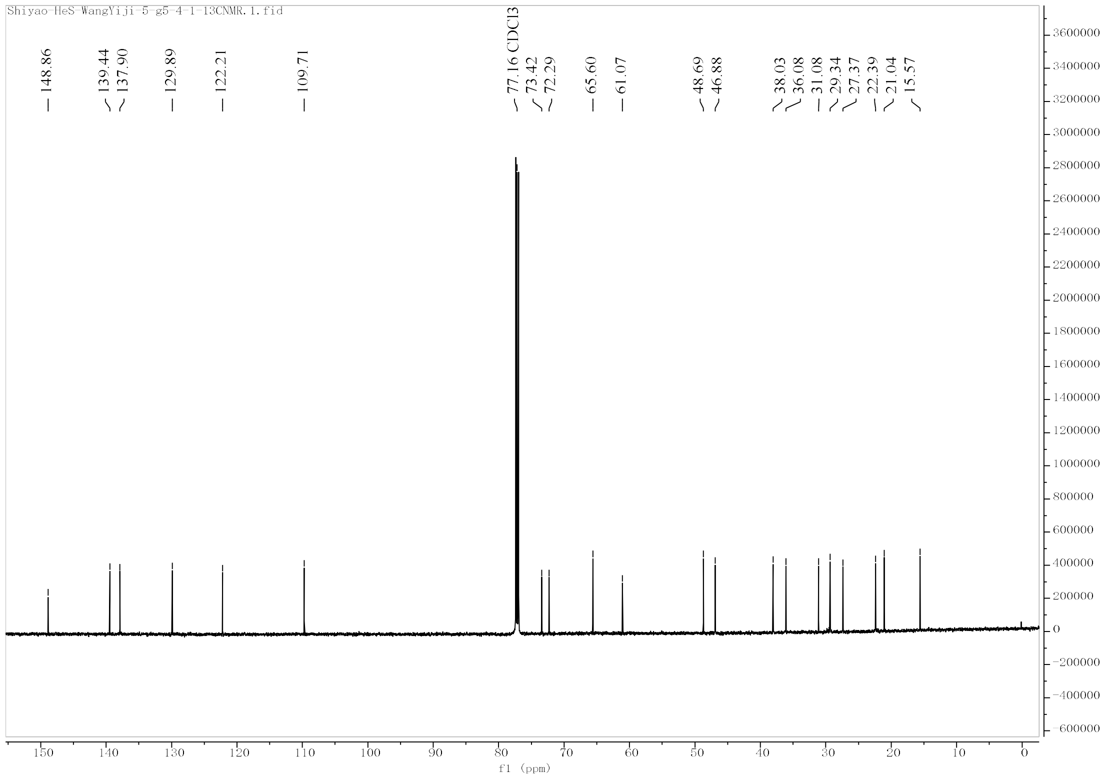

Figure S3 ^{13}C NMR spectrum of compound **1** in $CDCl_3$, 150 MHz

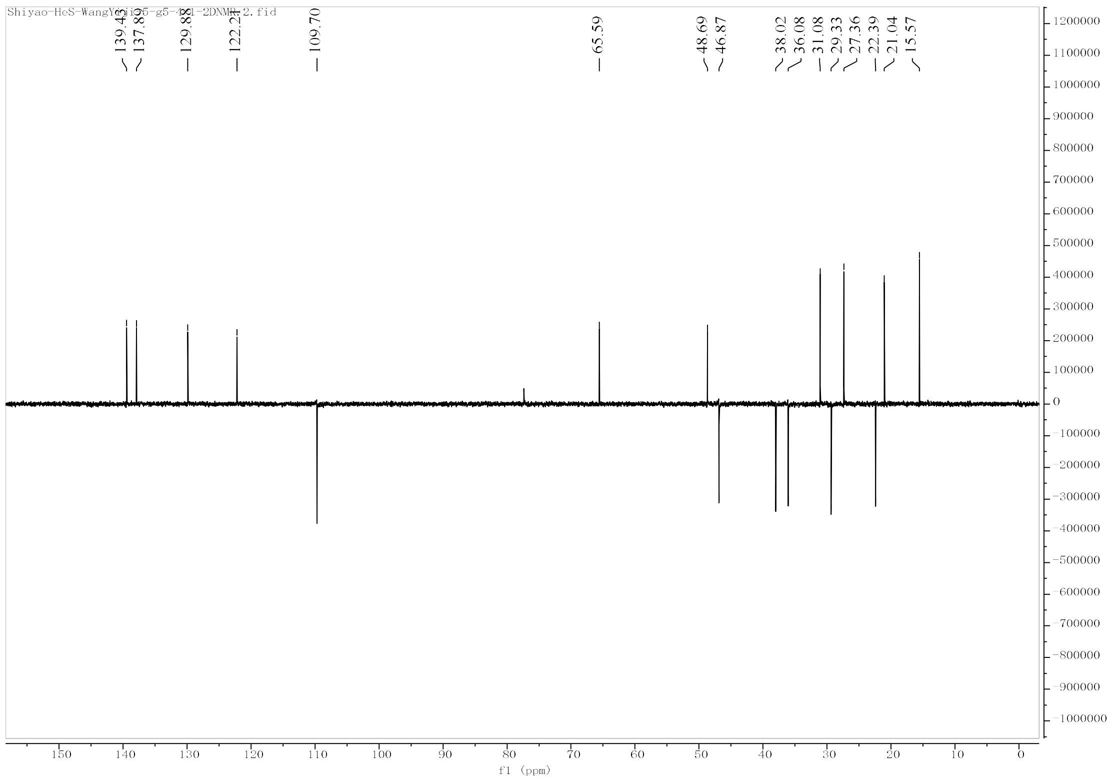

Figure S4 DEPT 135 spectrum of **1** in $CDCl_3$, 150 MHz

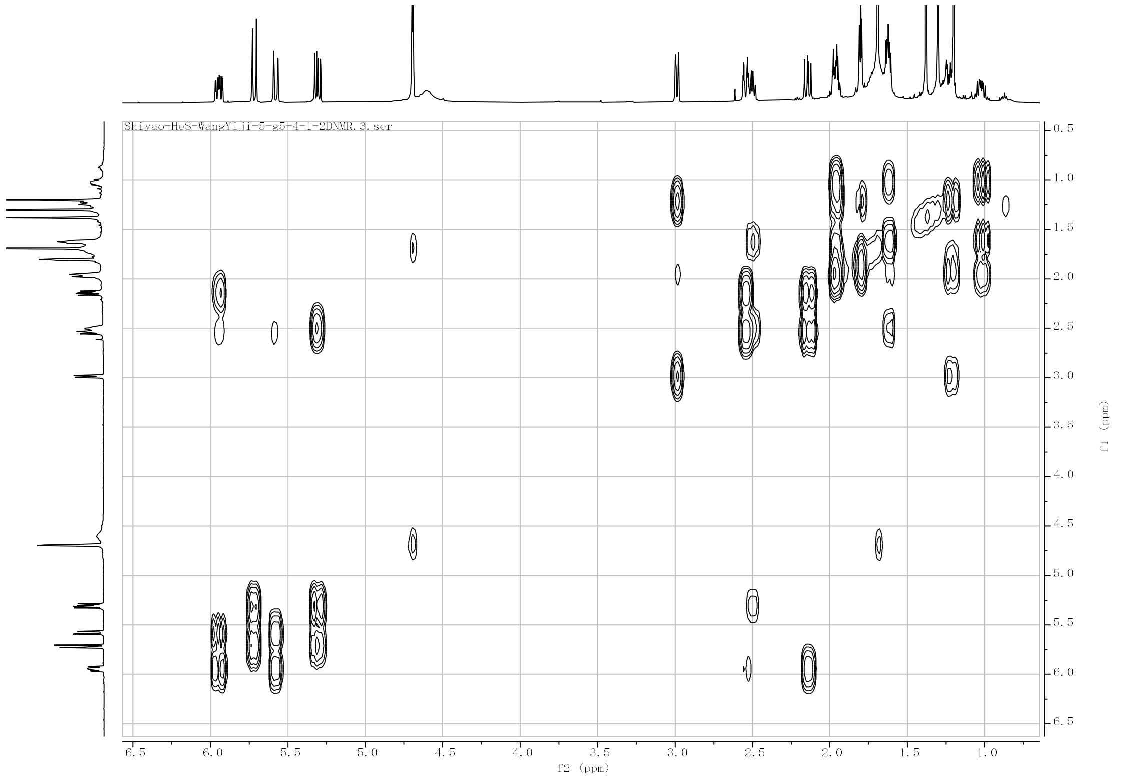

Figure S5 ^{1}H-^{1}H COSY spectrum of **1** in $CDCl_3$, 600 MHz

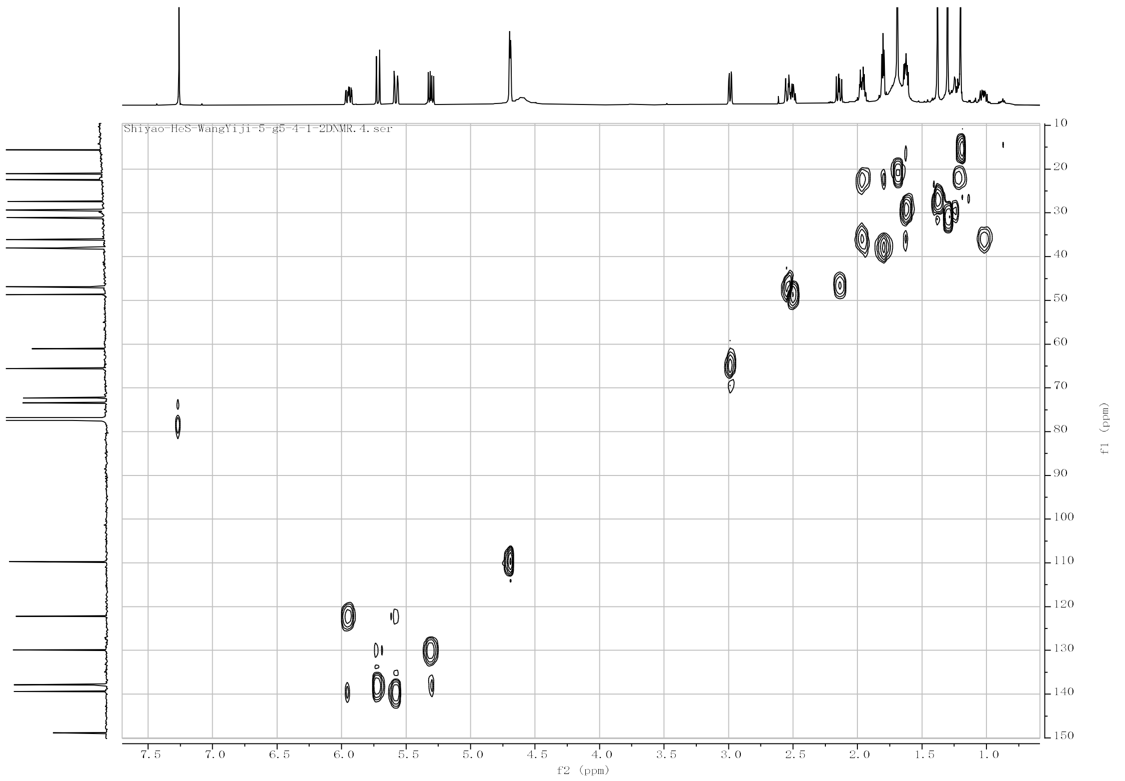

Figure S6 HSQC spectrum of **1** in $CDCl_3$, 150 MHz

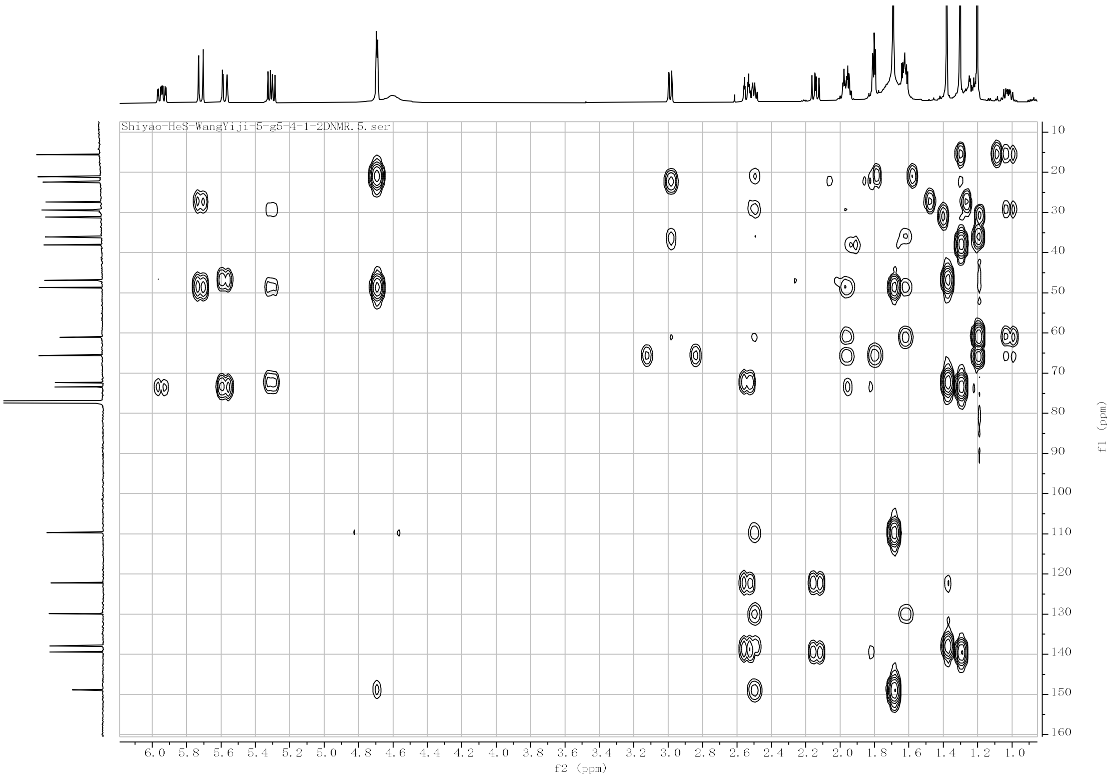

Figure S7 HMBC spectrum of **1** in $CDCl_3$, 150 MHz

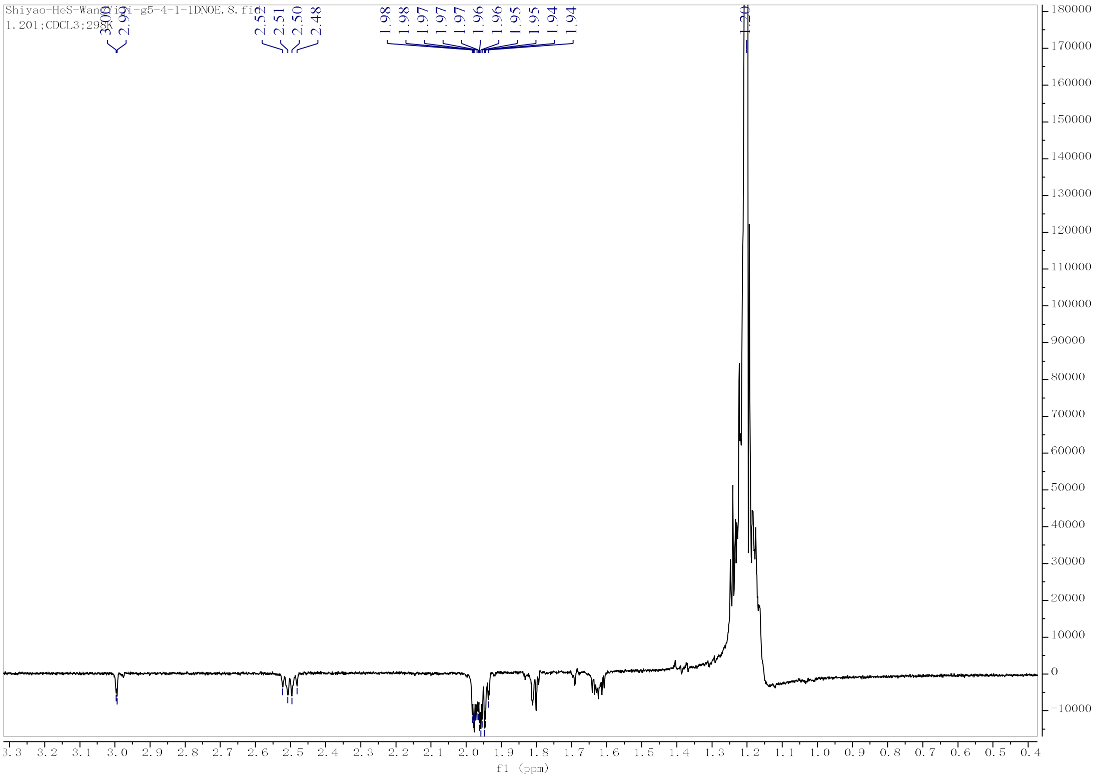

Figure S8 1D NOE spectrum of **1** in $CDCl_3$, 600 MHz (H_3-20)

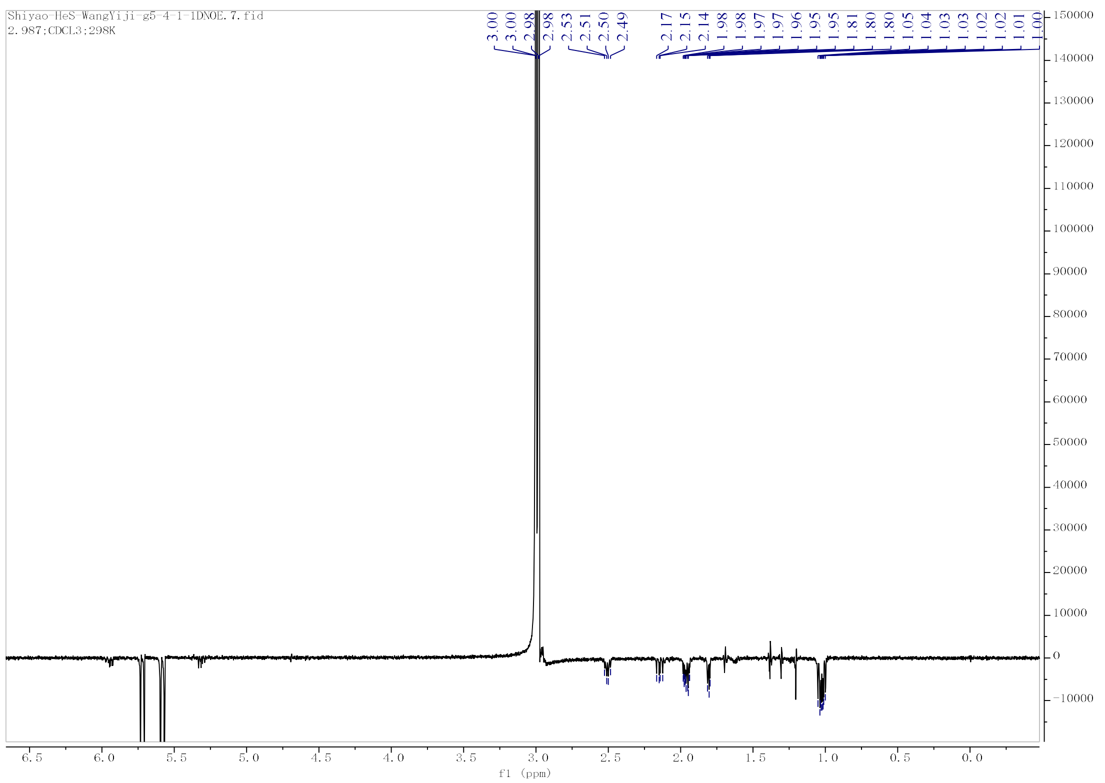

Figure S9 1D NOE spectrum of **1** in $CDCl_3$, 600 MHz (H-11)

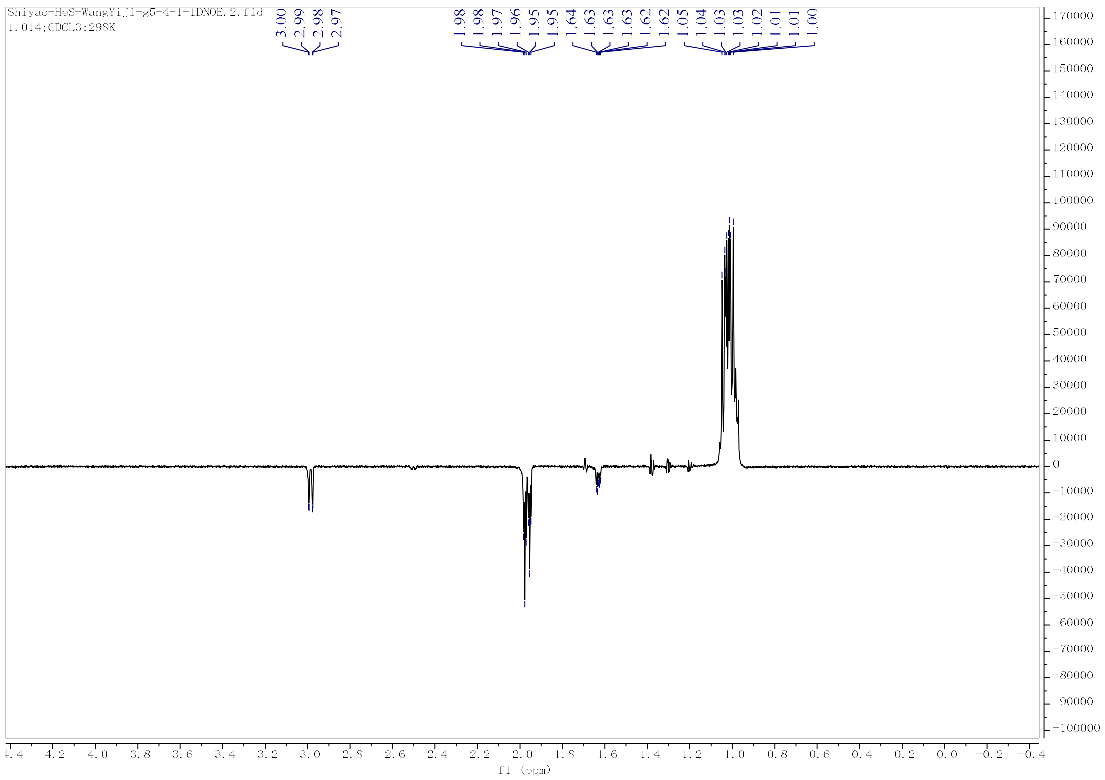

Figure S10 1D NOE spectrum of **1** in $CDCl_3$, 600 MHz (H-13b)

Functional		Solvent?		Basis Set		Type of Data
mPW1PW91		PCM		6-31+G(d, p)		Unscaled Shifts
		DP4+	0.00%	100.00%	-	-	-
Nuclei	sp2?	Experimenta	Isomer 1	Isomer 2	Isomer 3	Isomer 4	Isomer 5
C	x	148.9	153.7	153.3			
C	x	139.4	139.0	138.1			
C	x	137.9	140.5	138.9			
C	x	129.9	129.7	132.8			
C	x	122.2	124.6	125.5			
C	x	109.7	106.3	106.8			
C		73.4	80.5	80.5			
C		72.3	80.5	79.1			
C		65.6	72.8	74.9			
C		61.1	74.4	72.4			
C		48.7	51.2	51.5			
C		46.9	50.16	47.94			
C		38	40.86	39.29			
C		29.3	39.49	32.70			
C		36.1	32.82	39.38			
C		22.4	26.04	25.91			
C		15.5	23.72	16.99			
C		31.1	31.10	31.75			
C		27.4	28.93	27.13			
C		21	16.86	24.39			

Functional	Solvent?	Basis Set	Type of Data			
mPW1PW91	PCM	6-31+G(d, p)	Unscaled Shifts			
	Isomer 1	Isomer 2	Isomer 3	Isomer 4	Isomer 5	Isomer 6
sDP4+ (H data)	-	-	-	-	-	-
sDP4+ (C data)	0.00%	100.00%	-	-	-	-
sDP4+ (all data)	0.00%	100.00%	-	-	-	-
uDP4+ (H data)	-	-	-	-	-	-
uDP4+ (C data)	0.00%	100.00%	-	-	-	-
uDP4+ (all data)	0.00%	100.00%	-	-	-	-
DP4+ (H data)	-	-	-	-	-	-
DP4+ (C data)	0.00%	100.00%	-	-	-	-
DP4+ (all data)	0.00%	100.00%	-	-	-	-

Figure S11 Detailed DP4+ probability (calculated at PCM/mPW1PW91/6-31+G (d, p) level) for compound **1**. Isomer **1** is 1*R*,4*S*,8*R*,11*S*,12*S*, isomer **2** is 1*R*,4*R*,8*R*,11*S*,12*S*

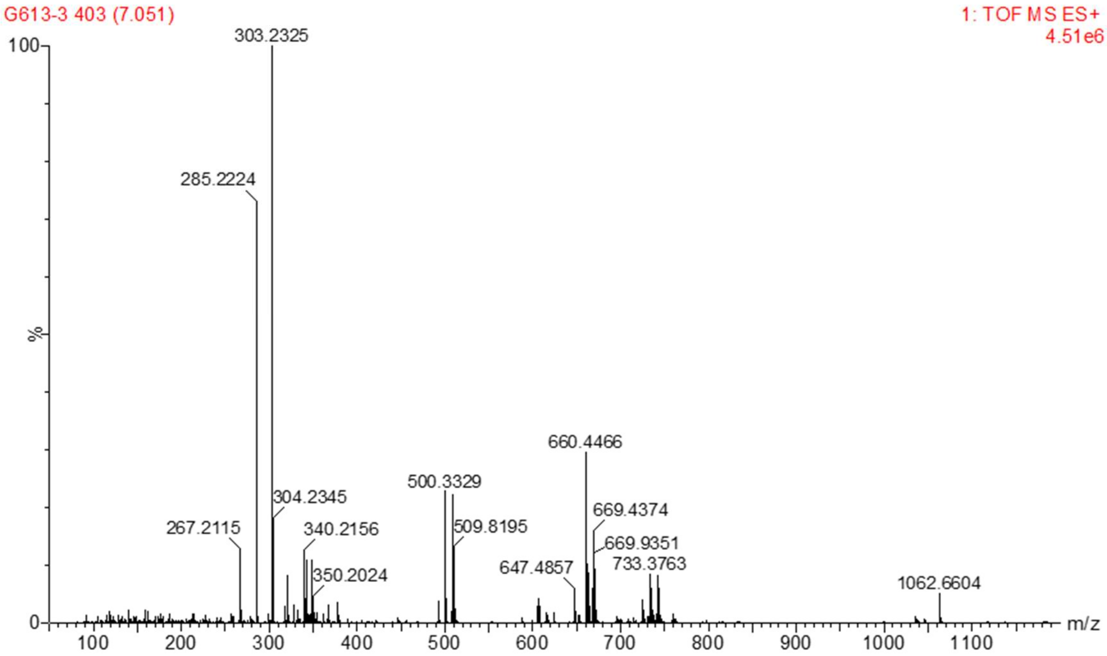

Figure S12 HRESIMS spectrum of compound **2**

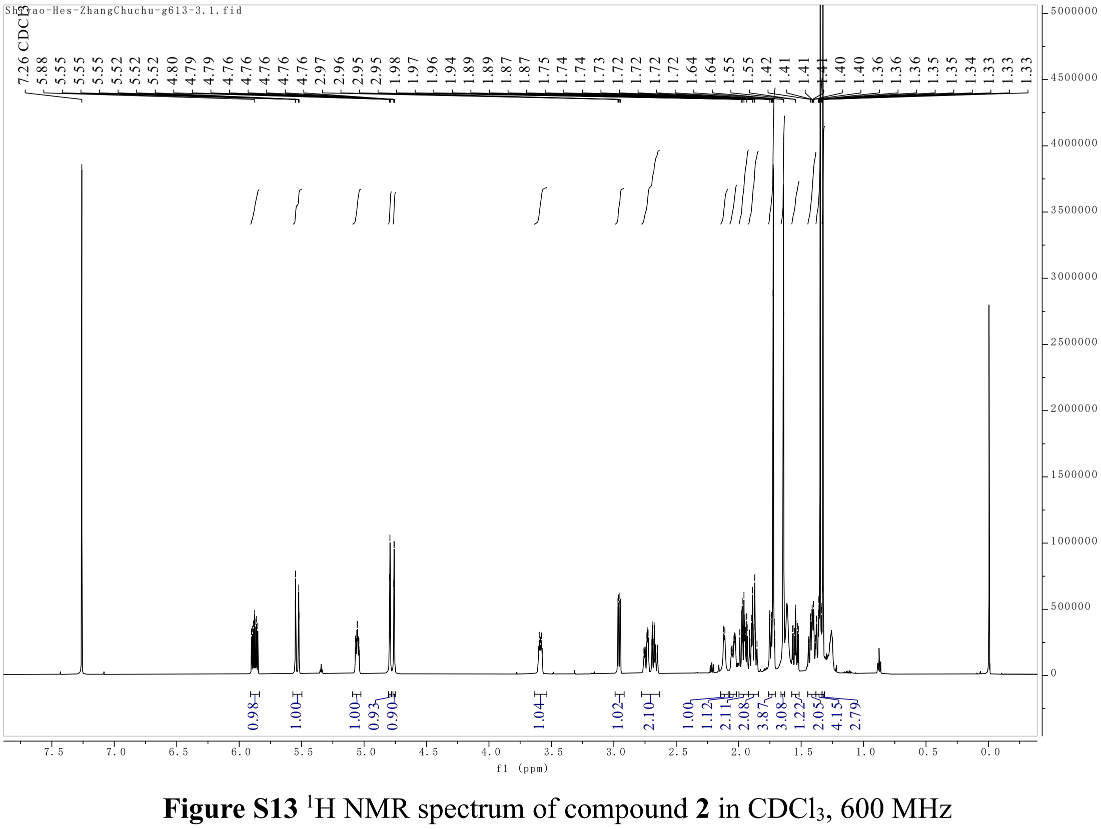

Figure S13 ^{1}H NMR spectrum of compound **2** in $CDCl_3$, 600 MHz

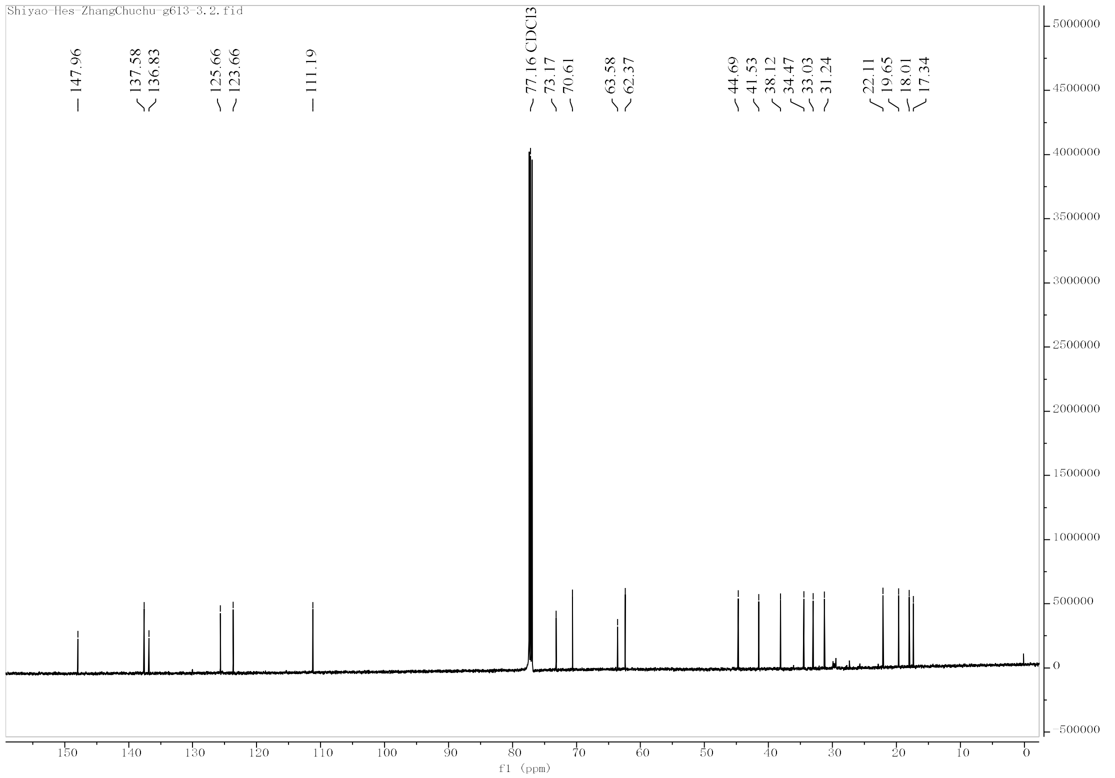

Figure S14 ^{13}C NMR spectrum of compound **2** in $CDCl_3$, 150 MHz

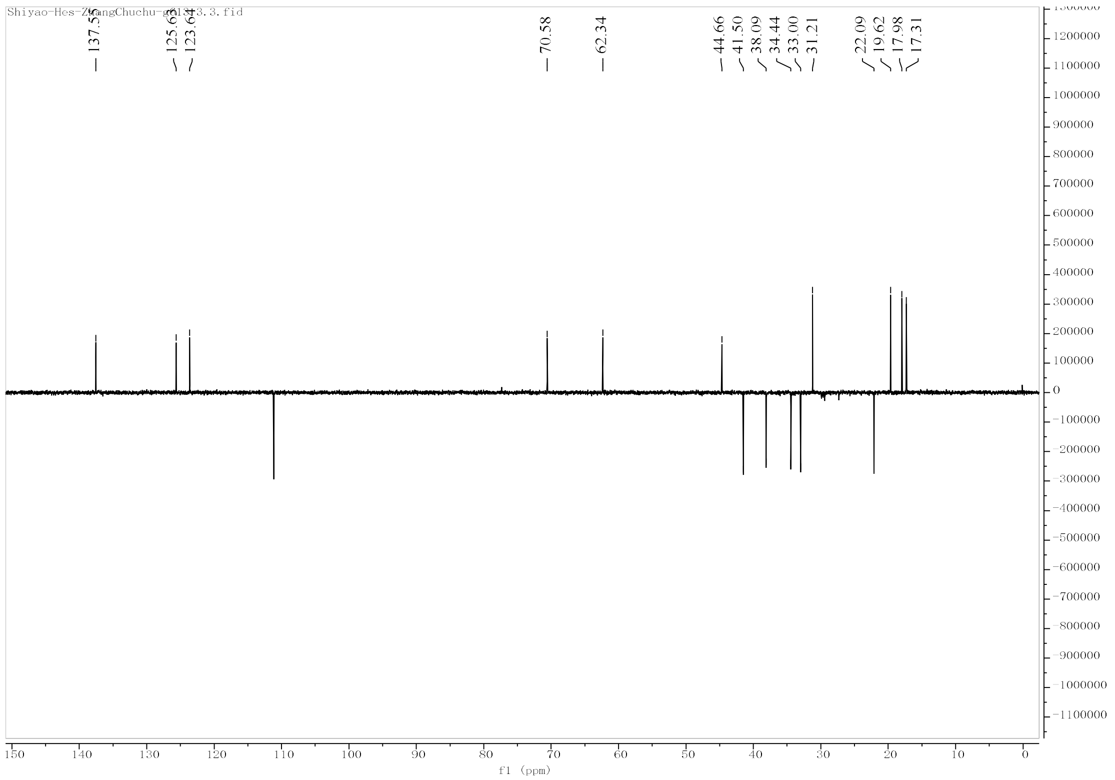

Figure S15 DEPT 135 spectrum of **2** in $CDCl_3$, 150 MHz

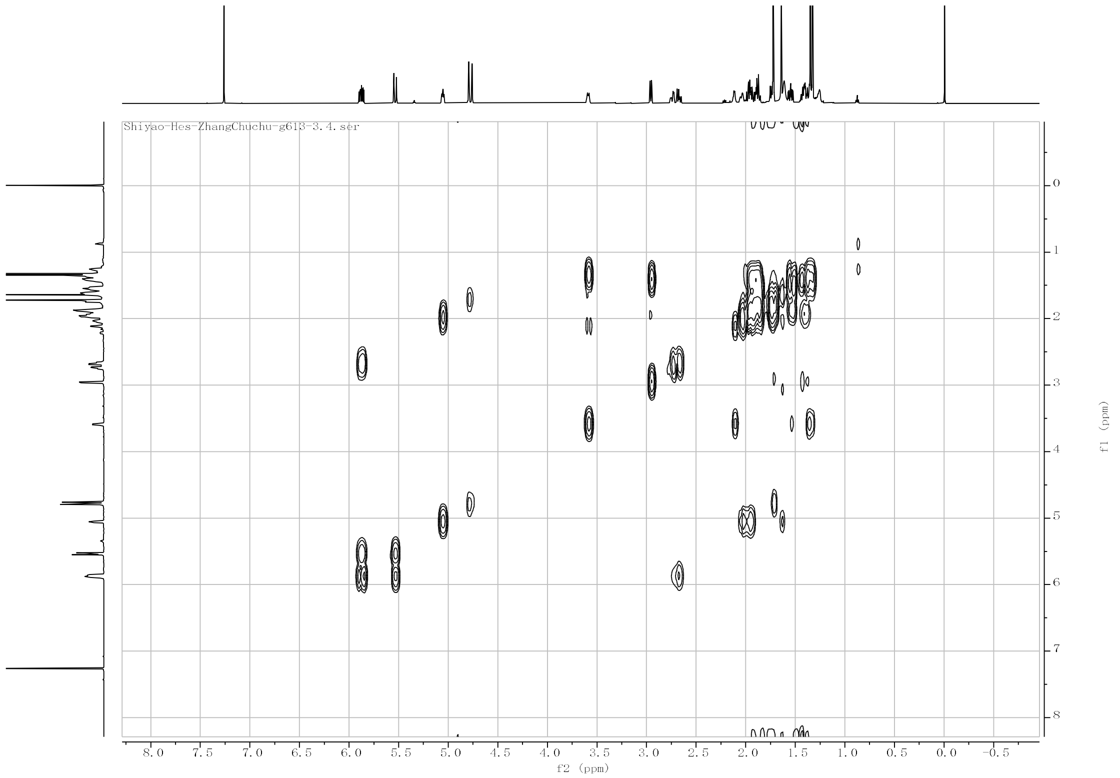

Figure S16 ^{1}H-^{1}H COSY spectrum of **2** in $CDCl_3$, 600 MHz

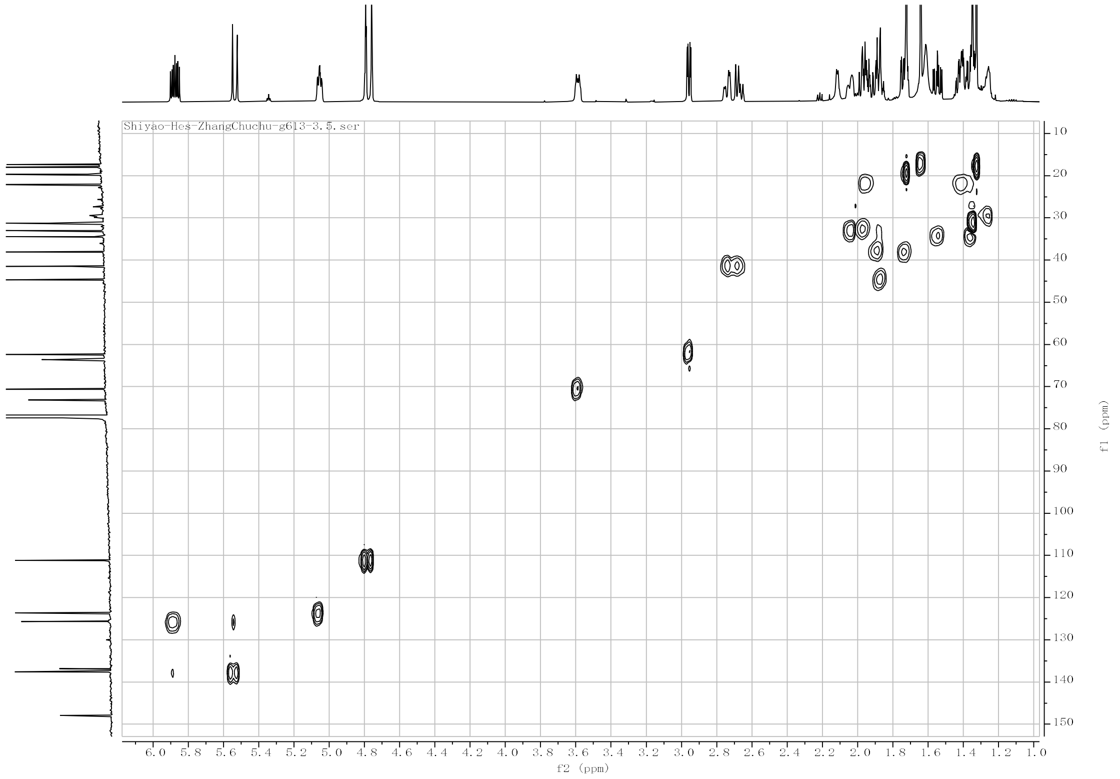

Figure S17 HSQC spectrum of **2** in $CDCl_3$, 150 MHz

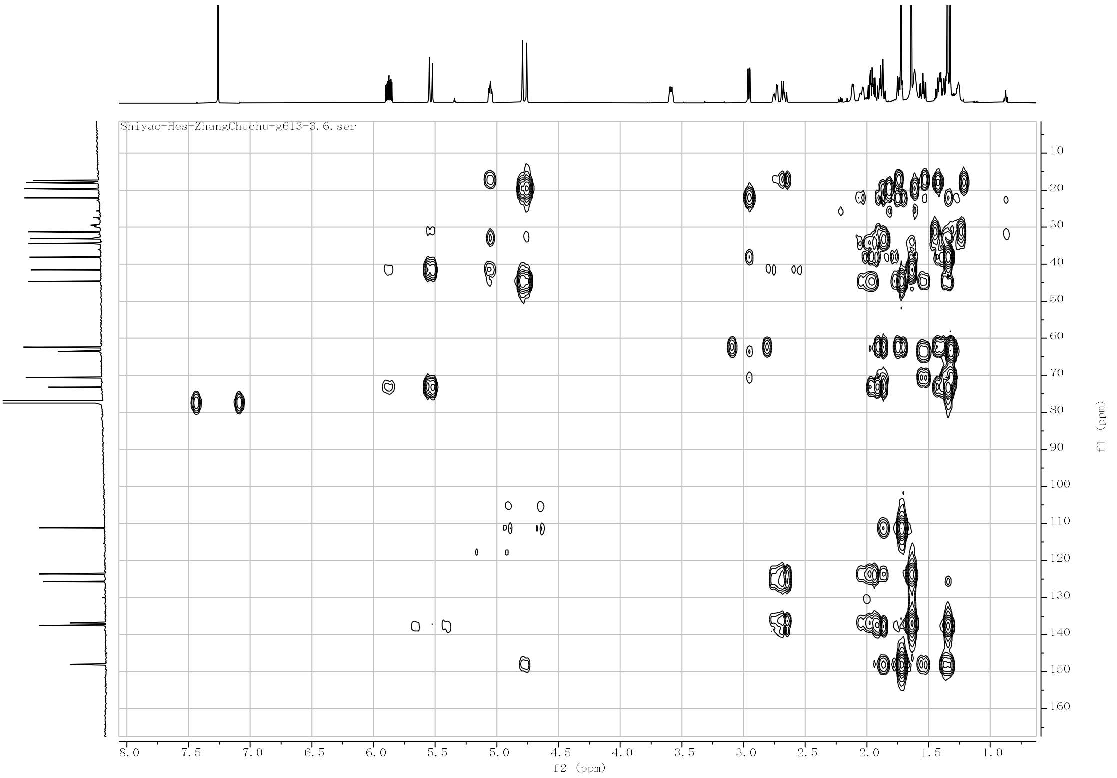

Figure S18 HMBC spectrum of **2** in $CDCl_3$, 150 MHz

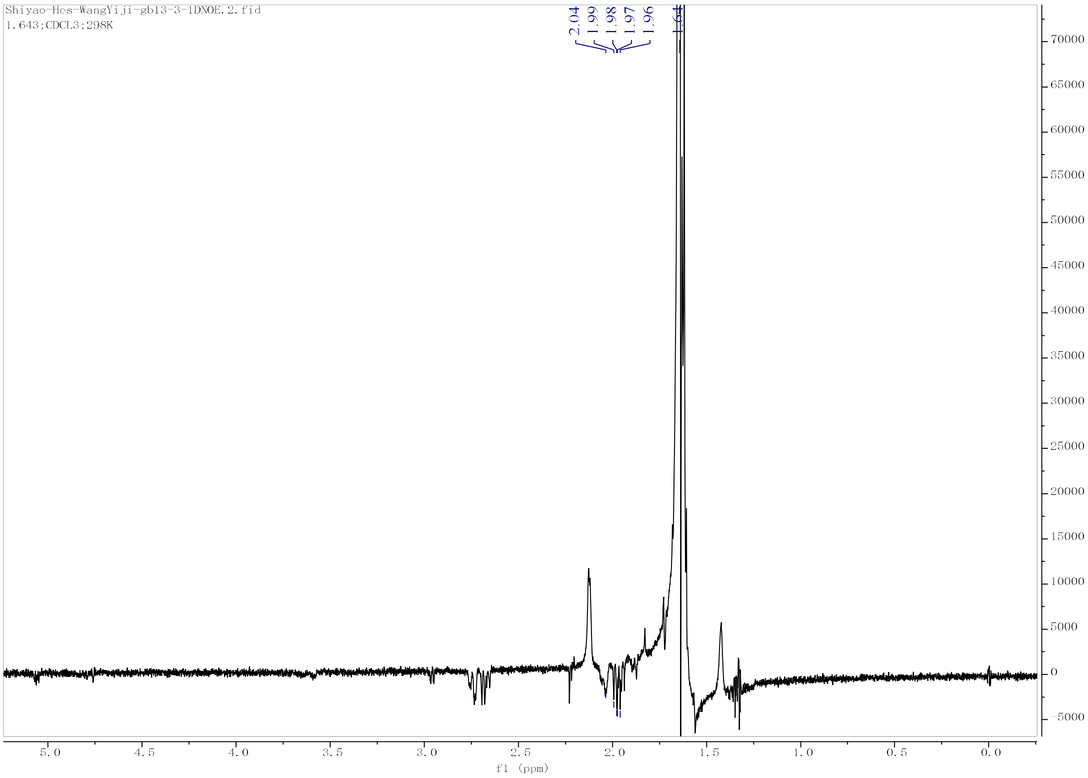

Figure S19 1D NOE spectrum of **2** in $CDCl_3$, 600 MHz (H_3-18)

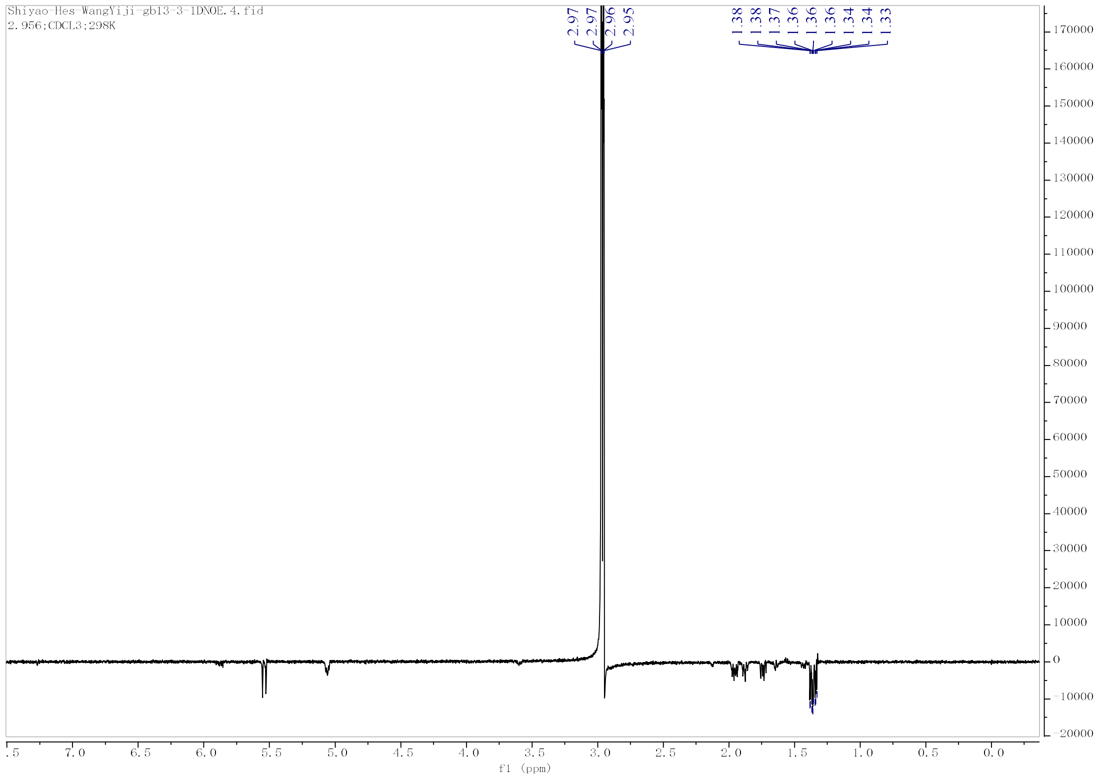

Figure S20 1D NOE spectrum of **2** in $CDCl_3$, 600 MHz (H-11)

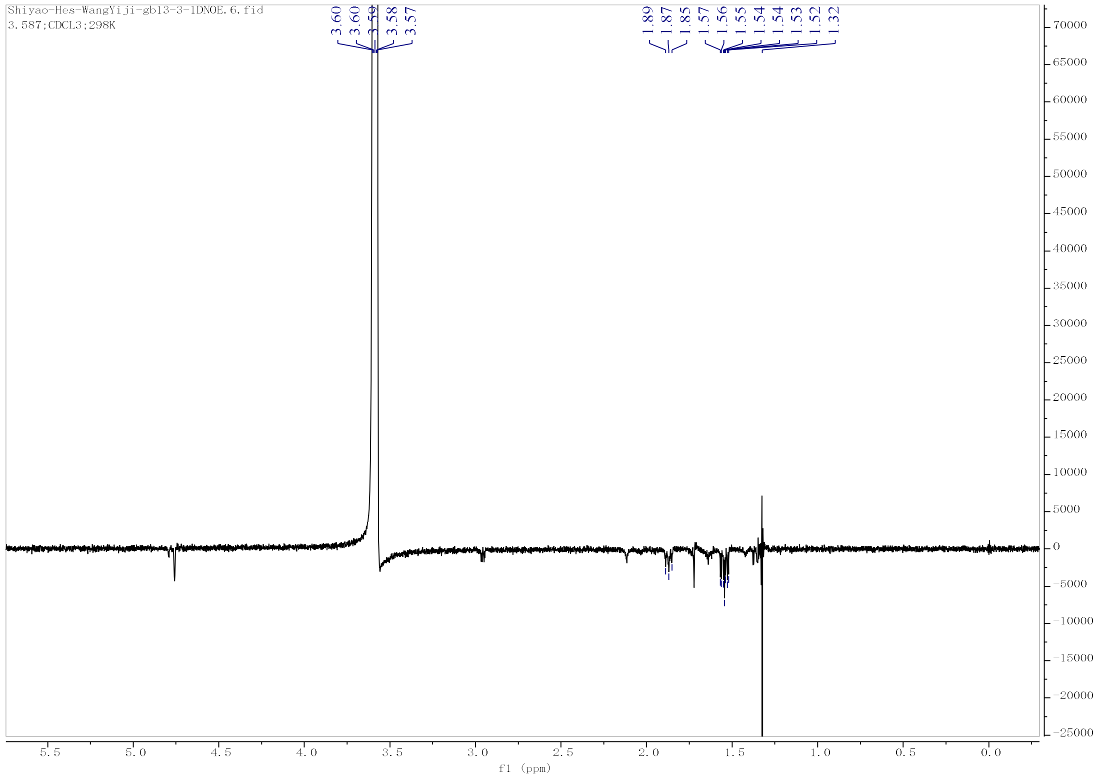

Figure S21 1D NOE spectrum of **2** in $CDCl_3$, 600 MHz (H-13)

		DP4+	0.00%	0.00%	100.00%	0.00%
Nuclei	sp2?	:xperimenta	Isomer 1	Isomer 2	Isomer 3	Isomer 4
C	x	148	155.2	157.0	156.5	155.9
C	x	136.8	139.1	141.4	137.8	143.4
C	x	137.6	134.6	137.1	137.7	137.4
C	x	125.7	126.6	129.8	128.1	126.6
C	x	123.7	123.8	125.4	128.5	124.0
C	X	111.2	107.5	109.7	105.9	106.3
C		70.6	85.3	85.3	80.1	80.7
C		73.2	79.9	80.4	79.5	79.7
C		62.4	75.1	70.9	72.1	75.2
C		63.6	75.1	73.8	80.3	75.7
C		44.7	50.7	55.3	45.9	45.7
C		41.5	48.06	48.26	44.63	41.26
C		38.1	41.04	39.70	40.70	43.12
C		33	38.49	36.68	37.42	40.02
C		34.5	38.01	37.63	39.60	40.69
C		31.2	31.59	24.96	31.23	29.84
C		22.1	25.86	26.85	25.20	26.27
C		19.7	19.87	19.66	25.31	25.26
C		17.3	17.24	17.38	18.31	19.72
C		18	12.08	12.67	11.88	11.99

Functional	Solvent?	Basis Set	
mPW1PW91	PCM	6-31+G(d,p)	

	Isomer 1	Isomer 2	Isomer 3	Isomer 4
sDP4+ (H data)	–	–	–	–
sDP4+ (C data)	0.02%	0.05%	99.93%	0.01%
sDP4+ (all data)	0.02%	0.05%	99.93%	0.01%
uDP4+ (H data)	–	–	–	–
uDP4+ (C data)	8.37%	0.00%	91.52%	0.11%
uDP4+ (all data)	8.37%	0.00%	91.52%	0.11%
DP4+ (H data)	–	–	–	–
DP4+ (C data)	0.00%	0.00%	100.00%	0.00%
DP4+ (all data)	0.00%	0.00%	100.00%	0.00%

Figure S22 Detailed DP4+ probability (calculated at PCM/mPW1PW91/6-31+G (d, p) level) for compound **2**. Isomer **1** is *1*S,*8*S,*11*R,*12R,13*S, isomer **2** is *1*S,*8R,11R,12R,13S*, isomer **3** is *1S,8S,11S,12S,13R*, isomer **4** is 1*S*,8*R*,11*S*,12*S*,13*R*

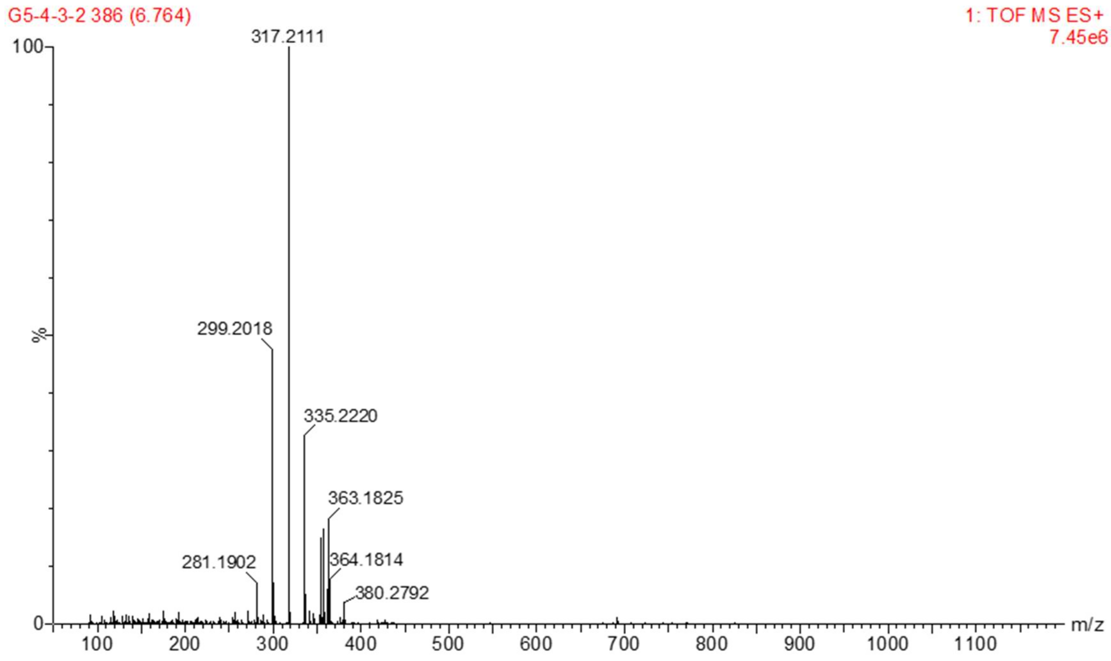

Figure S23 HRESIMS spectrum of compound **3**

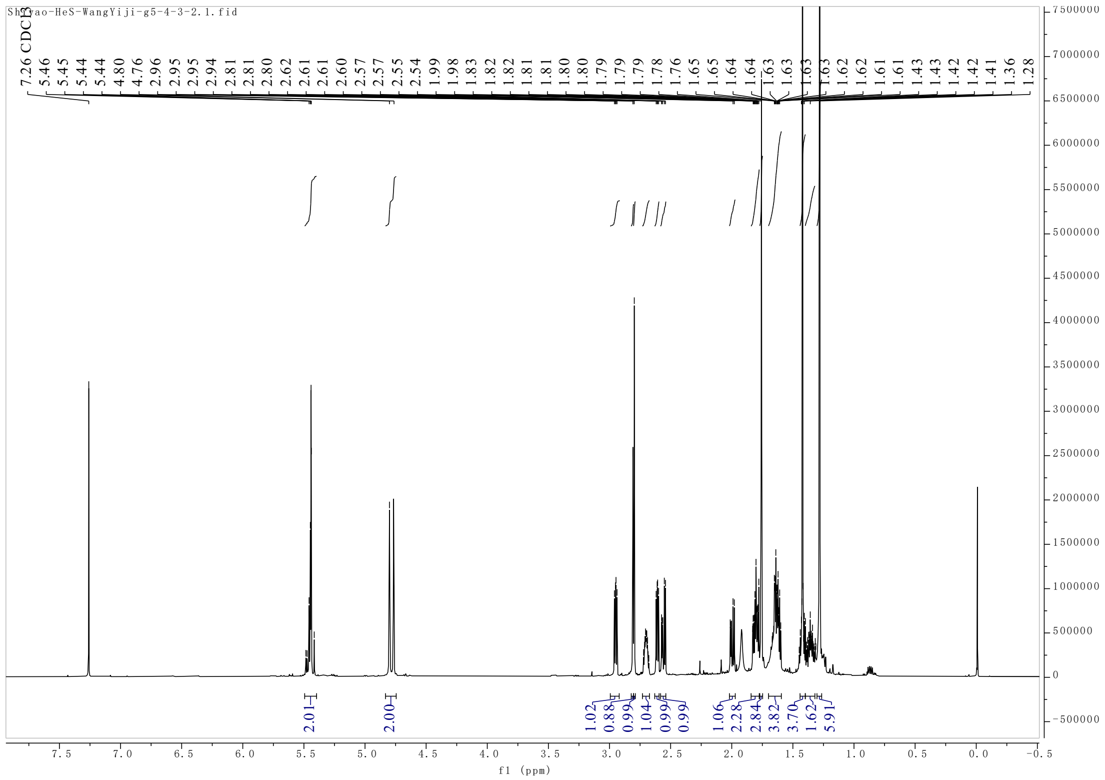

Figure S24 ^{1}H NMR spectrum of compound **3** in $CDCl_3$, 600 MHz

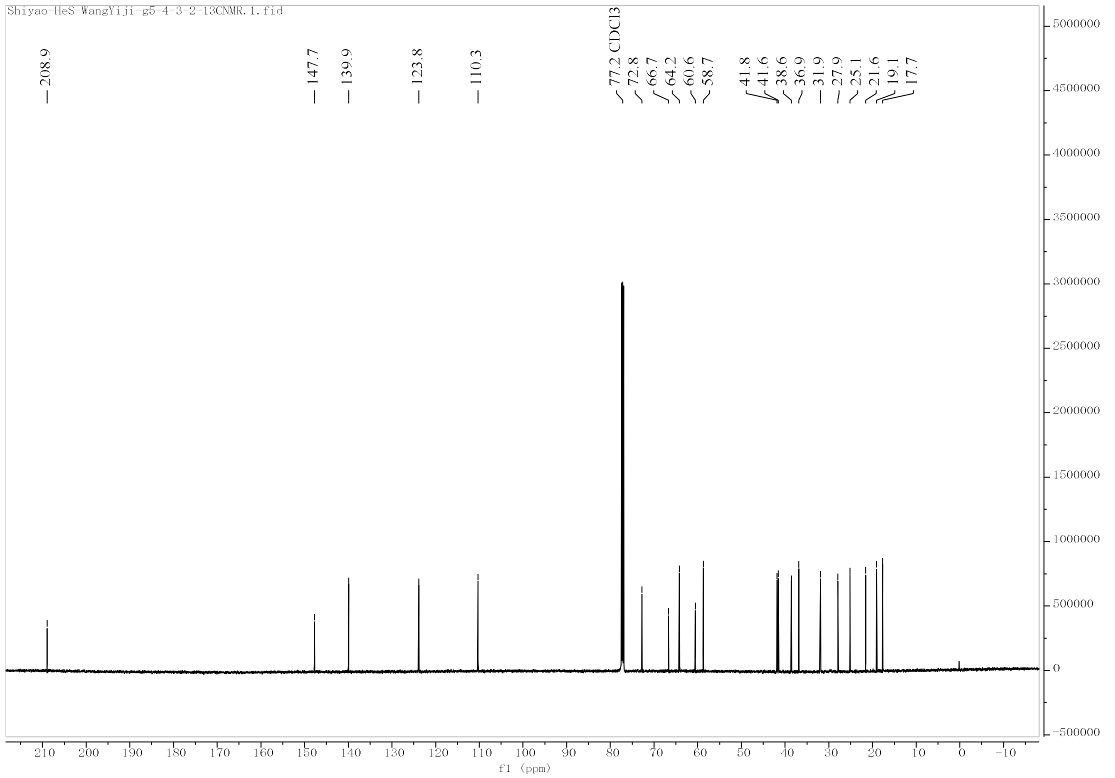

Figure S25 ^{13}C NMR spectrum of compound **3** in $CDCl_3$, 150 MHz

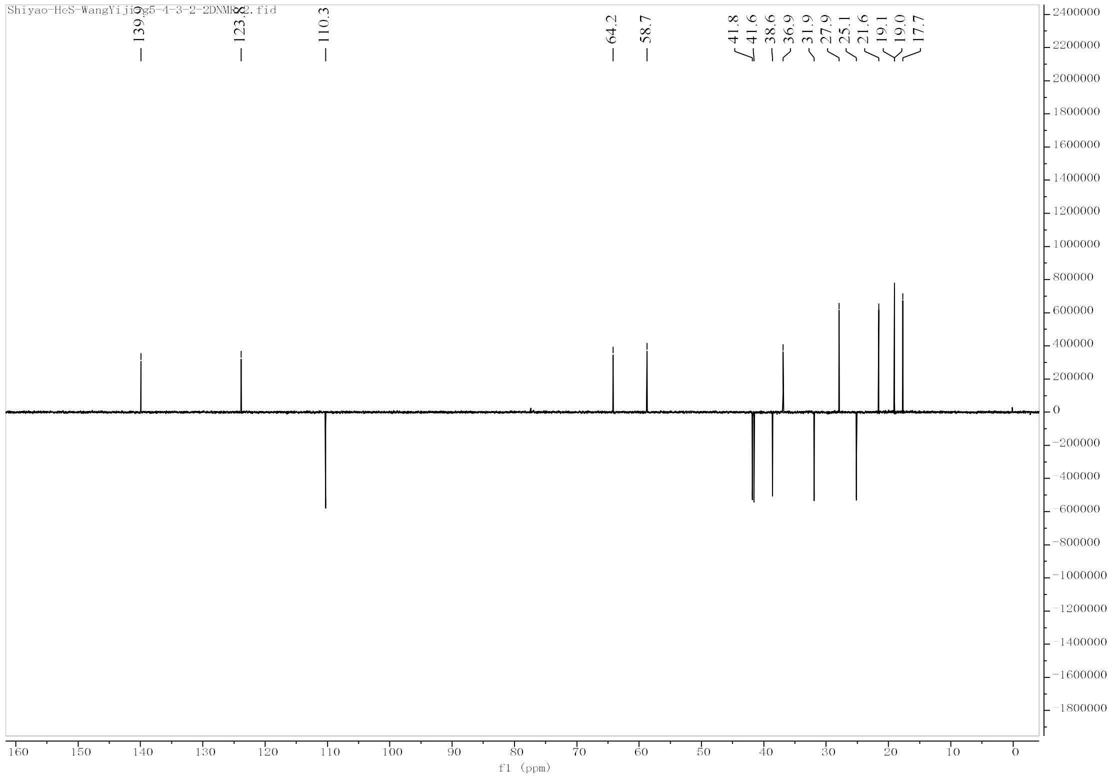

Figure S26 DEPT 135 spectrum of **3** in $CDCl_3$, 150 MHz

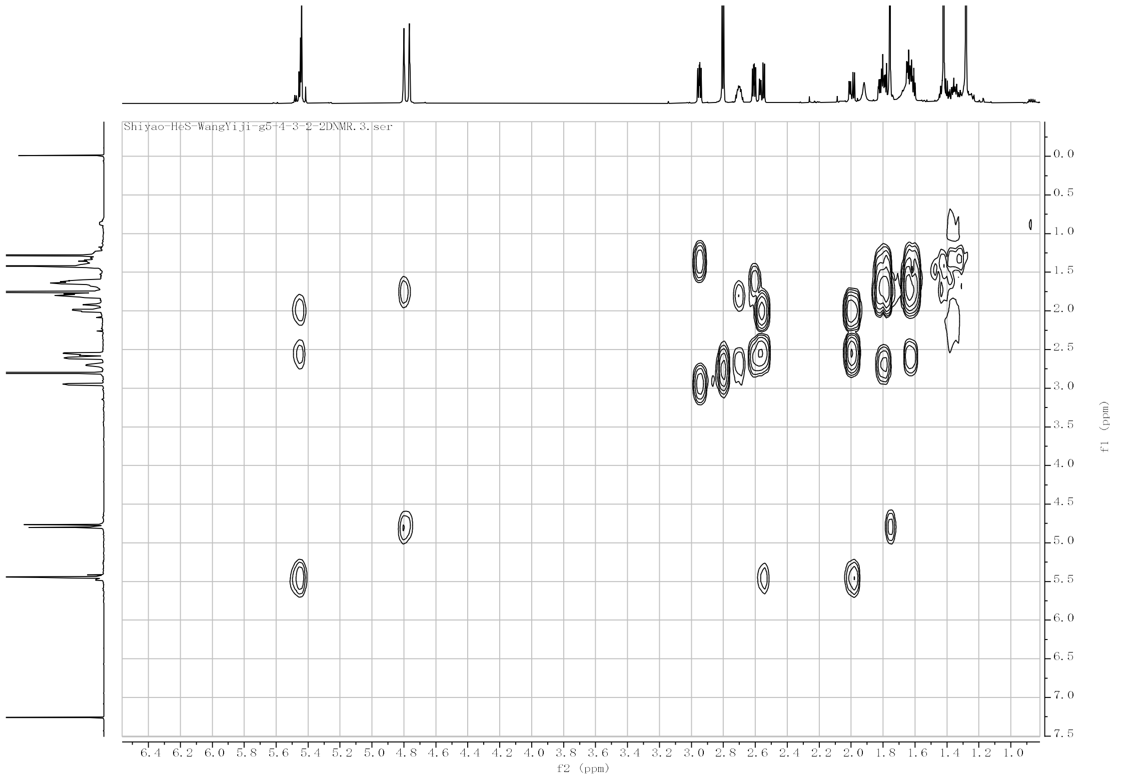

Figure S27 ^{1}H-^{1}H COSY spectrum of **3** in $CDCl_3$, 600 MHz

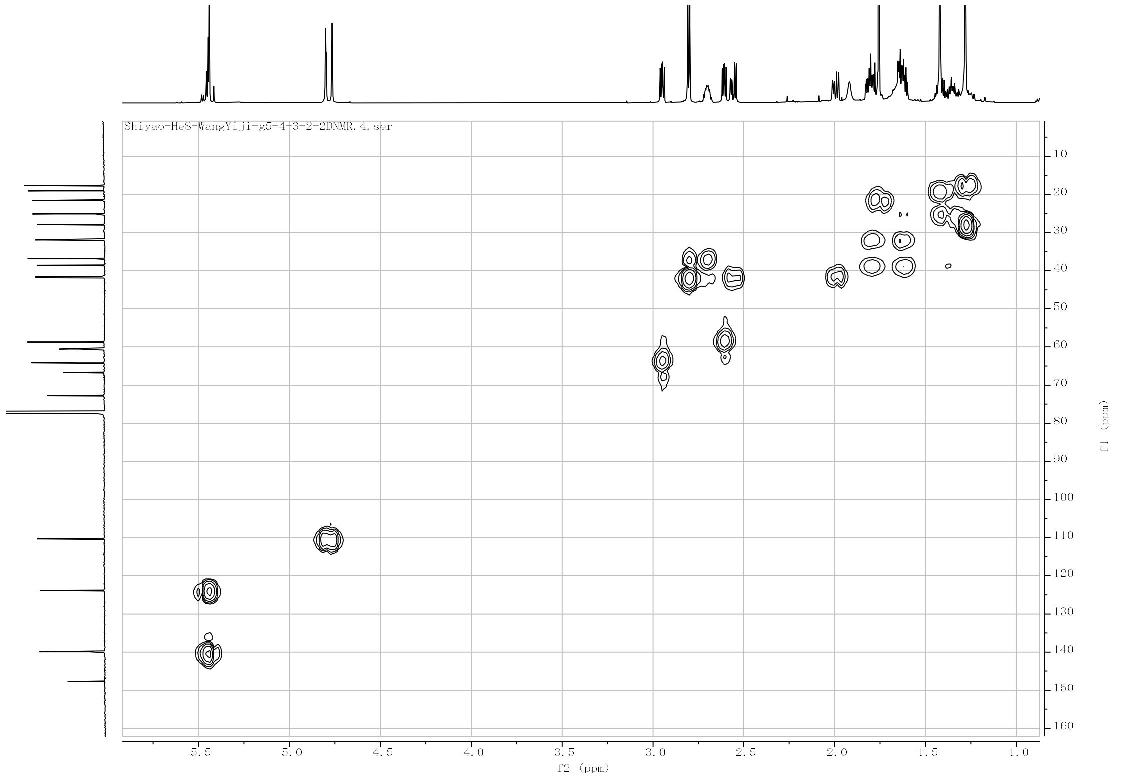

Figure S28 HSQC spectrum of **3** in $CDCl_3$, 150 MHz

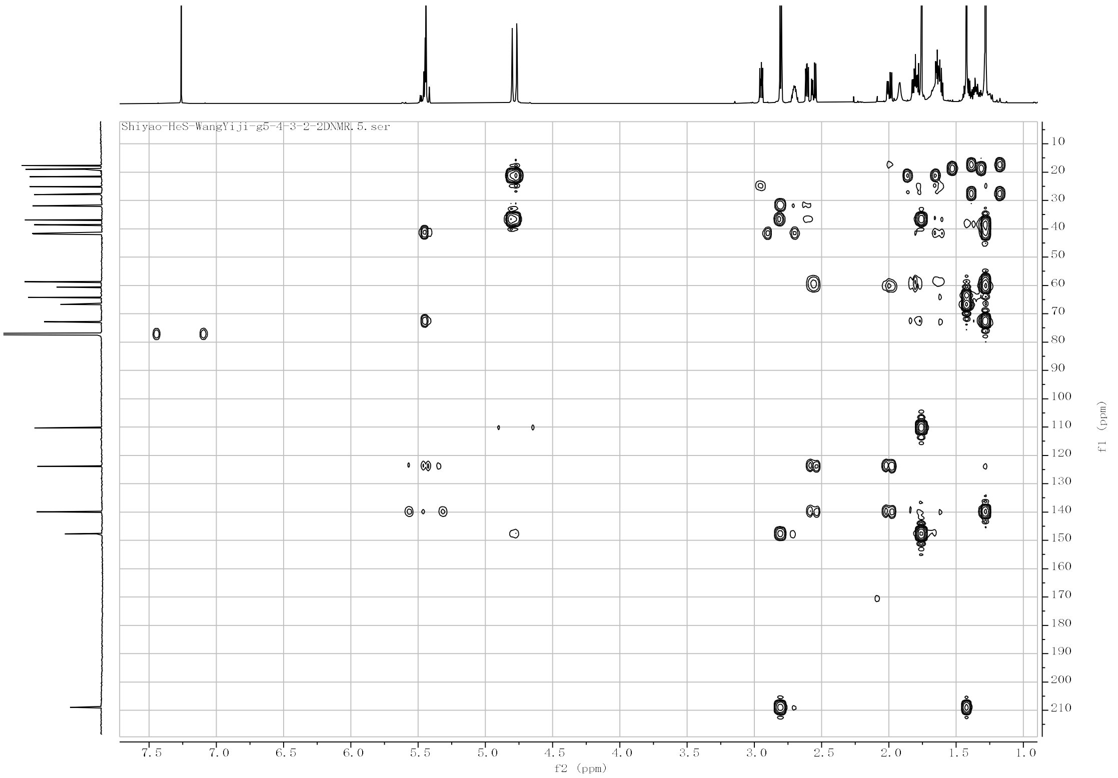

Figure S29 HMBC spectrum of **3** in $CDCl_3$, 150 MHz

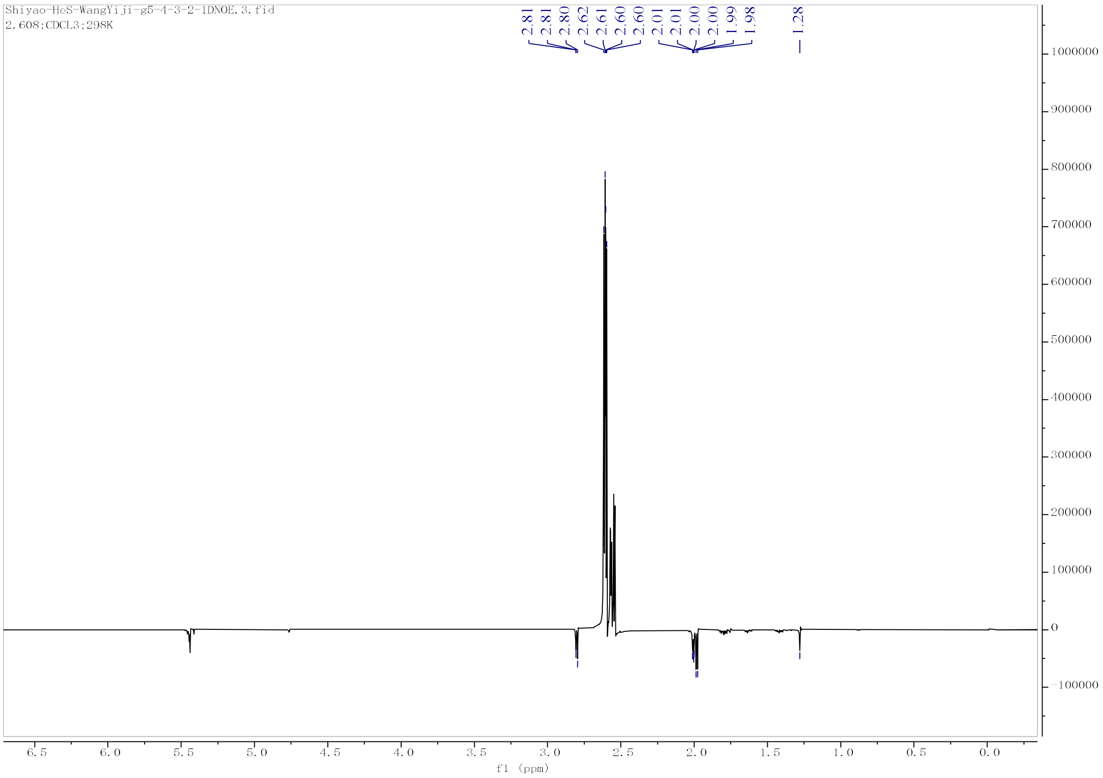

Figure S30 1D NOE spectrum of **3** in $CDCl_3$, 600 MHz (H-3)

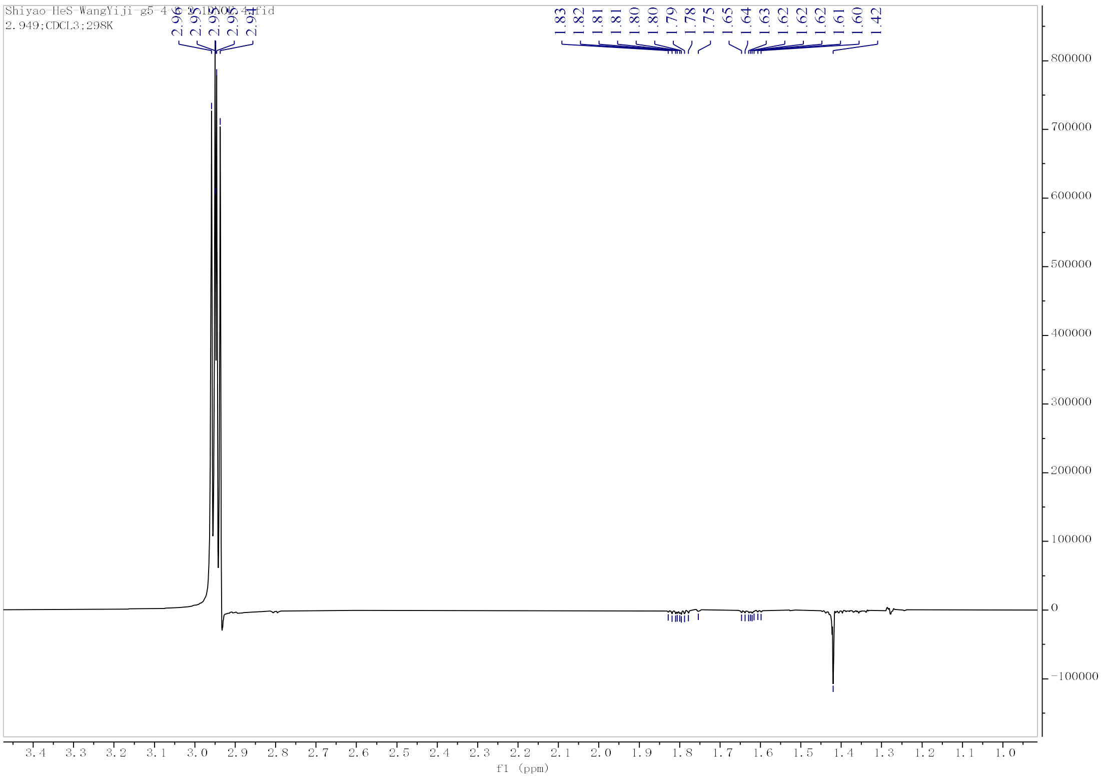

Figure S31 1D NOE spectrum of **3** in $CDCl_3$, 600 MHz (H-11)

Functional		Solvent?		Basis Set		Type of Data	
mPW1PW91		PCM		6-31+G(d,p)		Unscaled Shifts	
		DP4+	0.11%	68.15%	0.00%	31.74%	-
Nuclei	sp2?	xperimenta	Isomer 1	Isomer 2	Isomer 3	Isomer 4	Isomer 5
C	x	208.9	220.2	215.6	215.0	214.1	
C	x	147.7	152.3	152.3	153.0	152.4	
C	x	139.9	134.2	132.9	138.3	134.9	
C	x	123.8	128.0	128.4	128.1	128.2	
C	x	110.3	109.7	109.5	110.4	110.4	
C		72.8	83.5	82.7	80.9	81.4	
C		64.2	75.1	74.6	79.1	77.0	
C		60.6	69.2	70.2	74.0	69.4	
C		66.7	77.2	75.7	70.7	73.9	
C		58.7	71.3	71.9	72.4	71.3	
C		41.8	46.9	46.7	52.1	50.3	
C		36.9	42.36	43.18	48.04	46.55	
C		38.6	39.11	41.03	39.88	40.05	
C		41.6	32.09	34.14	33.44	37.70	
C		31.9	30.06	29.87	31.44	34.64	
C		25.2	28.23	28.46	24.40	27.22	
C		27.9	33.26	32.76	28.01	30.32	
C		17.7	22.95	23.20	23.88	23.30	
C		19.1	21.56	20.73	22.80	20.69	
C		21.6	25.24	25.15	19.01	20.75	

Functional	Solvent?		Basis Set		Type of Data	
mPW1PW91	PCM		6-31+G(d, p)		Unscaled Shifts	
	Isomer 1	Isomer 2	Isomer 3	Isomer 4	Isomer 5	Isomer 6
sDP4+ (H data)	–	–	–	–	–	–
sDP4+ (C data)	3.41%	84.01%	0.00%	12.58%	–	–
sDP4+ (all data)	3.41%	84.01%	0.00%	12.58%	–	–
uDP4+ (H data)	–	–	–	–	–	–
uDP4+ (C data)	0.99%	24.09%	0.00%	74.92%	–	–
uDP4+ (all data)	0.99%	24.09%	0.00%	74.92%	–	–
DP4+ (H data)	–	–	–	–	–	–
DP4+ (C data)	0.11%	68.15%	0.00%	31.74%	–	–
DP4+ (all data)	0.11%	68.15%	0.00%	31.74%	–	–

Figure S32 Detailed DP4+ probability (calculated at PCM/mPW1PW91/6-31+G (d, p) level) for compound **3**. Isomer **1** is 1*S*,3*R*,4*S*,8*R*,11*R*,12*R*, isomer **2** is 1*S*,3*R*,4*S*,8*R*,11*S*,12*S*, isomer **3** is 1*S*,3*R*,4*S*,8*S*,11*R*,12*R*, isomer **4** is 1*S*,3*R*,4*S*,8*S*,11*S*,12*S*

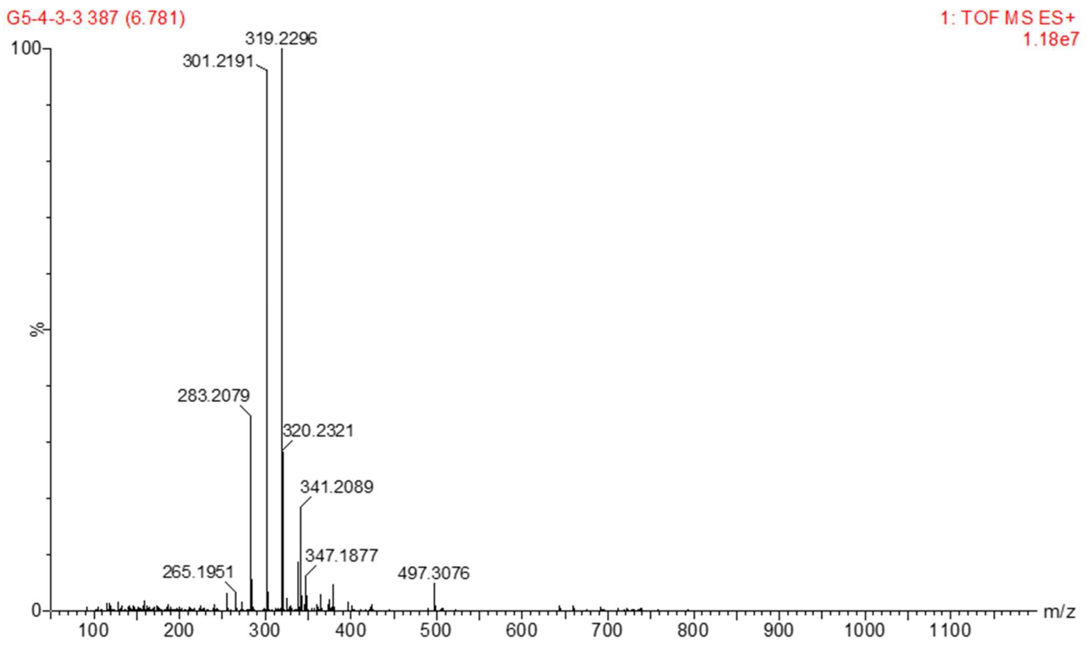

Figure S33 HRESIMS spectrum of compound **4**

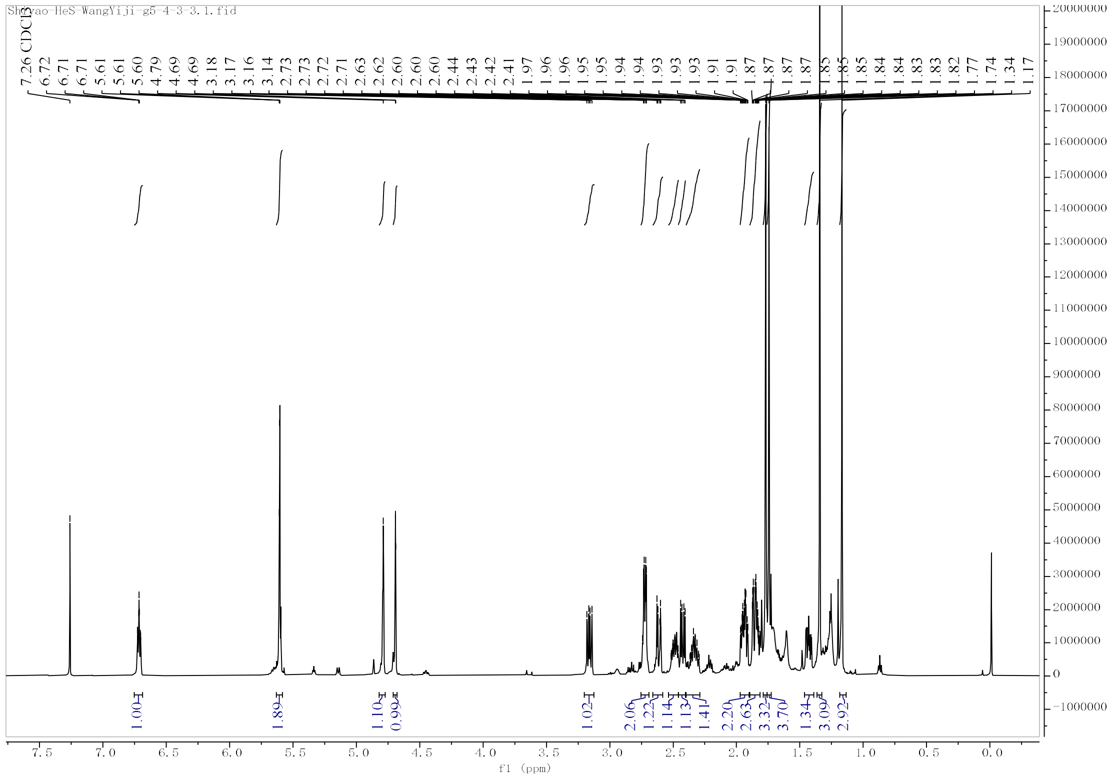

Figure S34 ^{1}H NMR spectrum of compound **4** in $CDCl_3$, 600 MHz

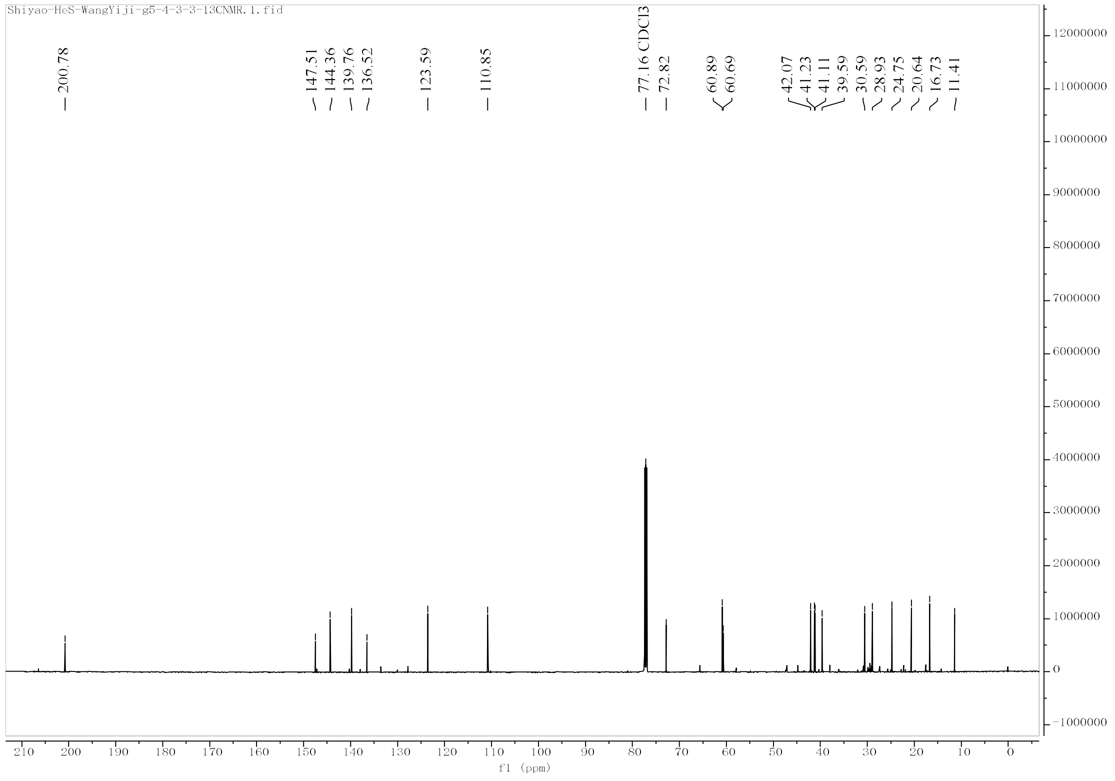

Figure S35 ^{13}C NMR spectrum of compound **4** in $CDCl_3$, 150 MHz

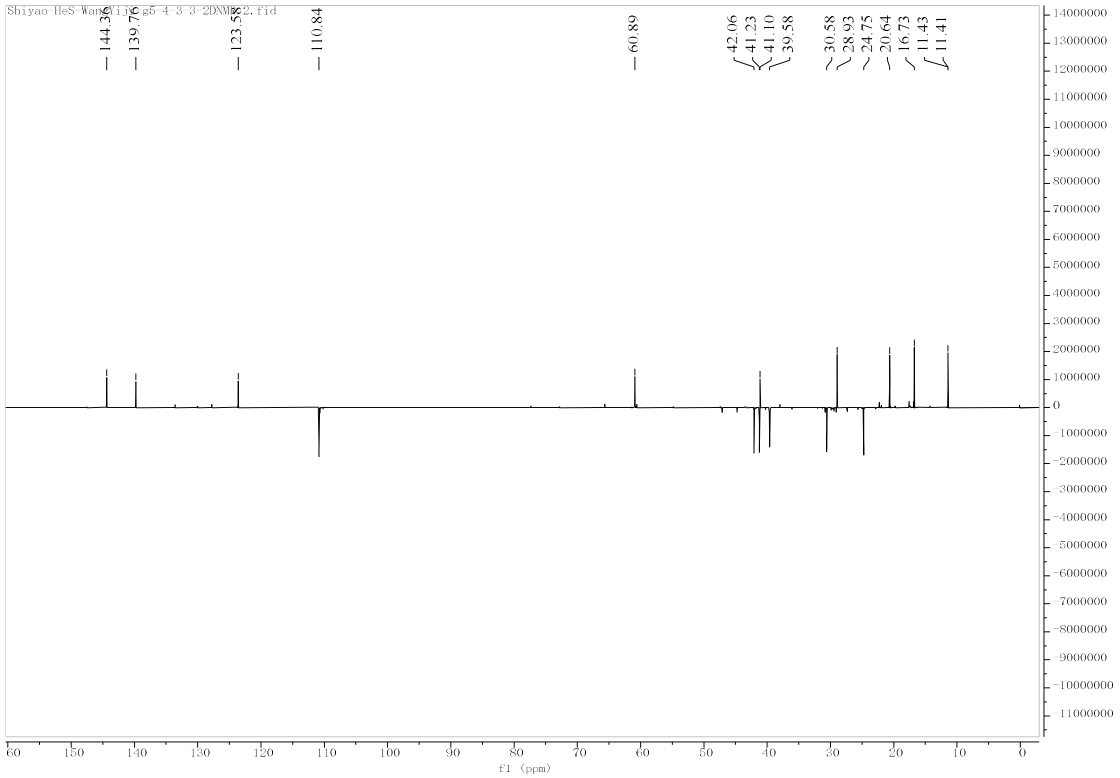

Figure S36 DEPT 135 spectrum of **4** in $CDCl_3$, 150 MHz

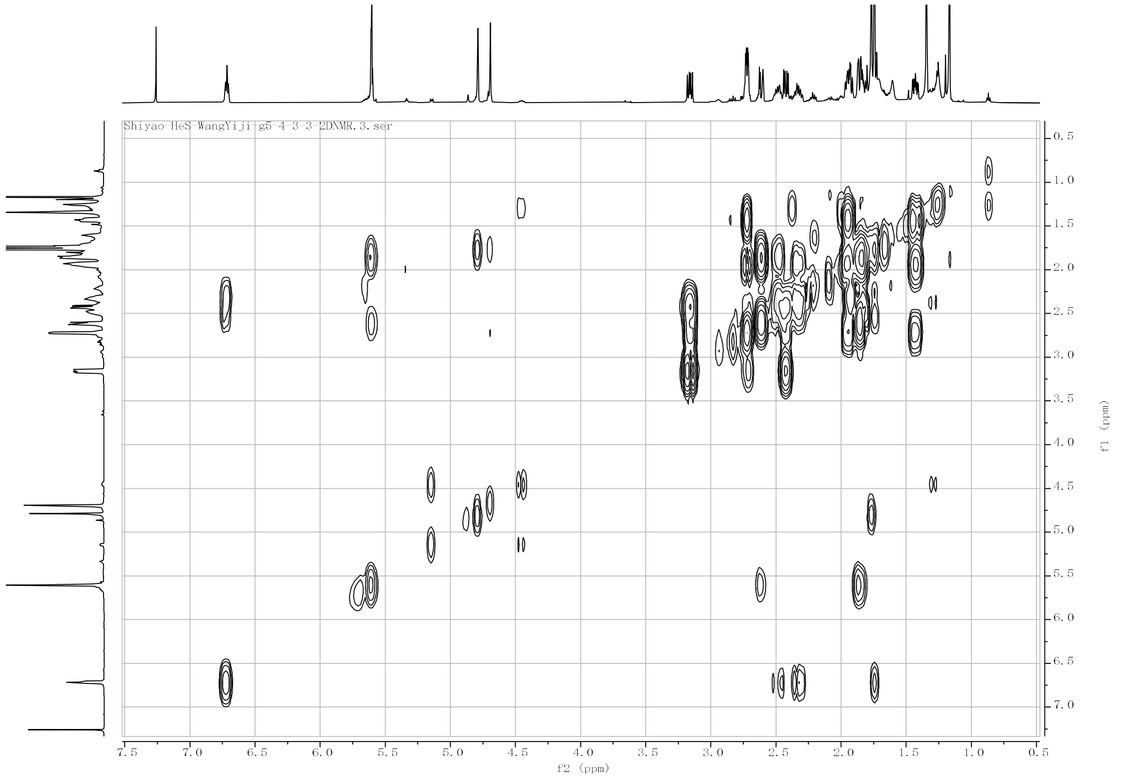

Figure S37 ^1H-^1H COSY spectrum of **4** in $CDCl_3$, 600 MHz

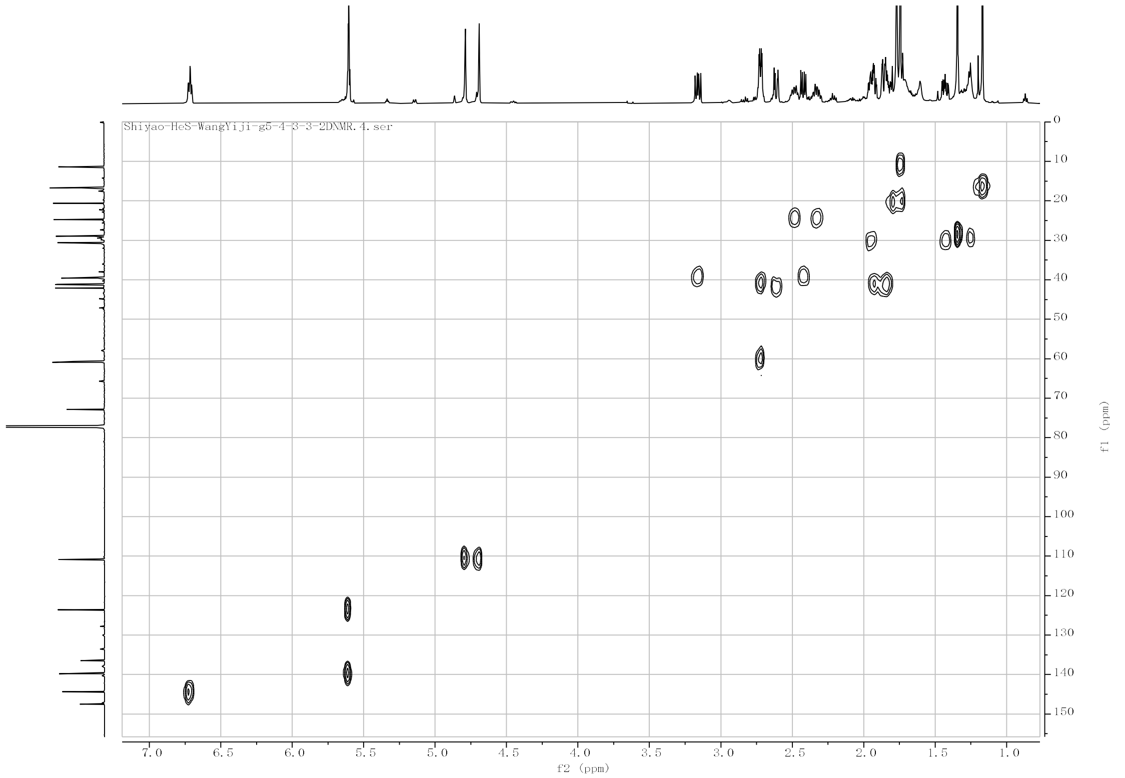

Figure S38 HSQC spectrum of **4** in $CDCl_3$, 150 MHz

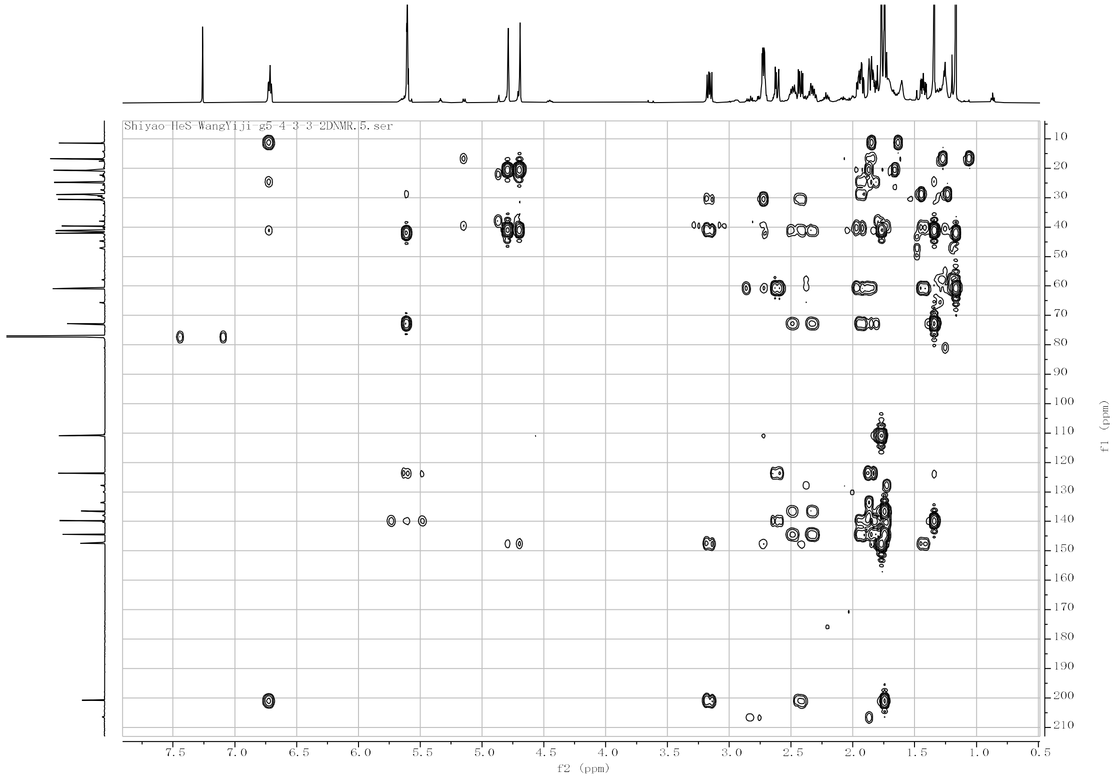

Figure S39 HMBC spectrum of **4** in $CDCl_3$, 150 MHz

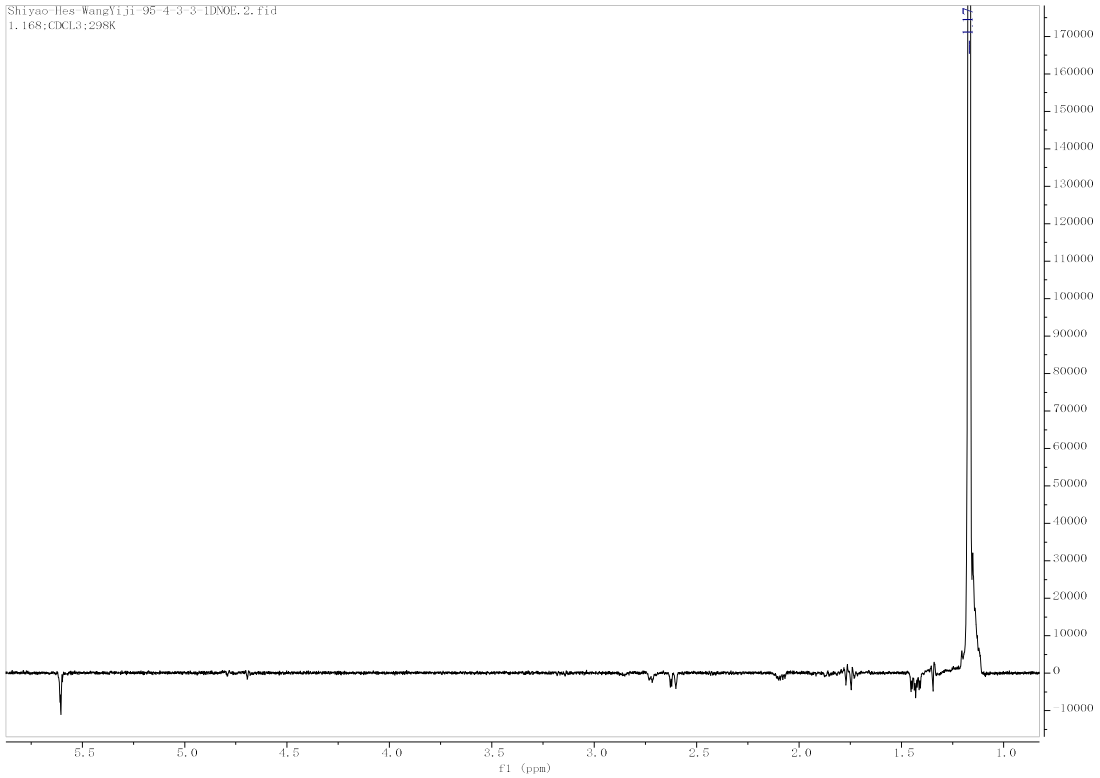

Figure S40 1D NOE spectrum of **4** in $CDCl_3$, 600 MHz (H_3-18)

Functional		Solvent?		Basis Set		Type of Data
mPW1PW91		PCM		6-31+G(d,p)		Unscaled Shifts
		DP4+	0.00%	0.03%	0.07%	99.90%	–
Nuclei	sp2?	Experimenta	Isomer 1	Isomer 2	Isomer 3	Isomer 4	Isomer 5
C	x	200.8	207.9	208.8	207.3	206.1	
C	x	147.5	153.5	148.7	153.9	152.2	
C	x	144.4	150.3	150.5	149.6	145.1	
C	x	136.5	134.3	132.9	135.3	141.2	
C	x	139.8	133.8	133.6	135.9	136.8	
C	x	123.6	128.8	128.8	128.1	131.3	
C	x	110.9	108.2	110.8	107.9	109.5	
C		72.8	84.0	84.2	82.4	81.5	
C		60.7	73.2	74.1	73.2	73.3	
C		60.9	71.7	70.4	74.6	68.7	
C		41.1	51.8	50.2	49.9	50.3	
C		41.2	44.93	46.69	44.91	46.08	
C		39.6	42.78	42.10	41.81	40.32	
C		42.1	41.75	36.85	41.62	37.62	
C		30.6	38.02	35.73	37.97	37.25	
C		28.9	32.97	32.67	32.26	30.78	
C		24.8	30.46	29.57	30.33	29.45	
C		20.6	19.29	25.25	20.53	20.02	
C		16.7	17.93	21.45	18.27	21.28	
C		11.4	13.10	13.08	13.59	14.21	

Functional	Solvent?	Basis Set	Type of Data
mPW1PW91	PCM	6-31+G(d,p)	Unscaled Shifts

	Isomer 1	Isomer 2	Isomer 3	Isomer 4	Isomer 5	Isomer 6
sDP4+ (H data)	–	–	–	–	–	–
sDP4+ (C data)	0.00%	0.49%	0.13%	99.38%	–	–
sDP4+ (all data)	0.00%	0.49%	0.13%	99.38%	–	–
uDP4+ (H data)	–	–	–	–	–	–
uDP4+ (C data)	0.15%	3.87%	34.45%	61.53%	–	–
uDP4+ (all data)	0.15%	3.87%	34.45%	61.53%	–	–
DP4+ (H data)	–	–	–	–	–	–
DP4+ (C data)	0.00%	0.03%	0.07%	99.90%	–	–
DP4+ (all data)	0.00%	0.03%	0.07%	99.90%	–	–

Figure S41 Detailed DP4+ probability (calculated at PCM/mPW1PW91/6-31+G (d, p) level) for compound **4**. Isomer **1** is 1*S*,3*S*,4*S*,8*S*, isomer **2** is 1*S*,3*R*,4*R*,8*S*, isomer **3** is 1*R*,3*S*,4*S*,8*S*, isomer **4** is 1*R*,3*R*,4*R*,8*S*

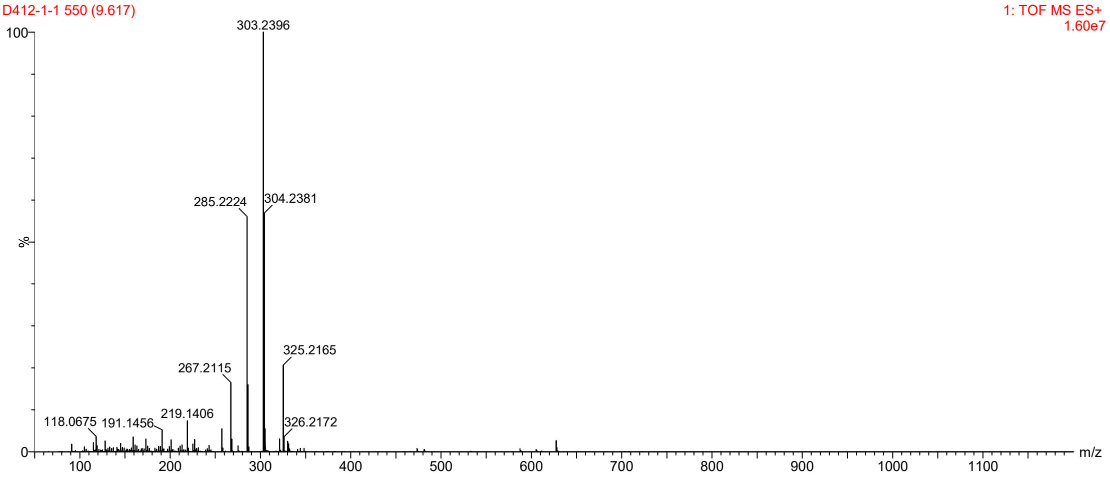

Figure S42 HRESIMS spectrum of compound **5**

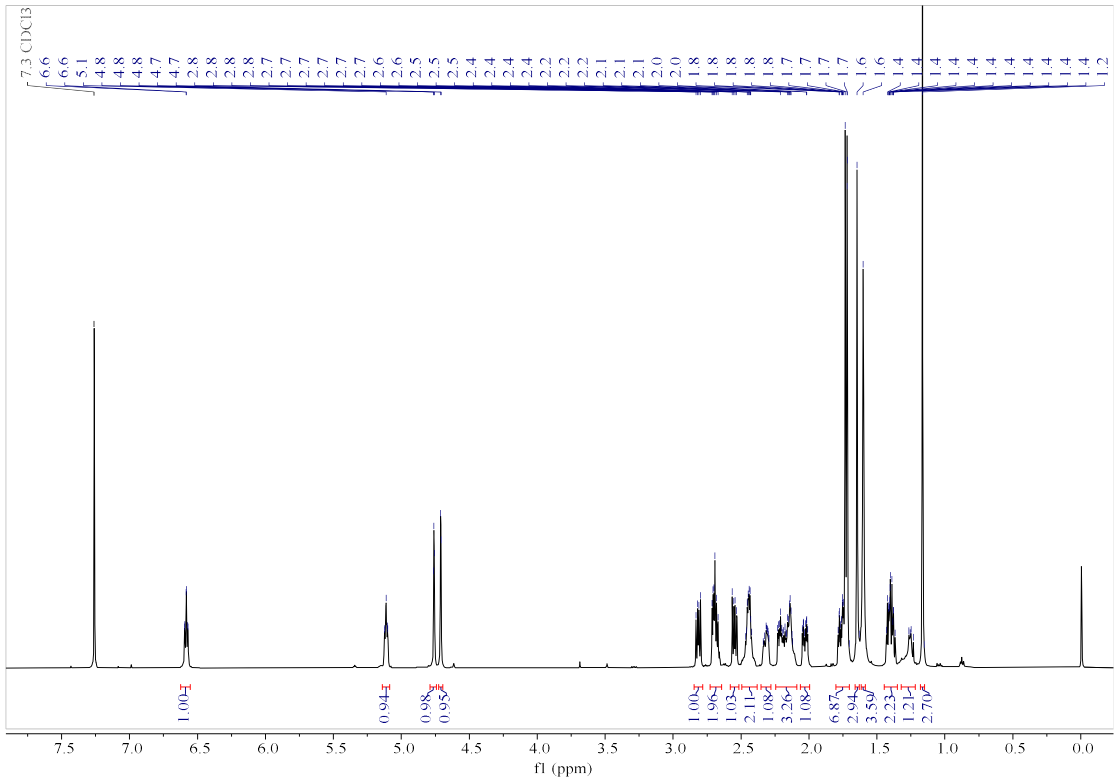

Figure S43 ^{1}H NMR spectrum of compound **5** in $CDCl_3$, 600 MHz

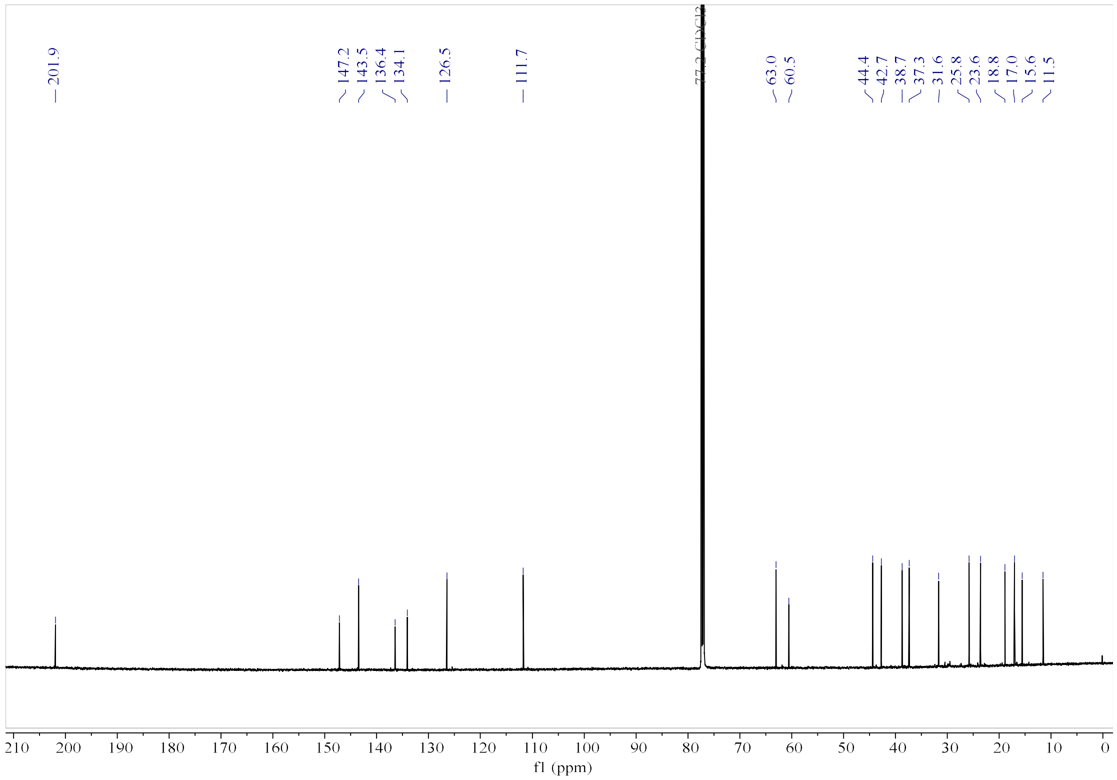

Figure S44 ^{13}C NMR spectrum of compound **5** in $CDCl_3$, 150 MHz

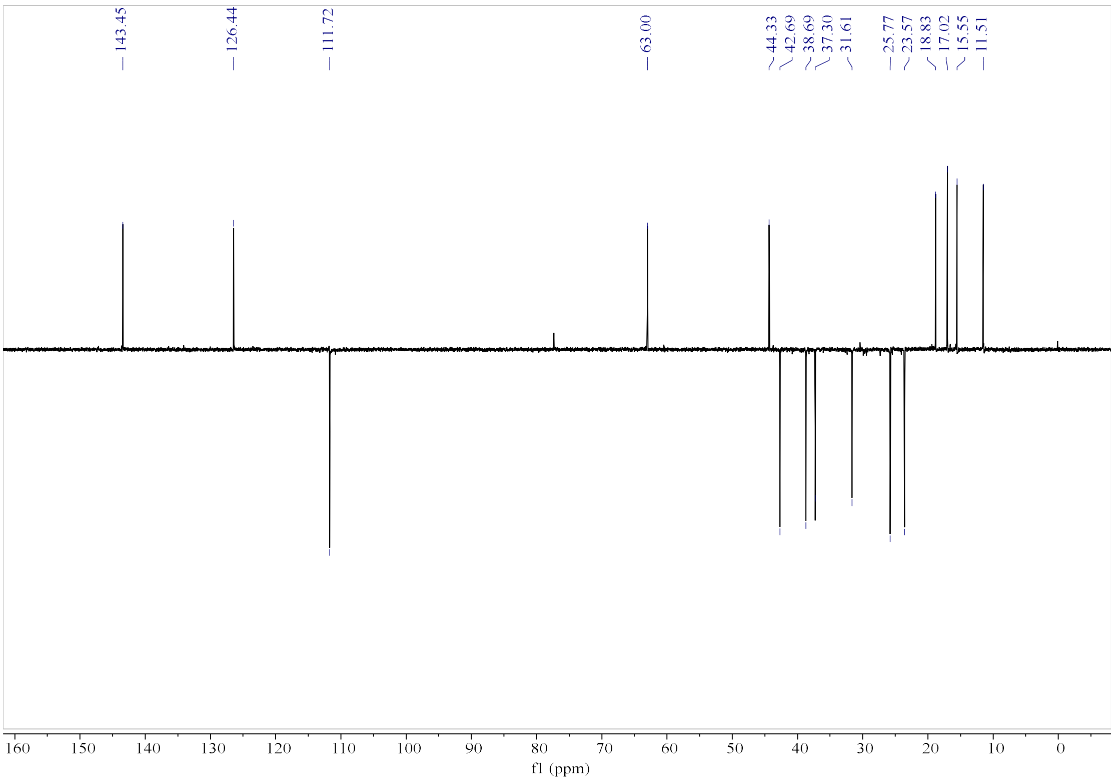

Figure S45 DEPT 135 spectrum of **5** in $CDCl_3$, 150 MHz

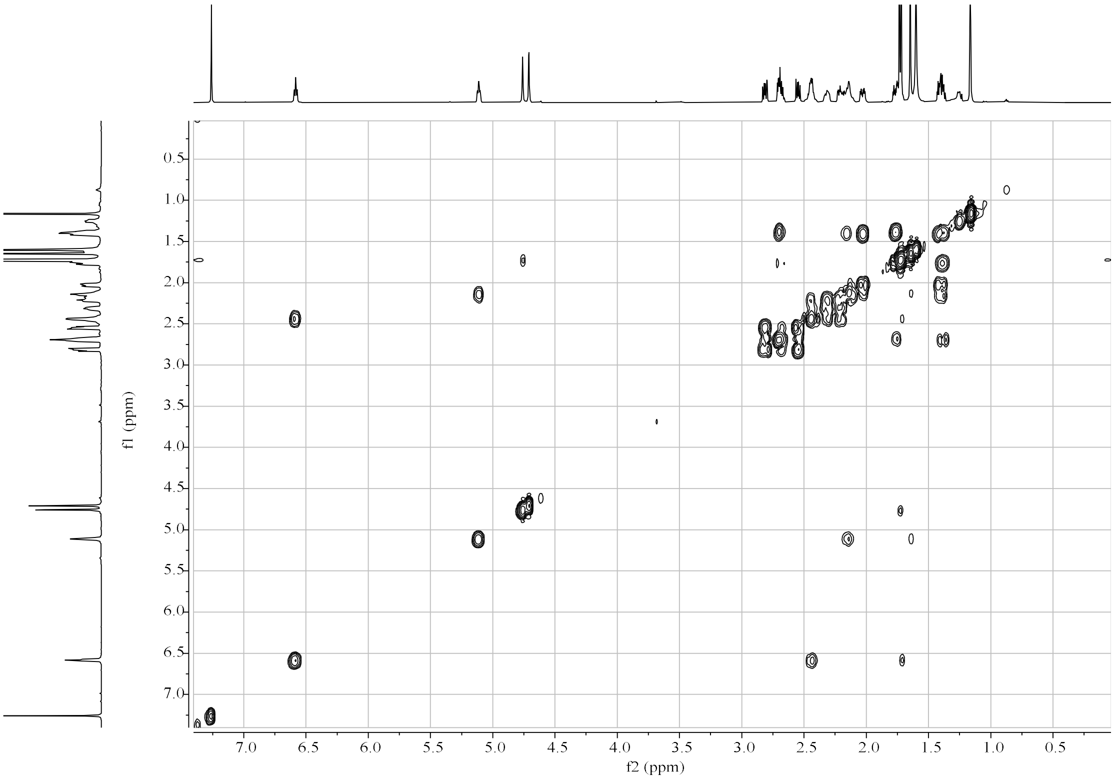

Figure S46 ^{1}H-^{1}H COSY spectrum of **5** in $CDCl_3$, 600 MHz

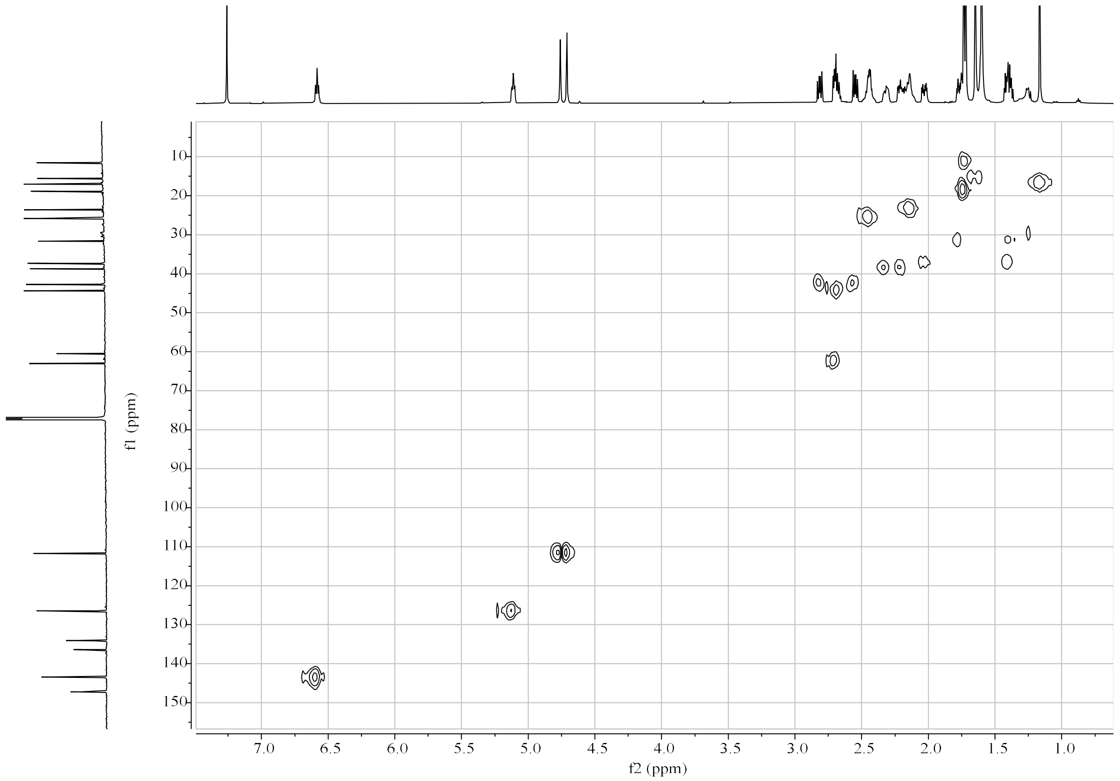

Figure S47 HSQC spectrum of **5** in $CDCl_3$, 150 MHz

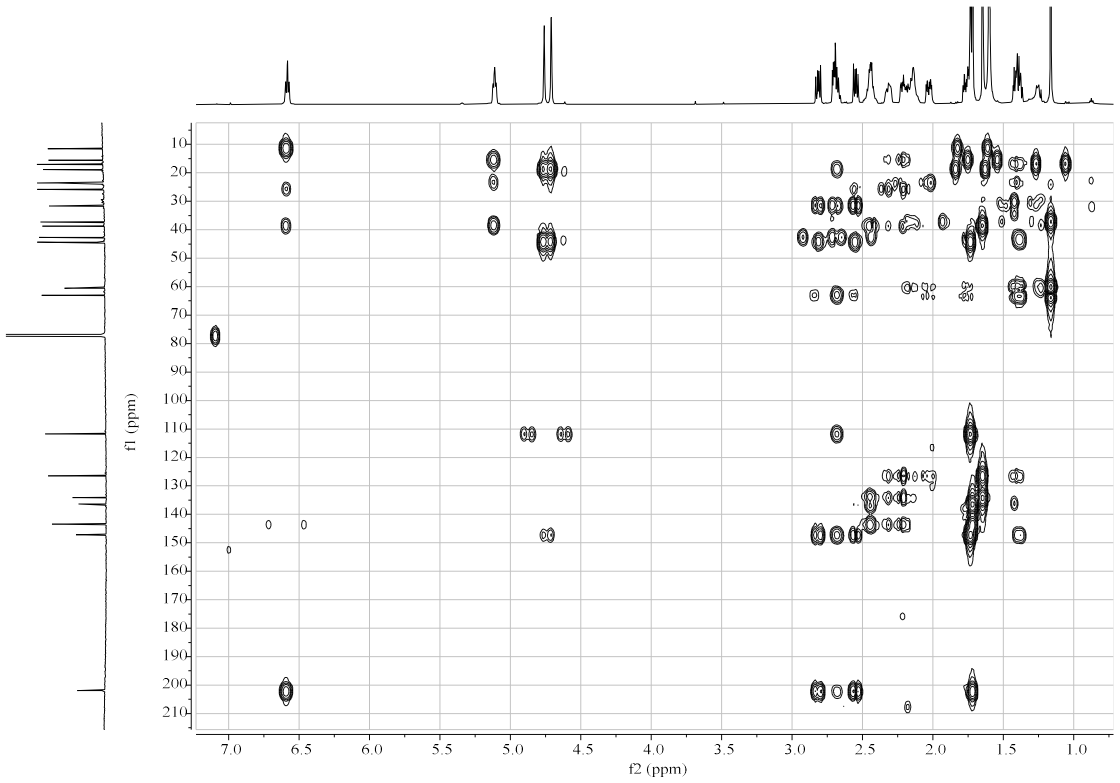

Figure S48 HMBC spectrum of **5** in $CDCl_3$, 150 MHz

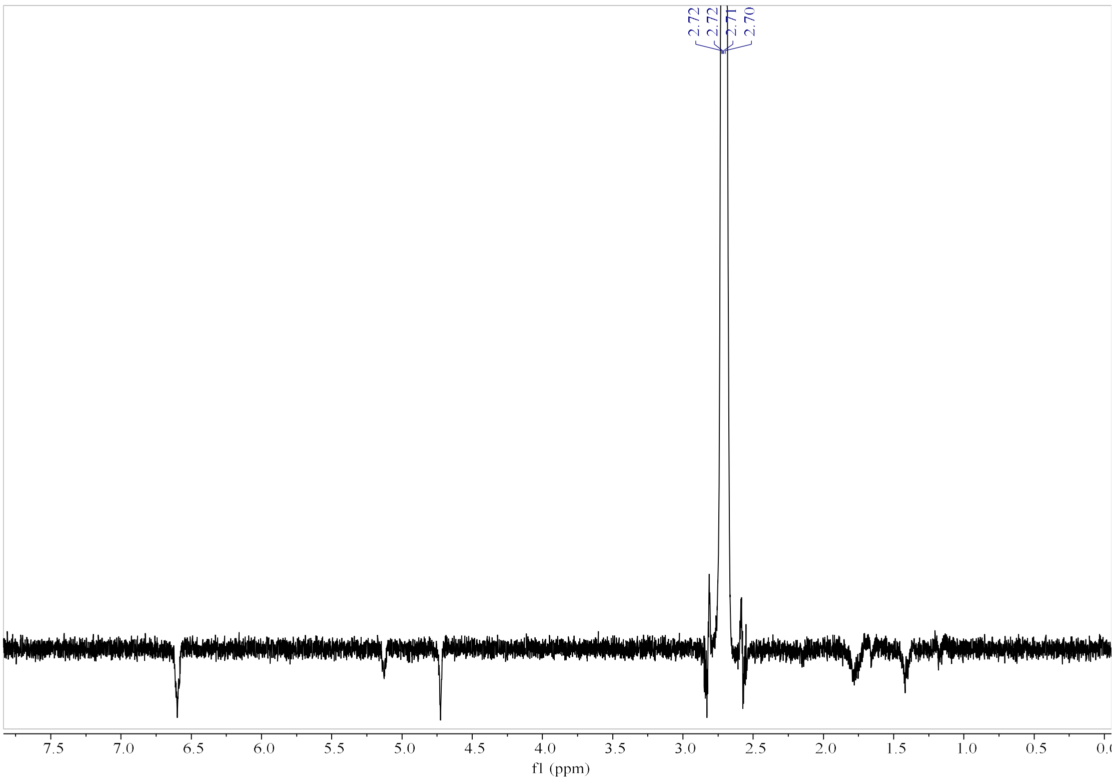

Figure S49 1D NOE spectrum of **5** in $CDCl_3$, 600 MHz (H-3)

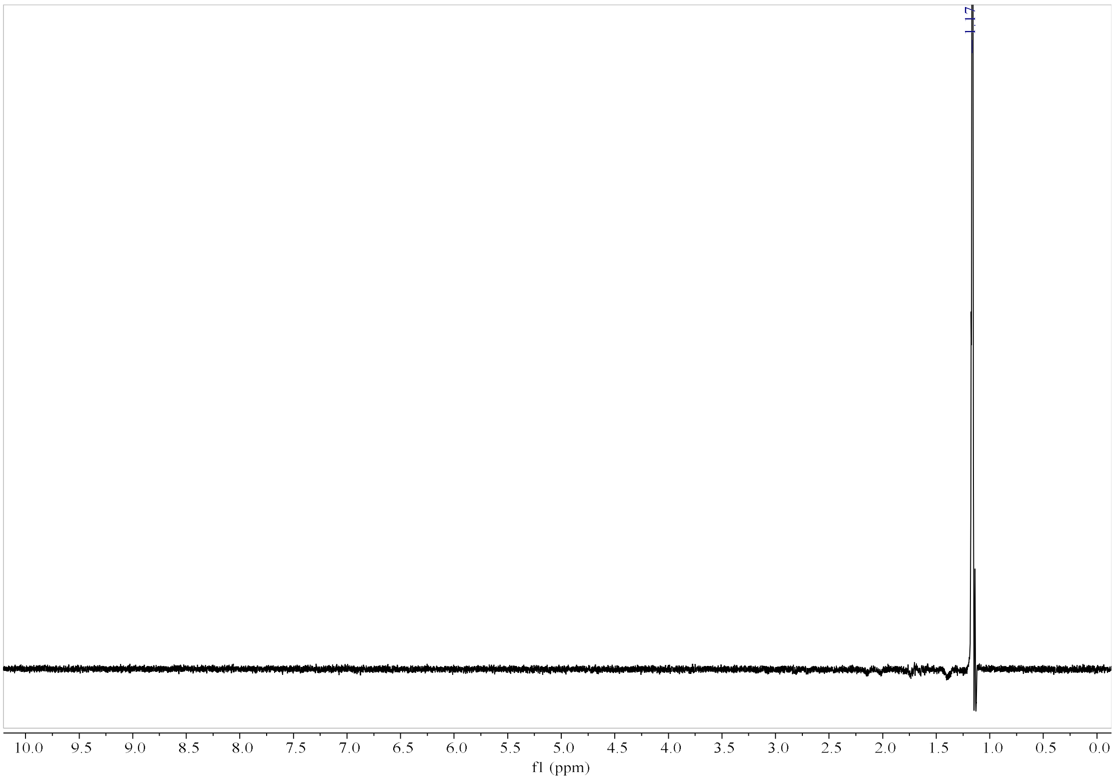

Figure S50 1D NOE spectrum of **5** in $CDCl_3$, 600 MHz (H_3-18)

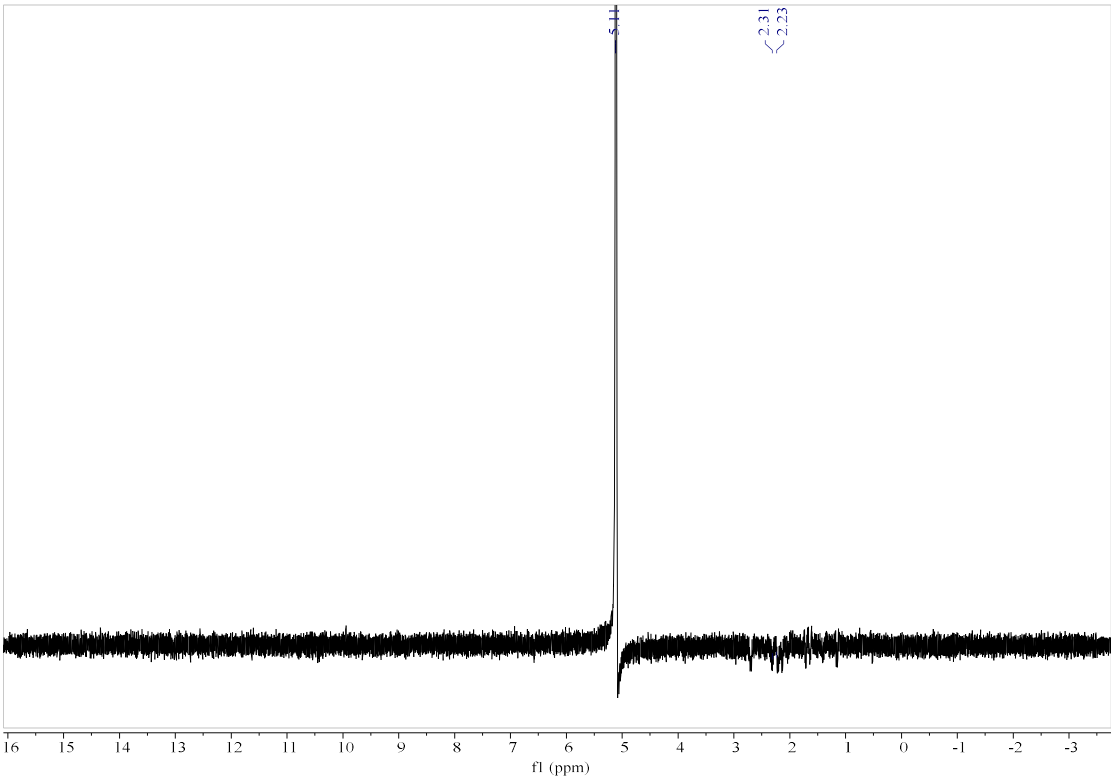

Figure S51 1D NOE spectrum of **5** in $CDCl_3$, 600 MHz (H-7)

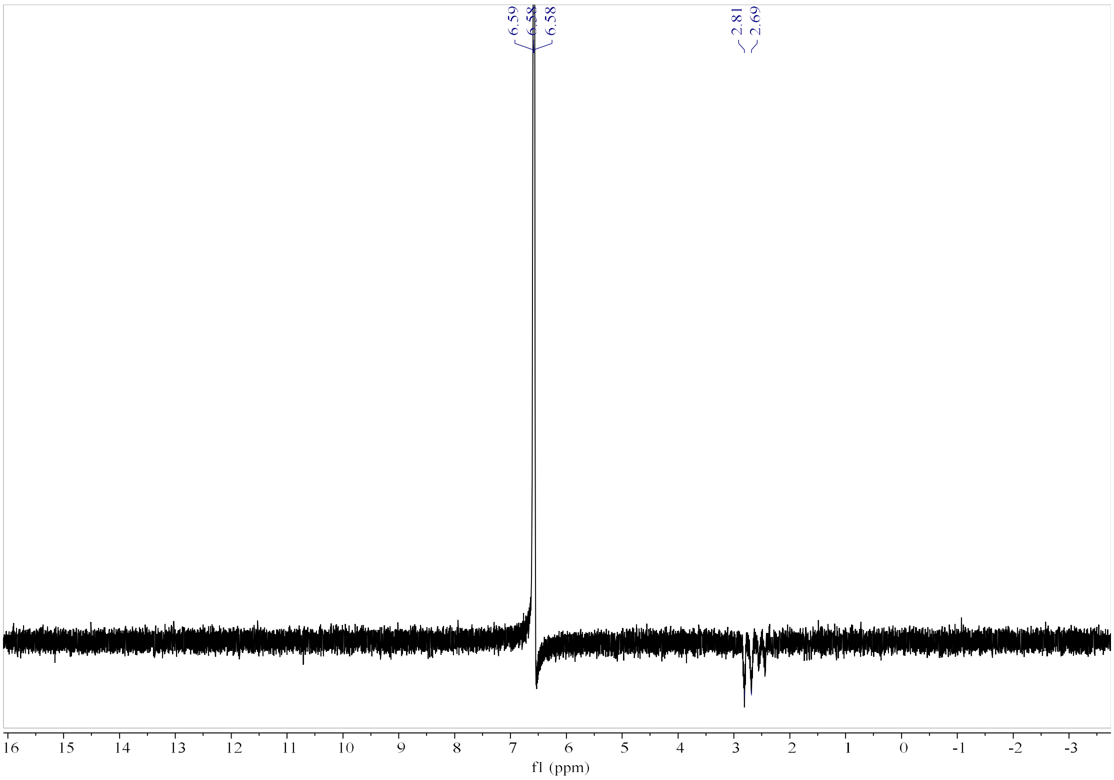

Figure S52 1D NOE spectrum of **5** in $CDCl_3$, 600 MHz (H-11)

Functional		Solvent?		Basis Set		Type of Data	
mPW1PW91		PCM		6-31+G(d, p)		Unscaled Shifts	
		DP4+	100.00%	0.00%	-	-	-
Nuclei	sp2?	Experimental	Isomer 1	Isomer 2	Isomer 3	Isomer 4	Isomer 5
C	x	201.9	201.1	201.7			
C	x	147.2	152.1	152.0			
C	x	143.5	147.5	147.6			
C	x	134.1	136.7	136.1			
C	x	136.4	136.8	138.7			
C	x	126.5	125.8	127.7			
C	x	111.7	108.9	106.4			
C		63	65.7	61.4			
C		60.5	62.1	60.5			
C		44.4	49.8	46.5			
C		42.7	46.2	38.2			
C		38.7	42.82	42.78			
C		37.3	40.40	42.40			
C		25.8	31.15	30.25			
C		31.6	31.57	32.20			
C		23.6	28.30	28.54			
C		18.9	18.89	24.77			
C		17	17.45	17.64			
C		15.6	16.56	15.63			
C		11.5	14.52	14.59			

Functional	Solvent?		Basis Set		Type of Data	
mPW1PW91	PCM		6-31+G(d, p)		Unscaled Shifts	
	Isomer 1	Isomer 2	Isomer 3	Isomer 4	Isomer 5	Isomer 6
sDP4+ (H data)	-	-	-	-	-	-
sDP4+ (C data)	99.98%	0.02%	-	-	-	-
sDP4+ (all data)	99.98%	0.02%	-	-	-	-
uDP4+ (H data)	-	-	-	-	-	-
uDP4+ (C data)	100.00%	0.00%	-	-	-	-
uDP4+ (all data)	100.00%	0.00%	-	-	-	-
DP4+ (H data)	-	-	-	-	-	-
DP4+ (C data)	100.00%	0.00%	-	-	-	-
DP4+ (all data)	100.00%	0.00%	-	-	-	-

Figure S53 Detailed $DP4^+$ probability (calculated at PCM/mPW1PW91/6-31+G (d, p) level) for compound **5**. Isomer **1** is 1*R*, 3*S*,4*S*, isomer **2** is 1*R*, 3*R*,4*R*

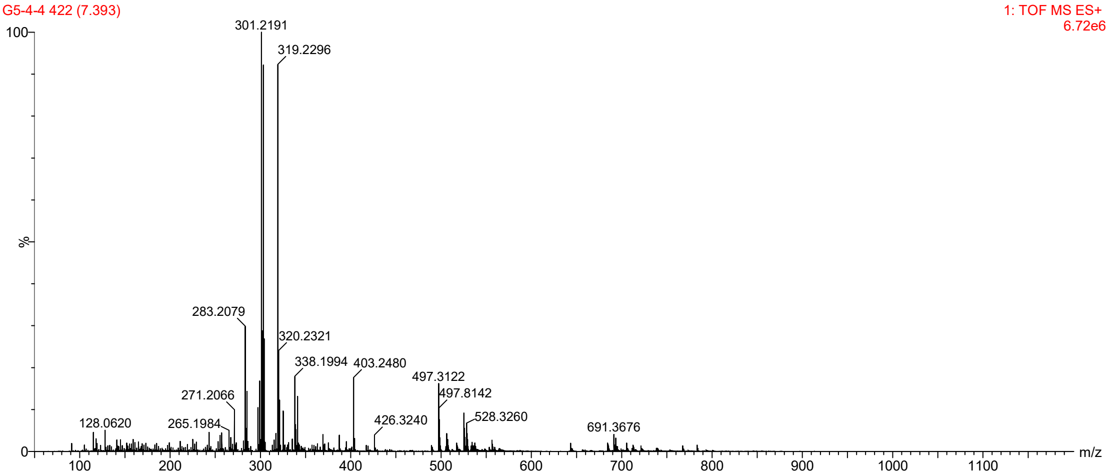

Figure S54 HRESIMS spectrum of compound **6**

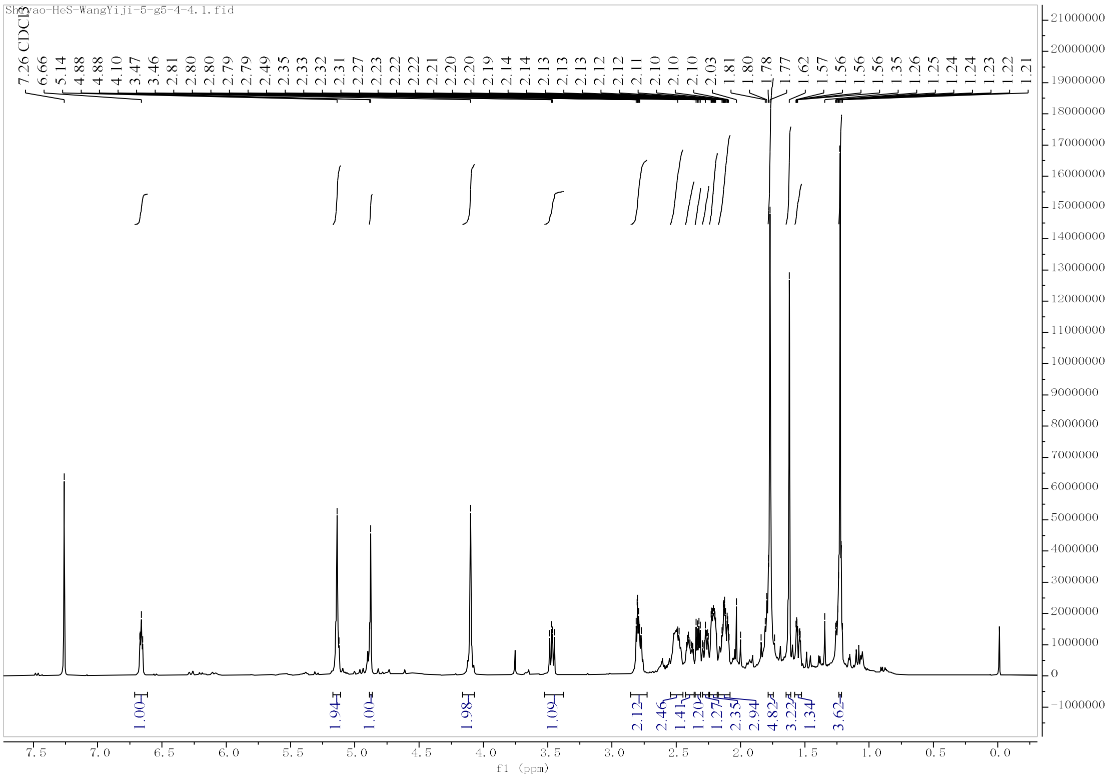

Figure S55 ^{1}H NMR spectrum of compound **6** in $CDCl_3$, 600 MHz

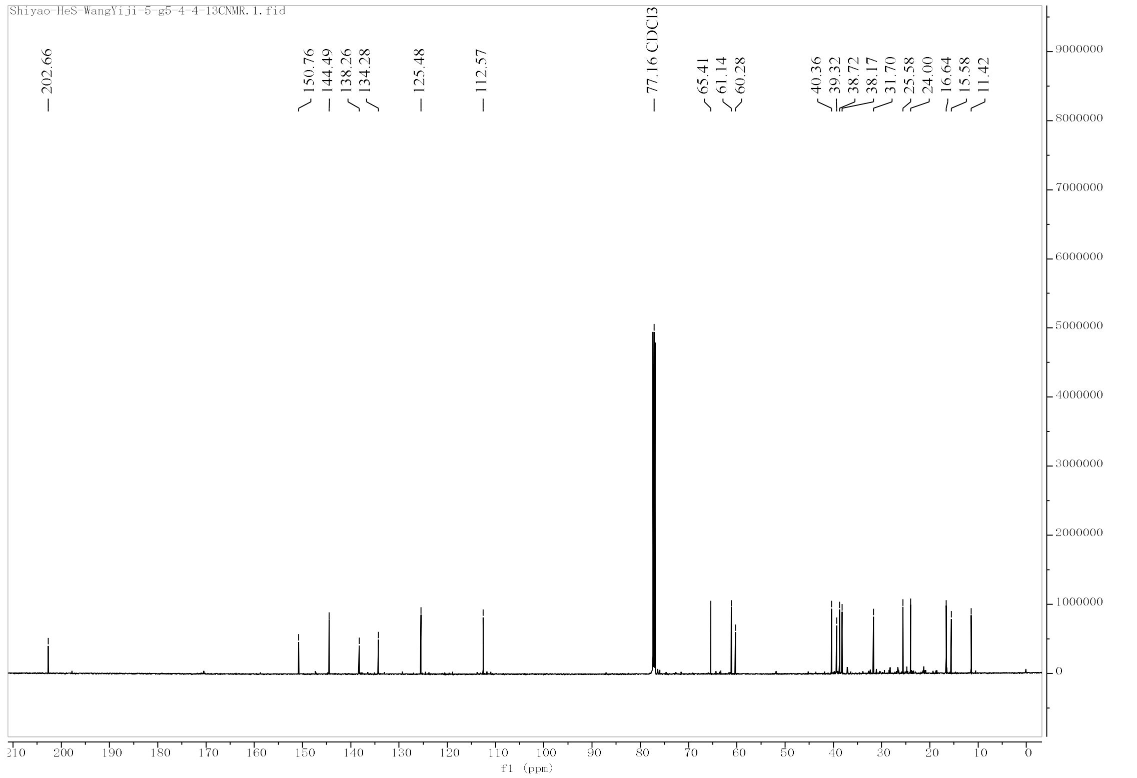

Figure S56 ^{13}C NMR spectrum of compound **6** in $CDCl_3$, 150 MHz

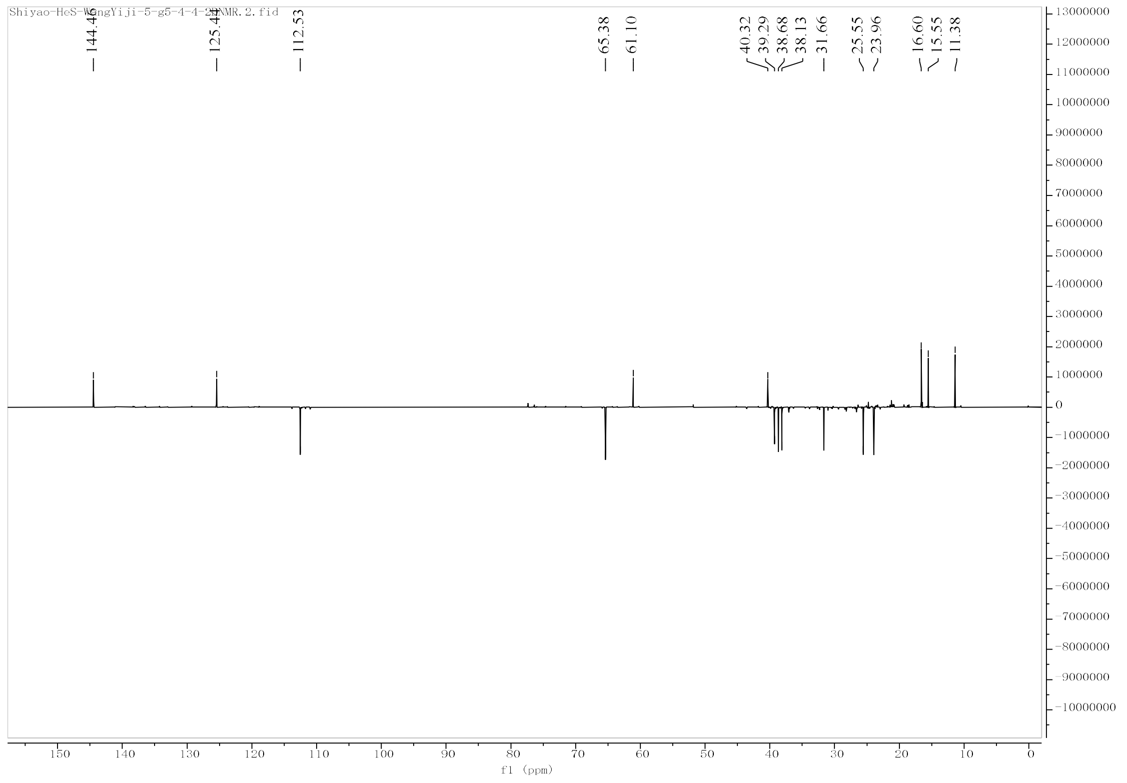

Figure S57 DEPT 135 spectrum of **6** in $CDCl_3$, 150 MHz

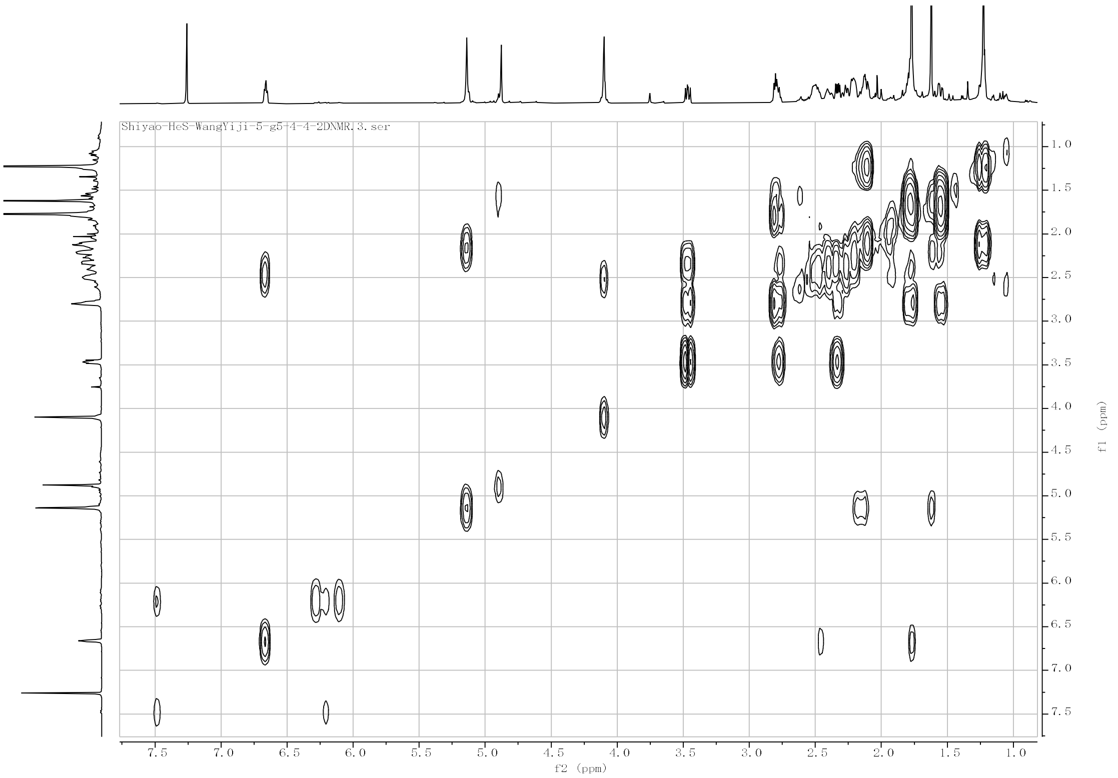

Figure S58 ^{1}H-^{1}H COSY spectrum of **6** in $CDCl_3$, 600 MHz

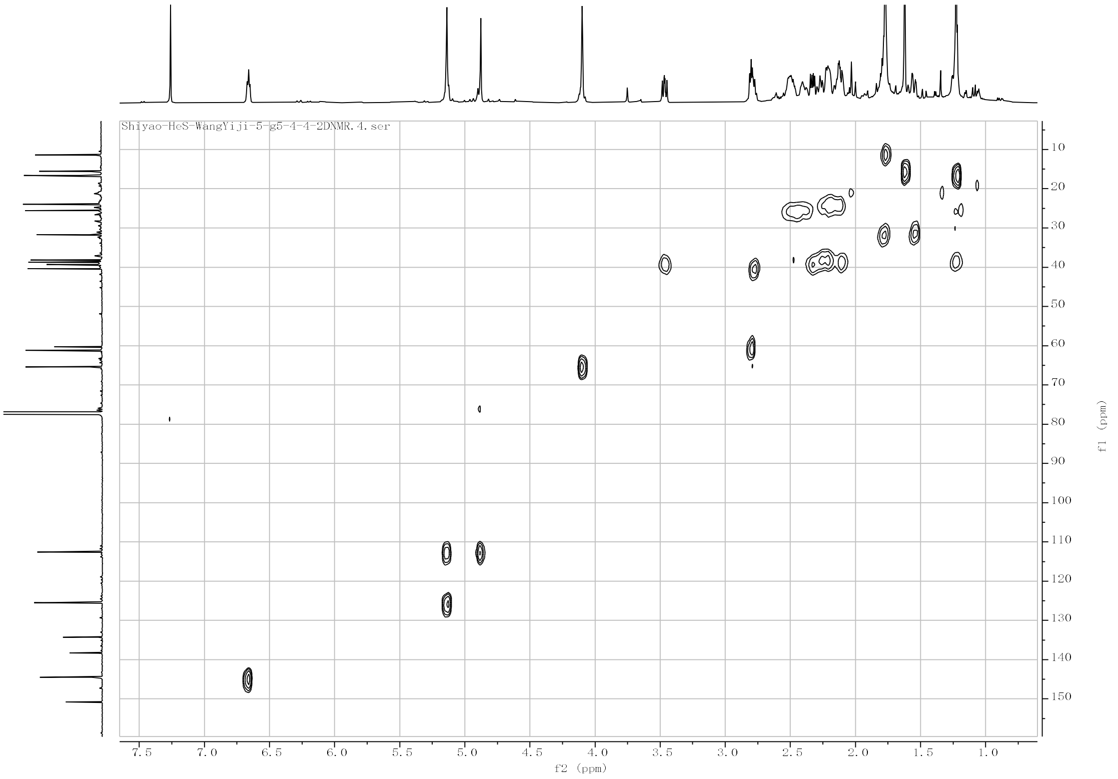

Figure S59 HSQC spectrum of **6** in $CDCl_3$, 150 MHz

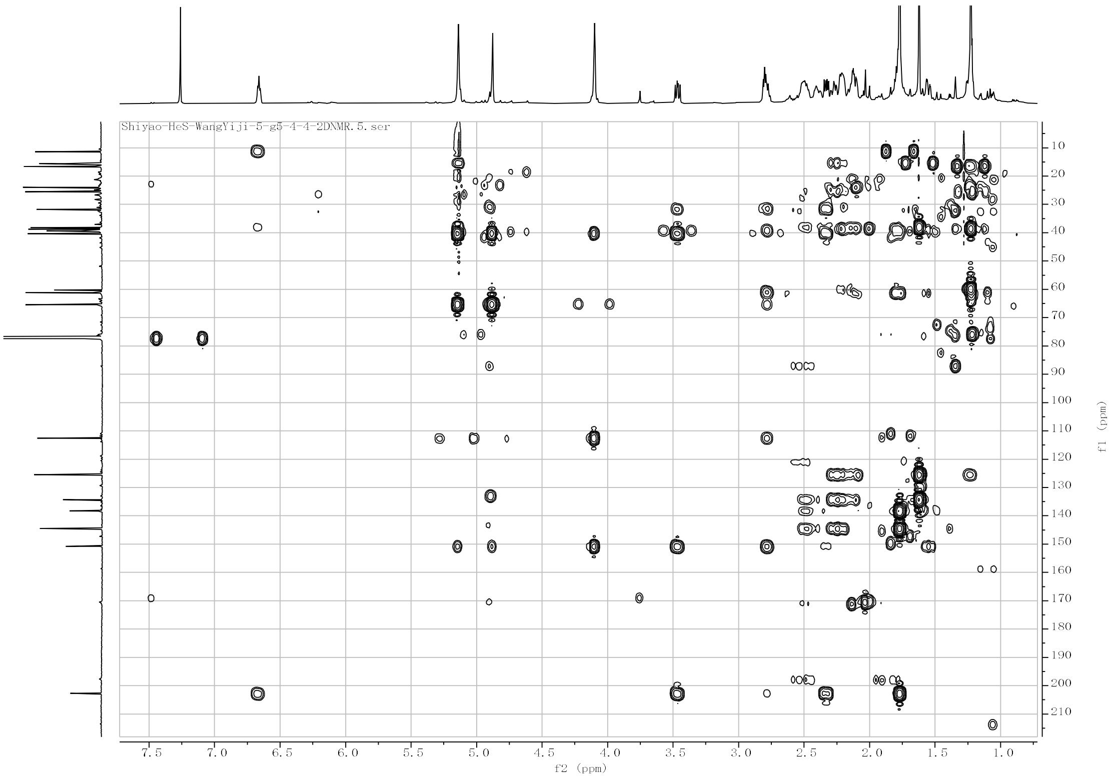

Figure S60 HMBC spectrum of **6** in $CDCl_3$, 150 MHz

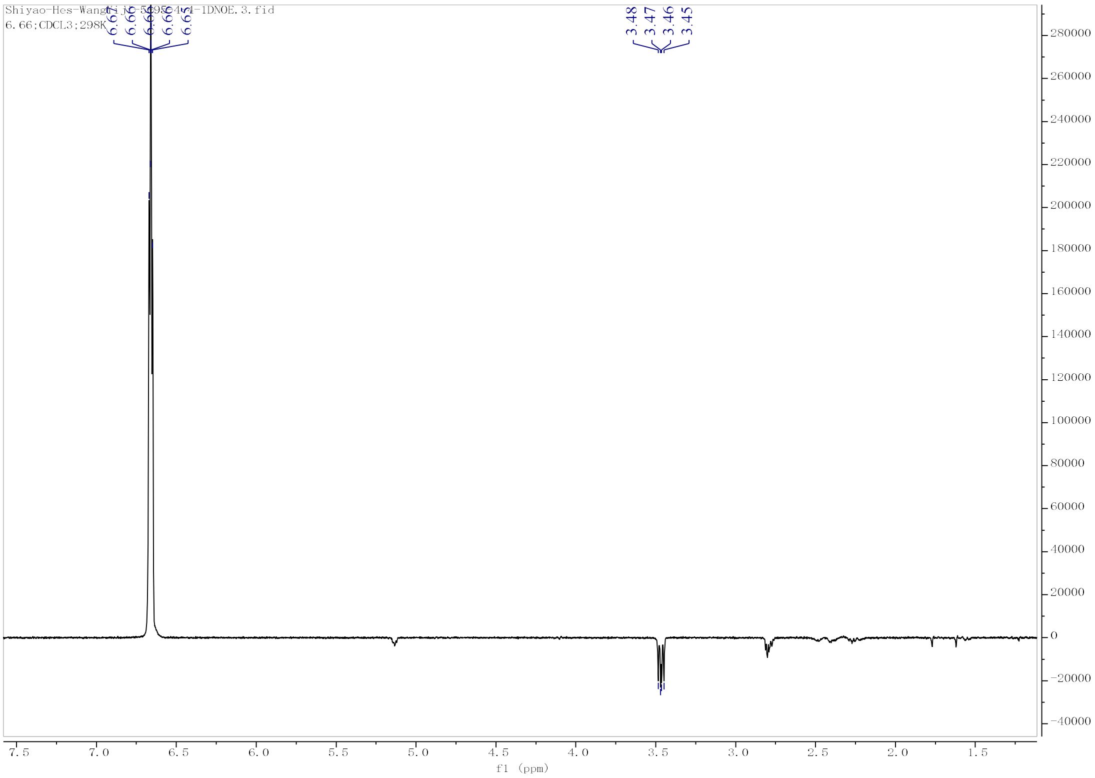

Figure S61 1D NOE spectrum of **6** in $CDCl_3$, 600 MHz (H-11)

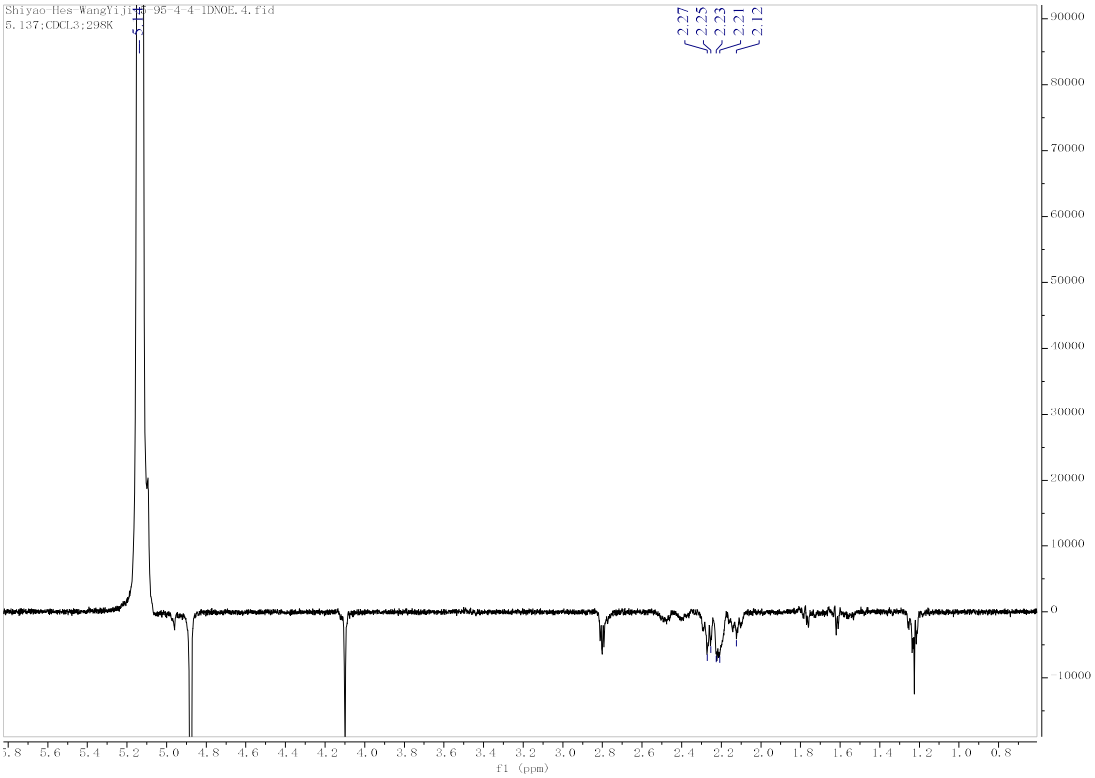

Figure S62 1D NOE spectrum of **6** in $CDCl_3$, 600 MHz (H-7)

Functional		Solvent?		Basis Set	Type of Data	
mPW1PW91		PCM		6-31+G(d, p)	Unscaled Shifts	
		DP4+	0.04%	0.01%	0.00%	99.95%	-
Nuclei	sp2?	Experimenta	Isomer 1	Isomer 2	Isomer 3	Isomer 4	Isomer 5
C	x	202.7	209.4	211.1	207.6	204.3	
C	x	150.8	156.5	153.6	158.4	159.7	
C	x	144.5	150.8	151.5	149.9	146.0	
C	x	138.3	137.5	135.6	140.7	139.3	
C	x	134.3	136.9	136.3	138.5	137.4	
C	x	125.5	129.6	134.2	126.7	127.9	
C	x	112.6	116.2	114.8	107.6	110.6	
C		60.3	73.2	72.6	70.1	71.8	
C		61.1	71.9	69.7	73.0	70.2	
C		65.4	62.8	69.8	67.3	70.8	
C		40.4	52.4	53.5	49.8	41.8	
C		39.3	42.19	42.89	46.22	44.38	
C		38.7	42.39	38.30	37.80	33.27	
C		38.2	41.23	41.50	44.86	43.44	
C		31.7	37.33	35.74	36.13	37.03	
C		25.6	29.35	29.32	31.33	31.56	
C		24	28.86	25.36	28.28	28.15	
C		16.6	17.22	21.90	23.23	23.12	
C		15.6	16.45	16.34	16.09	16.81	
C		11.4	14.57	14.07	14.24	14.74	

Functional		Solvent?		Basis Set		Type of Data	
mPW1PW91		PCM		6-31+G(d,p)		Unscaled Shifts	

	Isomer 1	Isomer 2	Isomer 3	Isomer 4	Isomer 5	Isomer 6
sDP4+ (H data)	-	-	-	-	-	-
sDP4+ (C data)	21.57%	0.46%	0.87%	77.10%	-	-
sDP4+ (all data)	21.57%	0.46%	0.87%	77.10%	-	-
uDP4+ (H data)	-	-	-	-	-	-
uDP4+ (C data)	0.15%	1.10%	0.00%	98.75%	-	-
uDP4+ (all data)	0.15%	1.10%	0.00%	98.75%	-	-
DP4+ (H data)	-	-	-	-	-	-
DP4+ (C data)	0.04%	0.01%	0.00%	99.95%	-	-
DP4+ (all data)	0.04%	0.01%	0.00%	99.95%	-	-

Figure S63 Detailed DP4+ probability (calculated at PCM/mPW1PW91/6-31+G (d, p) level) for compound **6**. Isomer **1** is 1*S*,3*S*,4*S*, isomer **2** is 1*S*,3*R*,4*R*, isomer **3** is 1*S*,3*R*,4*S*, isomer **4** is 1*S*,3*S*,4*R*

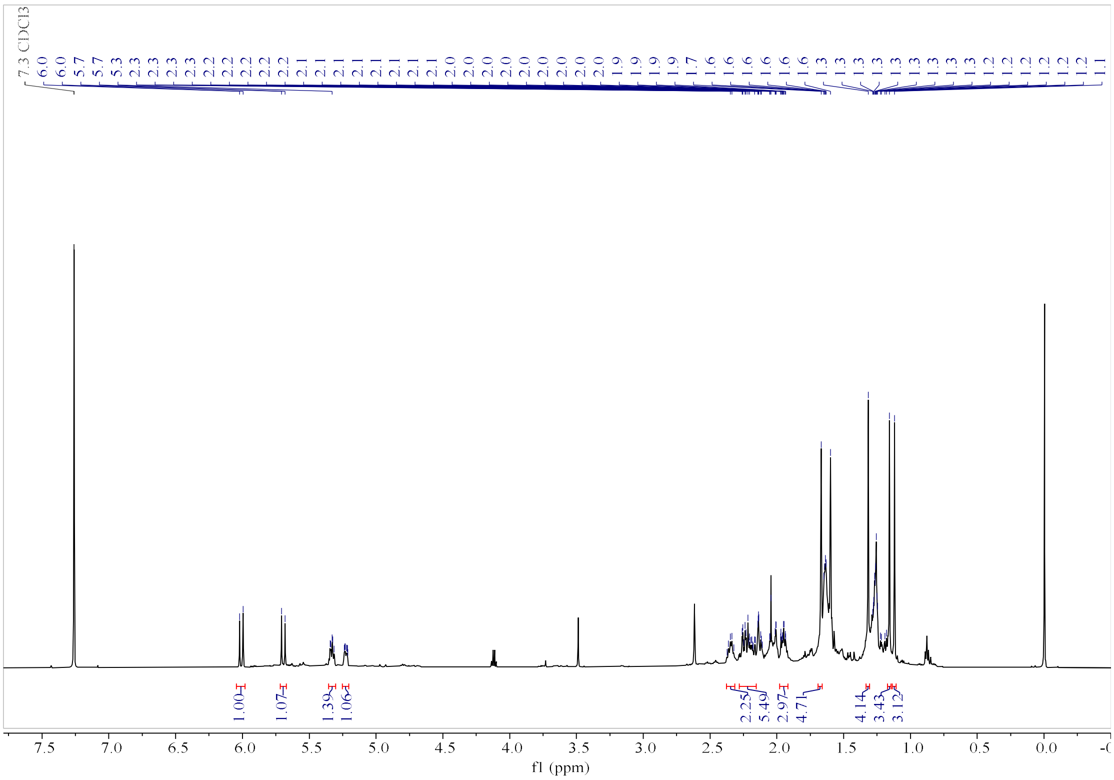

Figure S64 ^{1}H NMR spectrum of compound **7** in $CDCl_3$, 600 MHz

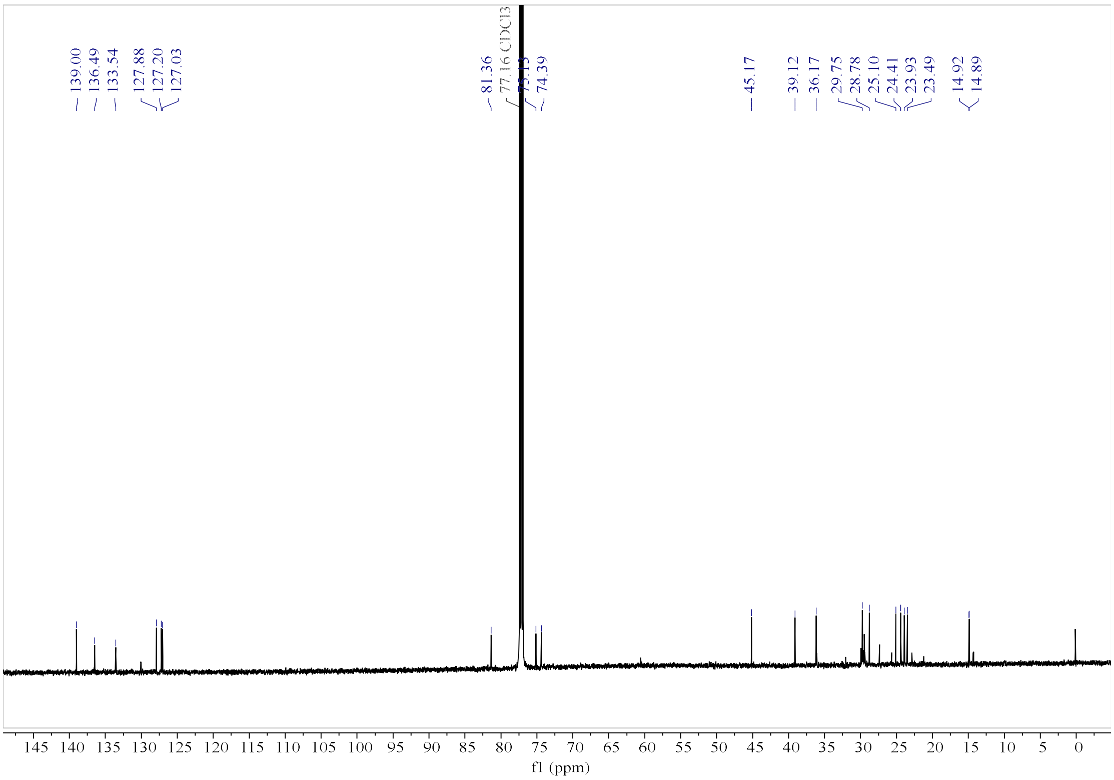

Figure S65 ^{13}C NMR spectrum of compound **7** in $CDCl_3$, 150 MHz

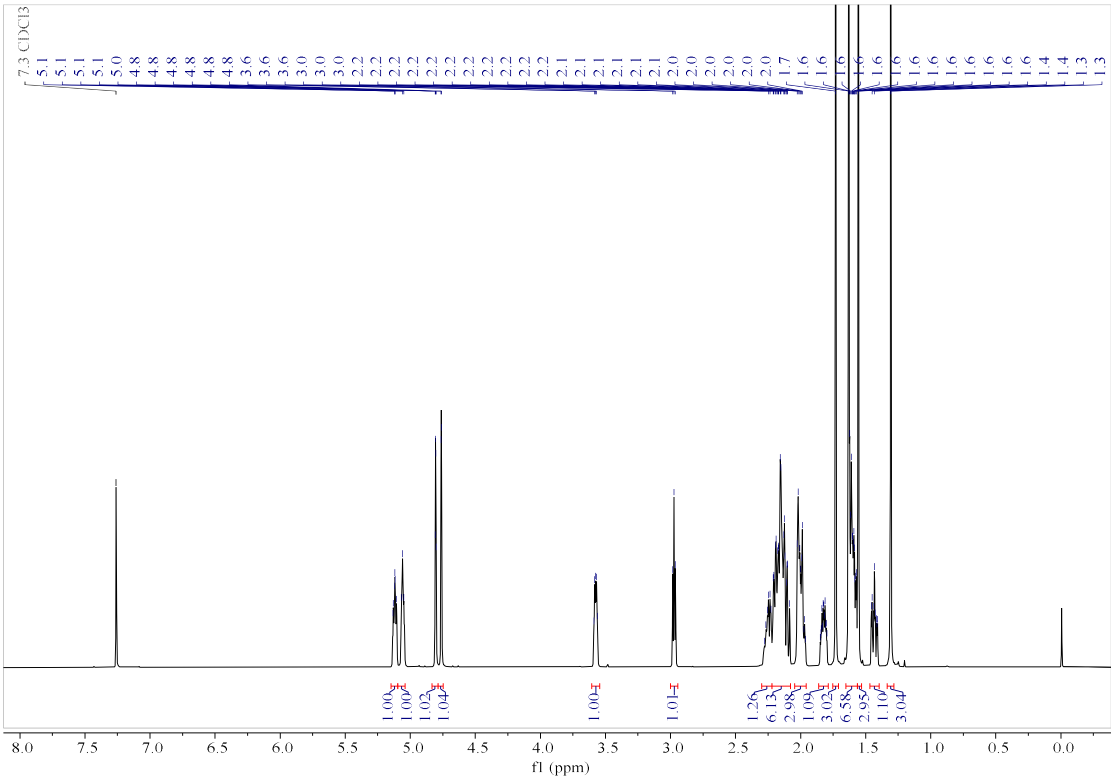

Figure S66 ^{1}H NMR spectrum of compound **8** in $CDCl_3$, 600 MHz

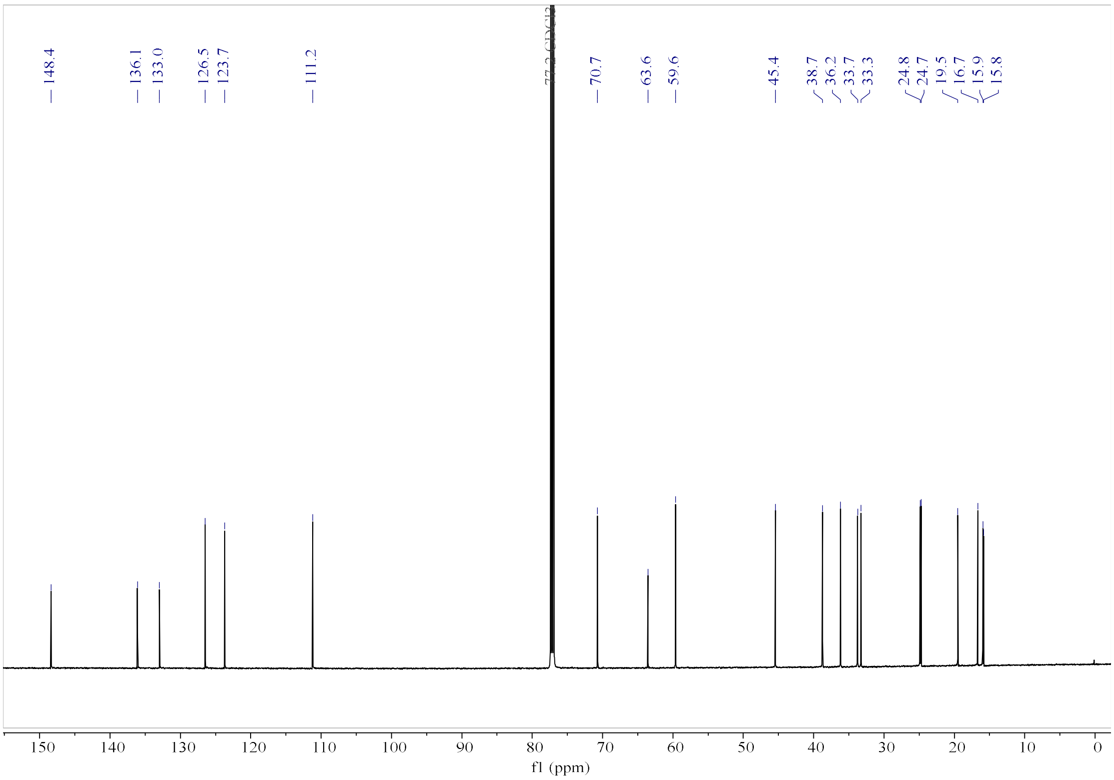

Figure S67 ^{13}C NMR spectrum of compound **8** in $CDCl_3$, 150 MHz

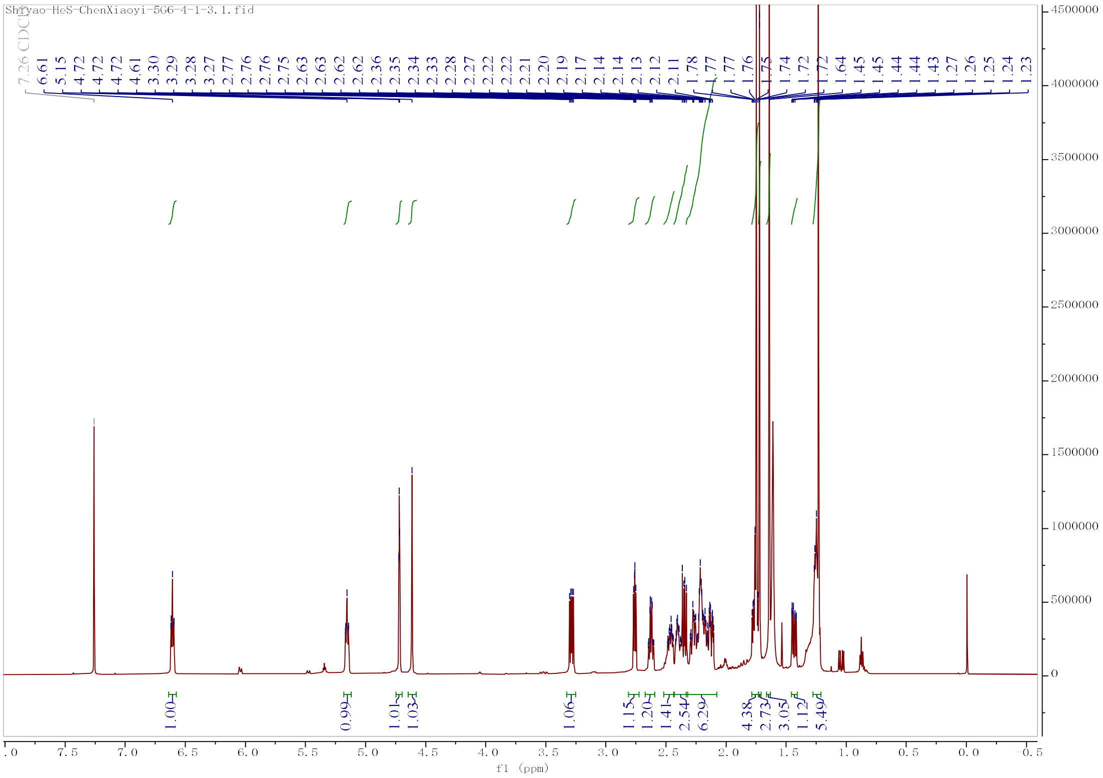

Figure S68 ^{1}H NMR spectrum of compound **9** in $CDCl_3$, 600 MHz

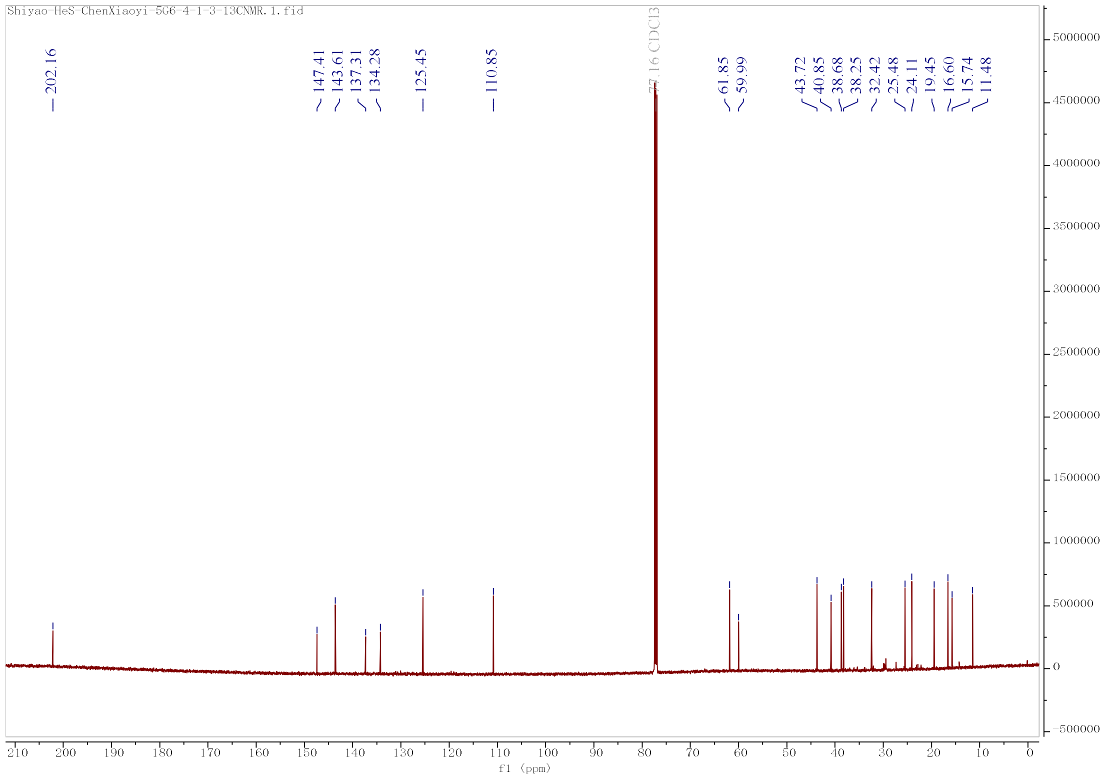

Figure S69 ^{13}C NMR spectrum of compound **9** in $CDCl_3$, 150 MHz
